# Population Immunity against Serotype-2 Poliomyelitis Leading up to the Global Withdrawal of the Oral Poliovirus Vaccine: Spatio-temporal Modelling of Surveillance Data

**DOI:** 10.1371/journal.pmed.1002140

**Published:** 2016-10-04

**Authors:** Margarita Pons-Salort, Natalie A. Molodecky, Kathleen M. O’Reilly, Mufti Zubair Wadood, Rana M. Safdar, Andrew Etsano, Rui Gama Vaz, Hamid Jafari, Nicholas C. Grassly, Isobel M. Blake

**Affiliations:** 1 Department of Infectious Disease Epidemiology, St Mary’s Campus, Imperial College London, London, United Kingdom; 2 World Health Organization, Islamabad, Pakistan; 3 National Emergency Operation Centre, Ministry of National Health Services, Regulations and Coordination, Islamabad, Pakistan; 4 National Primary Health Care Development Agency, Abuja, Nigeria; 5 World Health Organization, Abuja, Nigeria; 6 World Health Organization, Geneva, Switzerland; National Institutes of Health, UNITED STATES

## Abstract

**Background:**

Global withdrawal of serotype-2 oral poliovirus vaccine (OPV2) took place in April 2016. This marked a milestone in global polio eradication and was a public health intervention of unprecedented scale, affecting 155 countries. Achieving high levels of serotype-2 population immunity before OPV2 withdrawal was critical to avoid subsequent outbreaks of serotype-2 vaccine-derived polioviruses (VDPV2s).

**Methods and Findings:**

In August 2015, we estimated vaccine-induced population immunity against serotype-2 poliomyelitis for 1 January 2004–30 June 2015 and produced forecasts for April 2016 by district in Nigeria and Pakistan. Population immunity was estimated from the vaccination histories of children <36 mo old identified with non-polio acute flaccid paralysis (AFP) reported through polio surveillance, information on immunisation activities with different oral poliovirus vaccine (OPV) formulations, and serotype-specific estimates of the efficacy of these OPVs against poliomyelitis. District immunity estimates were spatio-temporally smoothed using a Bayesian hierarchical framework. Coverage estimates for immunisation activities were also obtained, allowing for heterogeneity within and among districts. Forward projections of immunity, based on these estimates and planned immunisation activities, were produced through to April 2016 using a cohort model.

Estimated population immunity was negatively correlated with the probability of VDPV2 poliomyelitis being reported in a district. In Nigeria and Pakistan, declines in immunity during 2008–2009 and 2012–2013, respectively, were associated with outbreaks of VDPV2. Immunity has since improved in both countries as a result of increased use of trivalent OPV, and projections generally indicated sustained or improved immunity in April 2016, such that the majority of districts (99% [95% uncertainty interval 97%–100%] in Nigeria and 84% [95% uncertainty interval 77%–91%] in Pakistan) had >70% population immunity among children <36 mo old. Districts with lower immunity were clustered in northeastern Nigeria and northwestern Pakistan. The accuracy of immunity estimates was limited by the small numbers of non-polio AFP cases in some districts, which was reflected by large uncertainty intervals. Forecasted improvements in immunity for April 2016 were robust to the uncertainty in estimates of baseline immunity (January–June 2015), vaccine coverage, and vaccine efficacy.

**Conclusions:**

Immunity against serotype-2 poliomyelitis was forecasted to improve in April 2016 compared to the first half of 2015 in Nigeria and Pakistan. These analyses informed the endorsement of OPV2 withdrawal in April 2016 by the WHO Strategic Advisory Group of Experts on Immunization.

## Abstract 2

Isobel Mary Blake and colleagues use spatio-temporal modelling of surveillance data to estimate population immunity leading up to the global withdrawal of serotype-2 oral poliovirus vaccine.

## Author Summary

**Why Was This Study Done?:**

- The Global Polio Eradication Initiative’s strategic plan includes the global withdrawal of all oral poliovirus vaccines (OPVs) because they can result, albeit rarely, in outbreaks of poliomyelitis caused by vaccine-derived poliovirus (VDPV).
- Serotype-2 OPV (OPV2) was withdrawn from trivalent OPV in all 155 countries using this vaccine in April 2016, as the last naturally occurring case of polio caused by serotype-2 wild poliovirus was reported in 1999.
- Strategies to minimise the risk of serotype-2 VDPVs during the period leading up to OPV2 withdrawal were required.

**What Did the Researchers Do and Find?:**

- To assess the risks associated with OPV2 withdrawal, we estimated population immunity against poliomyelitis caused by serotype 2 in children under three years old in eight countries (including the two highest risk countries: Nigeria and Pakistan).
- We found substantial improvements in serotype-2 population immunity over the last five years in most countries, although immunity in parts of northeastern Nigeria and northwestern Pakistan remained relatively low.
- Projections of immunity through April 2016 were used to explore the impact of alternative immunisation campaigns including the use of inactivated poliovirus vaccine.

**What Do These Findings Mean?:**

- An increased number of campaigns with trivalent OPV and IPV led to improvements in serotype-2 immunity, thereby minimising the risk of serotype-2 VDPVs associated with OPV2 withdrawal in April 2016.
- These immunity estimates and projections were used to support the decision by the World Health Organization Strategic Advisory Group of Experts on Immunization to proceed with OPV2 withdrawal.

## Introduction

A key milestone towards polio eradication is the global withdrawal of all live-attenuated oral poliovirus vaccines (OPVs) [1]. OPVs have played an instrumental role in the Global Polio Eradication Initiative (GPEI), and their use has largely contributed to the >99% reduction of annual poliomyelitis cases since the start of the programme in 1988 [1]. Historically, the GPEI has recommended the use of OPV—mainly trivalent OPV (tOPV), which protects against all three poliovirus serotypes—because of its low cost, ease of administration (oral), ability to induce a strong intestinal mucosal immune response, and potential to indirectly immunise secondary contacts. Until April 2016, tOPV was used in 155 countries, primarily through routine immunisation (RI) programmes [1] (administering three doses most commonly at 6, 10, and 14 wk) and additionally in mass supplementary immunisation activities (SIAs) to increase population immunity in settings where there is poliovirus transmission and RI coverage is low. Although OPV has served the GPEI well, the vaccine is genetically unstable, and its use carries some risks [2]. On very rare occasions, OPV may cause vaccine-associated paralytic poliomyelitis (VAPP), at a low rate of about 1–2 cases of VAPP per million primary immunisations [3]. Moreover, OPV viruses shed from vaccinees and their contacts may lose their attenuating mutations, regain transmissibility and neurovirulence, and result in outbreaks of vaccine-derived poliovirus (VDPV) poliomyelitis similar to those observed for wild polioviruses [4].

VDPVs are OPV-related isolates whose VP1 capsid protein has diverged from the OPV parental strain by >1% for serotypes 1 and 3 and >0.6% for serotype 2 [5]. VDPVs are classified as circulating VDPVs (cVDPVs) when there is evidence of person-to-person transmission, immunodeficiency-associated VDPVs (iVDPVs) when they are shed by individuals with an immunodeficiency (sometimes chronically excreted for years), and ambiguous VDPVs (aVDPVs) when there is insufficient evidence to classify them as cVDPV or iVDPV [6,7]. With the aim of improving the sensitivity of surveillance, the definition of cVDPVs was updated in July 2015 and no longer requires that “genetically linked VDPVs are isolated from at least two AFP cases”. From 1 January 2000 to 27 October 2015, 790 cases of cVDPV-associated poliomyelitis were reported [8], with serotype-2 vaccine-derived poliovirus (VDPV2) accounting for >85% of these cases [8]. Given the large number of circulating VDPV2 (cVDPV2) cases and that serotype-2 wild poliovirus (WPV2) was eradicated in 1999 [9,10], global withdrawal of serotype-2 OPV (OPV2) was of particular urgency.

Given the risk of VAPP and cVDPVs, the GPEI’s endgame strategy plans for the global transition from OPV to inactivated poliovirus vaccine (IPV). Like tOPV, IPV protects against poliomyelitis caused by all three serotypes, but it does not induce a strong mucosal immune response on its own [11] and therefore is likely to have a small effect in preventing poliovirus transmission. The globally synchronised removal of the three serotypes of OPV started with OPV2. In April 2016, tOPV was replaced with bivalent OPV (bOPV) (protecting against serotypes 1 and 3) during a 2-wk window in the 155 countries that were using OPV in RI. Furthermore, tOPV is no longer used for supplementary immunisations since that time [12]. The global vaccine switch will lead to a decline in population immunity to serotype 2 over time, which will leave populations at risk of VDPV2 outbreaks if they are then exposed to VDPV2 (from silent circulation at the time of the switch, from new VDPV2 emergence, or through inadvertent use of OPV2 after the switch). In order to reduce the risk of VDPV2 outbreaks after OPV2 withdrawal, the GPEI conducted large-scale SIAs with tOPV in high-risk countries prior to the tOPV-bOPV switch (with additional SIAs using IPV in Pakistan and Afghanistan). Additionally, before the switch, the GPEI recommended that all countries using tOPV in RI introduce at least one dose of IPV into RI programmes by the end of 2015 [13], to be co-administered with the third dose of OPV at ≥14 wk of age.

Nigeria and Pakistan have experienced the highest number of cVDPV2 cases in recent years [8], and immunity to serotype 2 is likely to have decreased at some point during the last decade in both countries. Following WPV2 elimination, new formulations of OPV were developed to increase protective efficacy against serotypes 1 and 3, including monovalent OPV1 (mOPV1) and mOPV3, licensed in 2005, and bOPV, licensed in 2009 [14–17]. These new formulations have been used in SIAs during the last decade to accelerate elimination of the two remaining wild types, in some instances leading to a decline in the use of tOPV in SIAs, in particular in countries with sustained transmission of serotypes 1 and 3, such as Nigeria and Pakistan. Analysing the trends of serotype-2 population immunity and concomitant incidence of cVDPV2 poliomyelitis cases in Nigeria and Pakistan provides an opportunity to better understand the risk of cVDPV2 associated with low serotype-2 population immunity before and after the switch from tOPV to bOPV.

In October 2015, the WHO Strategic Advisory Group of Experts on Immunisation (SAGE) met and endorsed the unprecedented global switch from tOPV to bOPV in April 2016 [18]. The decision to conduct the switch was based upon the perceived risk of persistent cVDPV2 circulation. The evidence informing this evaluation included the current global epidemiology of cVDPV2, surveillance quality, current serotype-2 population immunity estimates, and planned SIAs (to interrupt circulation of cVDPV2 by April 2016 and avoid new emergences before and after the switch). Nigeria and Pakistan were of particular importance for consideration given the persistent cVDPV2 circulation in 2014 (reporting of cases >6 mo after the first reported case). In advance of the SAGE meeting, we estimated population immunity to serotype-2 poliomyelitis in Nigeria and Pakistan from 2004 to the first half of 2015 at the subnational level. We then projected serotype-2 population immunity at the time of the planned tOPV-bOPV switch (April 2016) in both countries, given the estimates in the first half of 2015 and planned SIAs, to identify areas that could have low levels of population immunity at the time of the switch and therefore could require additional SIAs.

## Methods

### Data

#### Geodata

National and subnational (both first and second administrative level) boundaries were obtained from WHO. In Nigeria, the first administrative level corresponds to states, and the second, to local government areas; in Pakistan, the first administrative level corresponds to provinces, and the second, to districts. For simplicity, we use here the word “district” to refer to districts in Pakistan and local government areas in Nigeria, and the word “state” to refer to provinces in Pakistan and states in Nigeria. The district-level administrative boundaries in Pakistan have changed over time. Here we retain the boundaries of districts prior to 2010 to be able to examine trends over time. Moreover, we consider Karachi (the most populous city in Pakistan) as two administrative units, based on historic polio epidemiology: (i) Gadap and Gulshan Iqbal and (ii) the rest of Karachi. Maps of Nigeria and Pakistan with the names of the states are provided in Figure I in S1 Text.

#### Acute flaccid paralysis data

The global surveillance for poliomyelitis is conducted through surveillance for acute flaccid paralysis (AFP) cases. In each country AFP cases are reported through a network of healthcare providers [19]. For each AFP case, information recorded includes the state and district in which the individual resides; the dates of onset, notification, and stool collection; the age and sex of the individual; and the reported number of OPV doses received (with RI and SIA doses recorded separately for countries in the Eastern Mediterranean Region but not in the African Region). All poliomyelitis cases are confirmed through isolation and sequencing of poliovirus from stool collected from notified AFP cases. Here, we used data from AFP cases with clinical onset between 1 January 2004 and 30 June 2015. Institutional ethics approval for this study was not sought because the databases are free of personally identifiable information.

#### SIA data

A calendar of implemented and planned SIAs is maintained by the Emergency Operations Centres in both Pakistan and Nigeria. The calendars provide the dates of implementation of each SIA at the district level and the vaccine type used (including the different OPV formulations and IPV). We obtained data for the SIAs implemented from 1 January 2004 to 30 June 2015 (Figures IV–VII in S1 Text) and those planned between 1 July 2015 and 31 March 2016 (Figures XXIV and XXV in S1 Text).

### Statistical Analyses

#### Estimates of serotype-2 population immunity in children <36 mo

Vaccine-induced population immunity against poliomyelitis due to serotype-2 poliovirus by district and 6-mo period from 1 January 2004 to 30 June 2015 was estimated for Nigeria and Pakistan based on the reported number of OPV doses received by non-polio AFP cases <36 mo of age (40,614 and 24,730 cases in Nigeria and Pakistan, respectively), the history of SIAs, and estimates of vaccine efficacy, using the methods described in [20] (assuming a per-dose efficacy of tOPV against serotype-2 poliomyelitis of 48.5% [95% CI 43.1%–53.1%] [21]). In brief, each child’s vaccination history with tOPV was inferred (given the number of reported vaccine doses received and the number of tOPV SIAs a child was exposed to), and individual protection against serotype-2 poliomyelitis was derived assuming a constant per-dose probability of protection (Section A.1.1 in S1 Text). These estimates do not account for immunity resulting from transmission of OPV among contacts of vaccinated children, maternally derived antibodies, or naturally acquired immunity following exposure to cVDPV2. In Nigeria, receipt of tOPV through RI was inferred from district-level coverage with three doses of diphtheria-tetanus-pertussis (DTP3) vaccine at the age of 12 mo or later estimated from Demographic and Health Surveys (DHS) data (estimates are derived in [22]). In Pakistan, receipt of tOPV through RI was reported separately for all children with AFP. Crude estimates of population immunity per district and 6-mo period were obtained by taking the mean over the individual estimates of protection from all non-polio AFP cases <36 mo old in a given district and 6-mo time period. To account for data sparsity, the crude estimates were then spatially and temporally smoothed using a random-effects spatio-temporal model in a Bayesian framework [23] (further details are given in Section A.1.2 in S1 Text).

#### Projections of serotype-2 population immunity in children <36 mo in April 2016

Forward projections of serotype-2 immunity from July 2015 to April 2016 (the time of the tOPV-bOPV switch) in Nigeria and Pakistan were obtained using a cohort model (Section C in S1 Text). Age-specific estimates of population immunity for children <36 mo old were produced for the baseline period (1 January–30 June 2015). The baseline estimates for districts that received SIAs with IPV between 1 January 2014 and 31 July 2015 were adjusted to account for the impact of IPV on population immunity (a single dose of IPV was assumed to have an efficacy of 60% against serotype-2 poliomyelitis [95% CI 54.6%–70.4%] [24] when administered at ≥14 wk of age).

Briefly, the projections assume that RI coverage remains the same as in the baseline period and that future SIAs occur according to the GPEI plans (Figures XXIV and XXV in S1 Text). SIA coverage from July 2015 to April 2016 and the presence of under-vaccinated groups were both assumed to be the same as those of the period January 2014–June 2015, which were estimated through a statistical framework described in [25] using reported vaccination histories of non-polio AFP cases aged <36 mo old and the expected number of SIA doses received. The modelling framework can test for the presence of heterogeneity in coverage, which is modelled by splitting the population into two groups: a better-vaccinated group and an under-vaccinated group (see [25] and Section B.1 in S1 Text for details).

Projections therefore account for the presence of under-vaccinated communities and include different immunity estimates for the under-vaccinated group and the better-vaccinated group at baseline (Section C.1.2 in S1 Text). Projections with alternative SIA plans or that assumed improvements in access to children in high-risk areas in Pakistan were explored. The projections also include the introduction of one dose of IPV given at 14 wk through RI (Section C.1.3 in S1 Text).

Uncertainty analyses of the immunity projections were performed to assess the robustness of the results to uncertainty in the input parameters (i.e., the baseline estimates of immunity, SIA coverage, and efficacy of tOPV and IPV). For these, we used a Latin hypercube sampling approach [26], which allows efficient exploration of the multidimensional parameter space (Section C.1.6 in S1 Text). Finally, to test to what extent the hypothesis of non-independence between RI coverage and SIA coverage could lead to lower projected immunity, we considered as a sensitivity analysis an extreme scenario wherein individuals in the under-vaccinated group did not receive any doses of tOPV through RI (Section C.1.7 in S1 Text).

All statistical analyses were performed using the R programming language [27].

## Results

### Serotype-2 Population Immunity January 2004 to June 2015 and Incidence of cVDPV2 Poliomyelitis Cases

Coverage of three doses of tOPV delivered through RI was heterogeneous across Nigeria (Figure II in S1 Text) and Pakistan (Figure III in S1 Text), with many areas in both countries having persistently low RI coverage over the study period, January 2004 to June 2015 (Section A.2 in S1 Text). Therefore serotype-2 population immunity in those areas heavily relied on SIAs. Since 2004, the number of tOPV SIAs over time markedly changed in both countries (Figures IV–VII in S1 Text). Overall, estimates of population immunity to serotype-2 poliomyelitis in children <36 mo old in both countries reflect the spatio-temporal trends in the number of tOPV SIAs.

**Fig 1:**
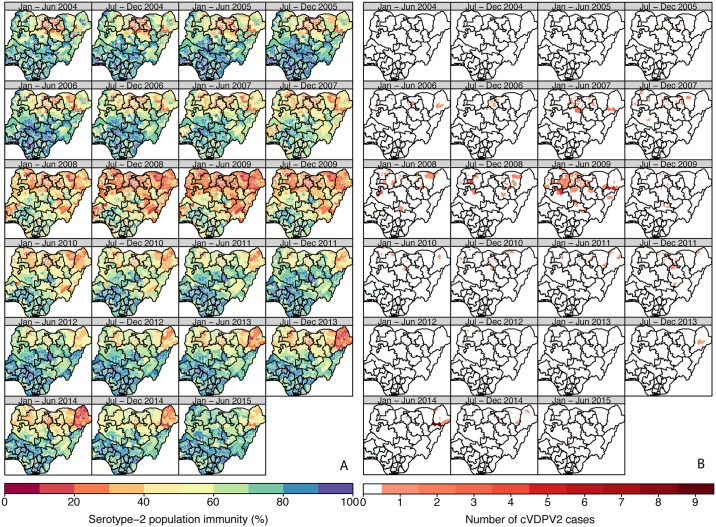
Estimated serotype-2 population immunity and number of cVDPV2 cases in Nigeria, January 2004 to June 2015. (A) Estimated serotype-2 population immunity in children <36 mo of age and (B) number of cVDPV2 cases, for 6-mo periods from January–June 2004 to January–June 2015. The first cVDPV2 cases were reported during the second half of 2005. The publication of this map does not imply the expression of any opinion whatsoever on the part of WHO concerning the legal status of any territory, city, or area or of its authorities, or concerning the delimitation of its frontiers or boundaries.

**Fig 2:**
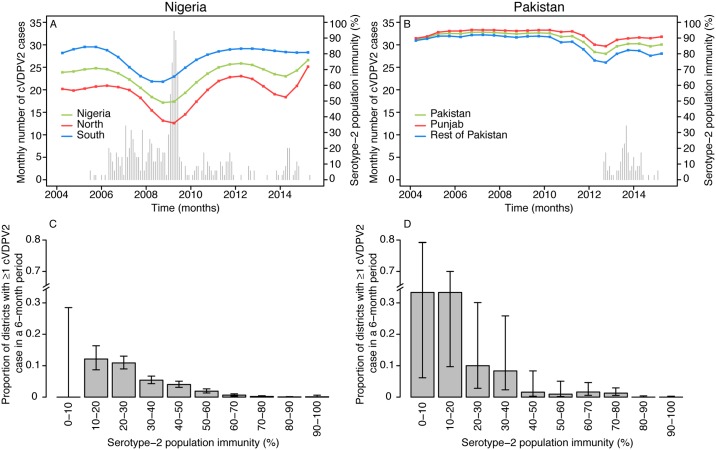
Spatio-temporal correlation between serotype-2 population immunity and the occurrence of cVDPV2 cases. Country estimates of serotype-2 population immunity and monthly number of cases of cVDPV2 in (A) Nigeria and (B) Pakistan. Proportion of districts (with 95% confidence intervals) reporting at least one cVDPV2 case within a 6-mo period among all districts/6-mo periods by intervals of 10% of estimated serotype-2 population immunity for (C) Nigeria and (D) Pakistan.

In Nigeria, population immunity to serotype-2 was estimated to be consistently lower in the northern districts compared to the southern districts (Fig 1A; Figure XXXII in S2 Text; 95% credible intervals [CrIs] in Figure VIII in S1 Text), reaching its lowest level in 2008–2009 (in the second half of 2008, ~75% of the northern districts had estimates of immunity < 50%, compared to ~25% of the southern districts). Immunity then progressively increased nationally with the exception of Borno and Kano (in the first half of 2014, 20/27 districts in Borno and 28/44 in Kano had estimates < 30%). The first cVDPV2 cases in Nigeria were reported in July 2005 in Bauchi and Lagos states (Fig 1B). The incidence of cVDPV2 cases then rapidly increased across the northern states, peaking in 2009, with >150 cases reported that year, coinciding with the general decrease in population immunity (Figs 1 and 2A). From 2010, the incidence of cVDPV2 cases declined, and in 2014, a total of 30 cases were reported, primarily from Borno (14/30) and Kano (10/30), where population immunity was lowest (range 14%–63% in Borno and 16%–58% in Kano). The last cVDPV2 case was reported on 16 May 2015. Nigeria has reported more cVDPV2 cases than any other country, with >440 cases as of 20 October 2015 reported from 193 districts (Fig 1B).

**Fig 3:**
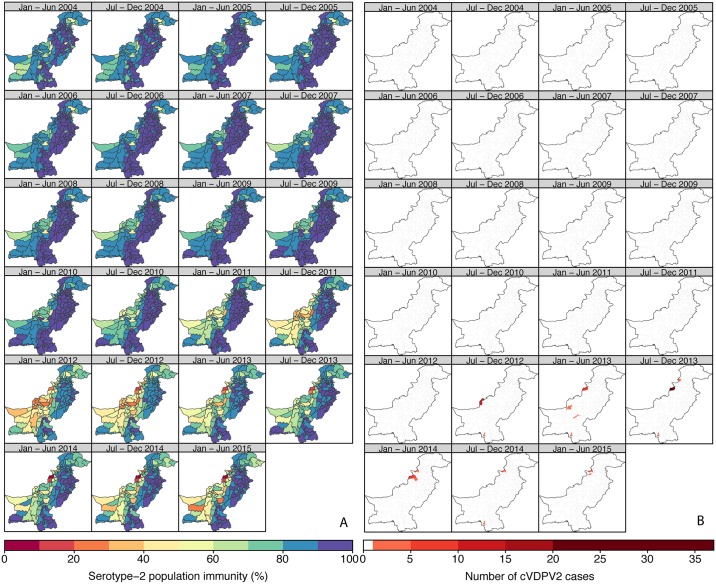
Estimated serotype-2 population immunity and number of cVDPV2 cases in Pakistan, January 2004 to June 2015. (A) Estimated serotype-2 population immunity in children <36 mo of age and (B) number of cVDPV2 cases, for 6-mo periods from January–June 2004 to January–June 2015. The first cVDPV2 case was reported during the second half of 2012. The publication of this map does not imply the expression of any opinion whatsoever on the part of WHO concerning the legal status of any territory, city, or area or of its authorities, or concerning the delimitation of its frontiers or boundaries.

In contrast to Nigeria, population immunity to serotype 2 in Pakistan was nationally very high (>80% in most of the districts) from 2004 to 2010 (Fig 3A; Figure XXXIII in S3 Text; 95% CrIs in Figure IX in S1 Text). Immunity was estimated to be consistently lower in Federally Administered Tribal Areas (FATA), Khyber Pakhtunkhwa (KPK), and Balochistan than in the rest of the country and began to decline nationally from 2011 onwards, reaching its lowest level in 2012 (in the second half of 2012, 31% of the districts in FATA, KPK, and Balochistan had estimates of immunity < 50%, compared to none of the districts in the remaining states). In particular, North and South Waziristan (districts of FATA) had the most dramatic decline, with estimates of immunity < 30%. The first cVDPV2 case in Pakistan was reported in the second half of 2012 in Killa Abdullah, Balochistan, and was followed by 13 additional cases in this district by the end of the year, coinciding with an estimate of immunity < 40% (Fig 3). Most of the subsequent cases have been concentrated in areas of Balochistan, FATA, KPK (bordering districts of FATA), and Karachi, where population immunity has been low (median 54%, interquartile range 25%–72%). In 2013–2014, North Waziristan reported a high of 49 cases, likely due to the consistent low immunity (<20%) (Fig 3B). The last two cases had a date of paralysis onset in February 2015 and occurred in the Khyber and Hangu districts. Pakistan has reported a total number of 87 cVDPV2 cases as of 20 October 2015 (Fig 2B).

SIAs with IPV were implemented in a few high-risk districts in both countries in 2014 and 2015 (Figures X and XI in S1 Text) to increase population immunity and accelerate the interruption of wild poliovirus and cVDPV2 transmission. Estimates of serotype-2 population immunity accounting for both OPV and IPV SIAs (Section A.1.3 in S1 Text) are shown in Figures X and XI in S1 Text.

As expected, historical trends in Nigeria and Pakistan indicate that low serotype-2 population immunity is associated with a higher probability that a district will report a cVDPV2 case (Fig 2). In Nigeria, out of a total of 342 district/6-mo period observations that had at least one cVDPV2 case, 83% had levels of population immunity < 50%, and 97% had levels < 70% (Fig 2C). In Pakistan, only 17 district/6-mo period observations reported at least one cVDPV2 case. Of those, eight (47%) had immunity < 50%, and 12 (71%) had immunity < 70% (Fig 2D). However, in Pakistan most of the cases were focussed in space and time (Fig 3B), with 41% of cases occurring in one district (North Waziristan) in a single 6-mo period (second half of 2013), when immunity was estimated to be 11%.

### SIA Coverage from January 2014 to June 2015

Estimates of SIA coverage were required to project population immunity levels for April 2016 (Section B.2 in S1 Text). In Nigeria, two populations, an under-vaccinated and a better-vaccinated group, were assumed to occur in each state, and approximately 50% of the population of each state was estimated to be in the under-vaccinated group (Figure XXI in S1 Text). Under-vaccinated groups were estimated to have SIA coverage between 58% and 70% in the southern states and between 16% and 62% in the northern states, with Borno having the lowest estimate, of only 16.8% (95% CrI 13.3%–20.3%) (Figure XXI in S1 Text). Better-vaccinated groups had estimated SIA coverage >60% in all states (Figure XXI in S1 Text), with the exception of Borno, where coverage was estimated to be 57.7% (95% CrI 53.8%–61.7%).

In Pakistan, the presence of two coverage groups was tested in each region (Section B.1.2 in S1 Text), and there was evidence for the presence of under-vaccinated groups in the majority of the country, with the exception of Punjab (Figure XXII in S1 Text). Among areas with evidence of under-vaccinated groups, the fraction of the population in that group was heterogeneous, 30%–80%, with the larger proportions corresponding to specific districts of Balochistan, FATA, KPK, and Karachi (Figure XXII in S1 Text). Under-vaccinated groups in Balochistan, northern and central KPK, and Karachi were estimated to have coverage < 40%, whilst coverage in FATA and southern KPK was 10.0% (95% CrI 7.9%–12.2%) (Figure XII in S1 Text). SIA coverage in the better-vaccinated groups (or in the general population, for those areas with no evidence of under-vaccinated groups) was estimated to range between 40% and 80%, with the lowest estimates in FATA and southern KPK (Figure XXII in S1 Text).

### Projections of Serotype-2 Population Immunity in April 2016 at the Time of OPV2 Withdrawal

**Fig 4:**
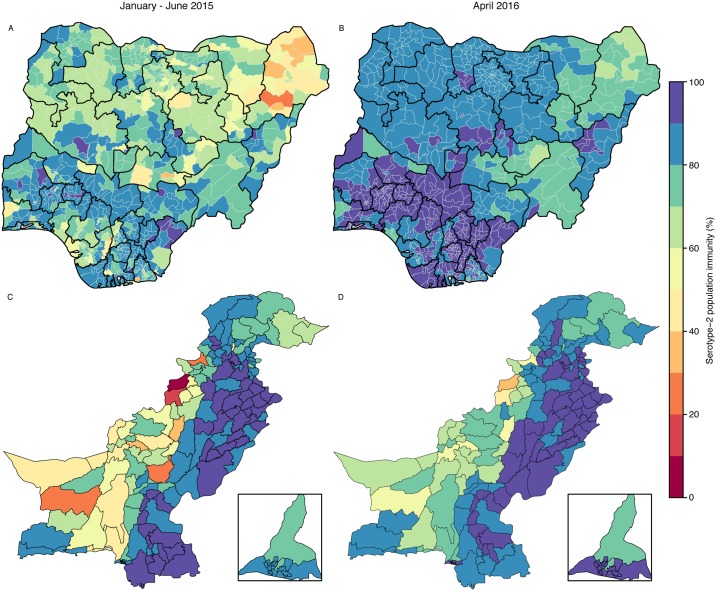
Estimated serotype-2 population immunity in January–June 2015 and projected serotype-2 population immunity in April 2016 in Nigeria and Pakistan. Estimated serotype-2 population immunity in January–June 2015 in (A) Nigeria and (C) Pakistan. Projected serotype-2 population immunity at the moment of OPV2 withdrawal (April 2016) in (B) Nigeria and (D) Pakistan. The inserts of (C) and (D) show the administrative areas of Karachi. The publication of this map does not imply the expression of any opinion whatsoever on the part of WHO concerning the legal status of any territory, city, or area or of its authorities, or concerning the delimitation of its frontiers or boundaries.

Population immunity against serotype 2 in Nigeria in the first half of 2015 increased in most of the northern states compared with 2014. About 75% of districts had estimates > 70%, although 29 districts had estimates < 50%, most of them concentrated within Borno (15/27 of its districts) (Fig 4A). The GPEI had planned two national and five subnational tOPV SIAs in Nigeria between July 2015 and April 2016 (Figure XXIV in S1 Text). Projected levels of immunity for April 2016 improved generally compared to estimates for January–June 2015, and in particular in the northeast states, with all districts in Nigeria having projected estimates > 65%, and around 90% of districts having estimates > 80% (Fig 4B). Despite the general improvements, districts with the lowest projected estimates were mostly in Borno (Fig 4B), where SIA coverage has been estimated to be the lowest. The 95% uncertainty intervals of the projections were narrow, with the absolute range of the interval never exceeding 10% of immunity (Fig. XXVI in S1 Text).

In Pakistan, population immunity in the first half of 2015 was estimated to be >80% in Punjab and Sindh (Fig 4C). However, immunity in Balochistan, FATA, and KPK was estimated to be heterogeneous in the first half of 2015: estimates of immunity in North and South Waziristan and Khyber (three districts within FATA) were <30%. Before April 2016, Pakistan had planned one national and two subnational tOPV SIAs, together with three subnational IPV SIAs (Figure XXV in S1 Text). Based on those plans and assuming vaccination coverage similar to that of January 2014–June 2015, population immunity in April 2016 was predicted to increase nationally (84% of districts with immunity > 70%), although immunity in North and South Waziristan was predicted to be <50% (Fig 4D). The 95% uncertainty intervals of the projections were also narrow for the majority of districts (75% of districts had an absolute range not exceeding 10% of immunity), whilst the remainder of districts (located in Balochistan, Gilgit-Baltistan, and Azad Jammu and Kashmir) had a broader range, not exceeding 25% (Figure XXVII in S1 Text).

Forward projections assuming improvements in vaccine coverage before April 2016 in FATA (North and South Waziristan, Kurram, and Khyber), Peshawar, and Karachi (shown in Figure XXIII in S1 Text) predicted increased immunity in these areas (Figure XXIX in S1 Text), particularly in North and South Waziristan, with absolute increases of 20% and 17%, respectively. We also projected immunity under alternative tOPV SIA plans (Figure XXX in S1 Text). Implementing one additional subnational campaign with tOPV in January 2016 or one additional national campaign in December 2015 was predicted to improve immunity nationwide (84% and 91% of districts with immunity > 70%, respectively); however, estimates in North and South Waziristan would remain <50% (Figure XXX in S1 Text).

## Discussion

In April 2016, OPV2 was globally and synchronously withdrawn from 155 countries within a 2-wk period. This unprecedented global public health intervention is a key milestone of the polio eradication programme for complete eradication of poliomyelitis due to serotype 2. OPV2 withdrawal will lead to cohorts of unimmunised children at risk of developing serotype-2 poliomyelitis if there is subsequent exposure, and, therefore, efforts to interrupt transmission of any cVDPV2 were required before OPV2 withdrawal. In this context, information on the levels of serotype-2 population immunity at the time of the OPV2 withdrawal and during the preceding months was essential to assess such risk, and perhaps adapt the tOPV SIA plans if the levels of population immunity were considered too low. In August 2015, we estimated population immunity in the two highest risk countries, Nigeria and Pakistan, at the subnational level for January–June 2015 and projected immunity to April 2016. Interpretation of immunity levels was provided through analysis of a decade of spatio-temporal trends of estimated population immunity and incidence of reported cVDPV2 poliomyelitis in both of these countries.

In Nigeria the first reported cases of cVDPV2 occurred in 2005, when the number of WPV1 and WPV3 cases were very high [21], and efforts were focussed on interrupting wild-type poliovirus transmission. The introduction of mOPV1 in 2006, mOPV3 in 2007, and bOPV in 2009 into SIAs led to a rise in serotype-1 and serotype-3 population immunity, specifically in the northern districts (Figure XXXI in S1 Text), but also to a substantial drop in serotype-2 immunity across the country that was extended through 2007–2010 and likely favoured extensive spread of cVDPV2. This led to the biggest cVDPV2 outbreak reported (>440 cases), with most of the cases occurring in the north, where population immunity mostly relies on SIAs due to inadequate RI coverage. As the incidence of serotype-1 and serotype-3 cases declined, an increased use of tOPV in SIAs was initiated, resulting in progressive increases in serotype-2 population immunity since 2010. This has probably been responsible for the decrease in cVDPV2 cases since then, with only one cVDPV2 case reported in 2015.

In contrast, the first cVDPV2 case in Pakistan did not occur until 2012, and was preceded by a rapid decrease in serotype-2 population immunity. The temporal pattern of serotype-2 immunity was strikingly different in Pakistan compared to Nigeria, with estimates nationally very high until 2011, but as in Nigeria, the decline in immunity coincided with a reduction in tOPV use in SIAs. Prior to 2010, tOPV was the main vaccine used in SIAs, but from 2010, following a surge of WPV1 cases, bOPV began to replace tOPV in SIAs, leading to a subsequent decline of population immunity, particularly in parts of FATA, KPK, Balochistan, and Karachi. Immunity in Punjab remained relatively high, largely due to high RI and SIA coverage. cVDPV2 cases have since been detected in a few localised areas of the country: high-risk districts of FATA, KPK, Balochistan, and Karachi (Gadap). Certain populations in these areas have been inaccessible to polio vaccinators in recent years [28], which has contributed substantially to the decline in population immunity in those areas. In 2015, two cVDPV2 cases were reported in Pakistan, with dates of onset in February. No cases have been reported since then.

In recent years, smaller outbreaks of cVDPV2 have been reported in other countries, mostly in Afghanistan, Cameroon, Chad, Democratic Republic of the Congo, and South Sudan, but transmission was interrupted in these countries by 2014. We additionally estimated serotype-2 population immunity in these countries from January 2010 to June 2015 (Section A.1.4 in S1 Text; Figures XII–XVII in S1 Text). In the first half of 2015, Afghanistan, north Cameroon, Chad, and Democratic Republic of the Congo were all predicted to have higher estimates of serotype-2 population immunity compared to estimates from 2012–2014, suggesting that the spread of potentially imported or newly emerged VDPV2s would be limited in these settings (Figure XVIII in S1 Text; 95% CrIs in Figure XIX in S1 Text). However, the number of non-polio AFP cases per 6-mo period informing these immunity estimates was much lower for these countries than for Nigeria and Pakistan, meaning that only state estimates were derived. The lower resolution of these estimates may mask the presence of under-immunised populations among districts within the larger administrative areas. Nevertheless, the GPEI conducted multiple SIAs with tOPV in these countries (Afghanistan, Cameroon, Chad, Democratic Republic of Congo, and South Sudan) between July 2015 and April 2016 (Figure XX in S1 Text) to increase serotype-2 population immunity and also conducted focussed IPV SIAs in Afghanistan.

The historical observations from Nigeria and Pakistan linking declines in serotype-2 immunity (following the reduction of tOPV use in SIAs) to the subsequent reporting of cVDPV2s highlight the need to ensure high serotype-2 immunity at the time of OPV2 withdrawal. This is important for both the prevention of new VDPV2 emergences [22] and the interruption of potential silent transmission of cVDPV2s. Data from Nigeria and Pakistan suggest a strong relationship between low levels of immunity and the probability that a district reports ≥1 cVDPV2 case (although a small proportion of districts with relatively high immunity reported ≥1 cVDPV2 case, likely due to heterogeneity in SIA coverage within certain districts). Note that the relationship between low levels of immunity and the probability of a district reporting a cVDPV2 case slightly differs between Nigeria and Pakistan, which is likely due to differences in the number of districts, the population size per district, and other factors that can affect the spatial spread of polioviruses in both countries. Determining the threshold of immunity that interrupts transmission is difficult, but the data from Nigeria suggest that population immunity > 70% dramatically reduces the chances of observing a cVDPV2 case.

The predicted immunity estimates for April 2016 were based on SIA coverage estimated for January 2014–June 2015. In 2015, however, areas of inaccessibility decreased in hard-to-reach areas of Pakistan [28], possibly resulting in improvements in SIA coverage. We projected immunity estimates for Pakistan under an alternative scenario that assumed improved access in certain areas of FATA, Peshawar, and Karachi, whereby immunity estimates were predicted to increase, with all districts having estimates > 50%. Close monitoring of these populations will be essential.

Past experience in Nigeria highlights the cost of prioritising the use of monovalent and bivalent vaccines above tOPV in terms of declines in serotype-2 immunity and subsequent risk of increased cVDPV2 incidence. Pakistan and Afghanistan were in a unique and challenging position, as they had to mitigate the risk of cVDPV2 in preparation for the global OPV2 withdrawal whilst continuing efforts to interrupt WPV1 circulation. Implementation of additional SIAs with tOPV prior to the removal of OPV2 in Pakistan were predicted to improve serotype-2 population immunity; however, due to the modestly lower efficacy of tOPV compared to bOPV against serotype 1 [16], an optimal combination of tOPV and bOPV had to be achieved to ensure sufficiently high serotype-1 immunity in April 2016. Although the balance in the use of the different OPVs is an important consideration, the key factor to achieve interruption of serotype-1 poliovirus while preventing cVDPV2 is ultimately to improve vaccine coverage by reaching inaccessible and persistently missed populations.

There are limitations to our analysis. First, the immunity estimates are based on the reported number of OPV doses received by non-polio AFP cases, and, therefore, we assume that non-polio AFP cases are representative of the entire population. Our method is also contingent upon the correct reporting of the current residence of non-polio AFP cases. For example, if non-polio AFP cases from displaced populations report their district of origin rather than their current district of residence (where they may have been exposed to SIAs), estimates of immunity in the district of origin may be overestimated. Encouragingly though, the immunity estimates show good correlation with the location and timing of reported cases of cVDPV2. Second, the estimates and projections accounting for SIAs with IPV, as well as for the introduction of one dose of IPV into RI, assume the same coverage as estimated for OPV, because the number of IPV doses received by children is not currently recorded during AFP surveillance. Although data from independent monitoring of SIA coverage suggest this assumption may be reasonable, it may not be universally true, particularly given the different modes of administration of the two vaccines and the inability to deliver IPV directly to households. Third, in Nigeria, DTP3 coverage from DHS surveys was used to estimate the RI coverage that informs serotype-2 population immunity. The last DHS survey for Nigeria was carried out in 2013, and we used estimates of RI based on those data from 2013 onwards, but there is some evidence that national rates may have marginally improved since then [29]. Fourth, our projections assume the same RI coverage in under- and better-vaccinated populations during SIAs. A sensitivity analysis considering an extreme scenario wherein the under-vaccinated group was assumed to receive zero doses of tOPV through RI showed that this assumption slightly biased our projections upwards in areas with high RI coverage, but the projections remained nearly unaffected in areas of low RI coverage (i.e., those of particular concern) (Section C.1.7 and Table III in S1 Text). Finally, we estimated serotype-2 population immunity induced by direct immunisation with OPV and IPV against the development of poliomyelitis (humoral immunity). We did not attempt to separately estimate mucosal immunity against infection and shedding of poliovirus, which may differ because of imperfect and waning mucosal protection after OPV and the limited impact of IPV on mucosal protection in children who have not previously been immunised with OPV [11,30]. We did find that our estimates of population immunity correlated strongly with the incidence of poliomyelitis, which suggests that in the context of widespread immunisation with OPV, humoral immunity is a reasonable proxy for intestinal immunity. However, following OPV2 withdrawal, a reduction in mucosal immunity faster than humoral immunity may allow silent circulation of cVDPVs. In addition, the introduction of IPV into RI will also widen the gap between humoral and mucosal immunity. Environmental surveillance (testing sewage for the presence of poliovirus [31]) will therefore be a critical surveillance tool for monitoring whether there is evidence of silent circulation of poliovirus.

Continued AFP surveillance coupled with environmental surveillance will be critical after global OPV2 withdrawal to detect cVDPV2 outbreaks and respond quickly. Environmental surveillance may help detect the emergence of new VDPV2s and also the possible inadvertent use of OPV2. A mOPV2 stockpile has been prepared in order to respond to serotype-2 polio outbreaks that may occur following the global OPV2 withdrawal [32]. If a new outbreak arises, updated immunity estimates similar to those provided here will be valuable to identify the extent of areas at risk and to inform the appropriate scale of the response.

In October 2015, SAGE endorsed the global OPV2 withdrawal in April 2016 [18]. Ultimately, this is the definitive strategy to eliminate OPV2-related polio. Gains in population immunity were projected at the time of OPV2 withdrawal compared to the first half of 2015 in areas that have been of particular concern in Nigeria and Pakistan. Given the improvements in population immunity and the small number of cVDPV2 cases reported in 2015, the global perspective for OPV2 withdrawal in April 2016 was promising, but vigilant surveillance during this period—particularly in areas where projected estimates are the lowest such as Borno in Nigeria and FATA in Pakistan—is essential to ensure there is no further VDPV2 circulation.

## Supporting Information

S1 Text Supplementary information. (PDF)

S2 Text Crude and estimated serotype-2 population immunity in Nigeria by district and 6-mo period from January–June 2004 to January–June 2015. (PDF)

S3 Text Crude and estimated serotype-2 population immunity in Pakistan by district and 6-mo period from January–June 2004 to January–June 2015. (PDF)

## Abbreviations

AFP: acute flaccid paralysis
bOPV: bivalent oral poliovirus vaccine
CrI: credible interval
cVDPV2: circulating serotype-2 vaccine-derived poliovirus
cVDPV: circulating vaccine-derived poliovirus
DHS: Demographic and Health Surveys
FATA: Federally Administered Tribal Areas
GPEI: Global Polio Eradication Initiative
IPV: inactivated poliovirus vaccine
KPK: Khyber Pakhtunkhwa
mOPV{1,2,3}: serotype-{1,2,3} monovalent oral poliovirus vaccine
OPV: oral poliovirus vaccine
OPV{1,2,3}: serotype-{1,2,3} oral poliovirus vaccine
RI: routine immunisation
SAGE: Strategic Advisory Group of Experts on Immunisation
SIA: supplementary immunisation activity
tOPV: trivalent oral poliovirus vaccine
VAPP: vaccine-associated paralytic poliomyelitis
VDPV: vaccine-derived poliovirus
VDPV2: serotype-2 vaccine-derived poliovirus
WPV{1,2,3}: serotype-{1,2,3} wild poliovirus
