# Supplementary material for: Population Immunity against Serotype-2 Poliomyelitis Leading up to the Global Withdrawal of the Oral Poliovirus Vaccine: Spatio-temporal Modelling of Surveillance Data
Source: PLoS Med. 2016 Oct 4;13(10):e1002140. doi: 10.1371/journal.pmed.1002140 (PMC5049753; doi:10.1371/journal.pmed.1002140)
Supplement: S1 Text — (PDF) [file pmed.1002140.s001.pdf]

S1 Text – Supplementary Information

Population immunity against serotype-2 poliomyelitis leading up to the global withdrawal of the oral poliovirus vaccine: Spatio-temporal modelling of surveillance data

Margarita Pons-Salort, Natalie A Molodecky, Kathleen M O'Reilly, Mufti Zubair Wadood, Rana M Safdar, Andrew Etsano, Rui Miguel Vaz, Hamid Jafari, Nicholas C Grassly and Isobel M Blake

Contents

A Estimating population immunity to serotype 2 poliomyelitis 2

A.1 Supplementary Materials and Methods . . . 2

A.1.1 Crude estimates of population immunity by district and 6-month period . . . 2

A.1.2 Spatio-temporal models of population immunity . . . 2

A.1.3 Accounting for SIAs with IPV . . . 4

A.1.4 Estimates of population immunity for other countries . . . 5

A.1.5 Estimating population immunity to serotypes 1 and 3 . . . 5

A.2 Supplementary Results for Nigeria and Pakistan . . . 6

A.3 Supplementary Results for other countries . . . 6

B Estimation of SIA coverage 7

B.1 Supplementary Methods . . . 7

B.1.1 Description of the methods to estimate SIA coverage . . . 7

B.1.2 Data analysis in Pakistan . . . 8

B.1.3 Data analysis in Nigeria . . . 8

B.2 Supplementary Results . . . 8

B.2.1 Nigeria . . . 8

B.2.2 Pakistan . . . 8

C Serotype-2 population immunity projections 9

C.1 Supplementary Methods . . . 9

C.1.1 General overview . . . 9

C.1.2 Age-specific estimates for the baseline period (Jan-Jun 2015) . . . 9

C.1.3 Forward projections for routine immunisation . . . 10

C.1.4 Forward projections for SIAs . . . 11

C.1.5 Forward projections for routine immunisation and SIA . . . 11

C.1.6 Analysis of uncertainty in the projections . . . 11

C.1.7 Testing the assumption of non-independence between RI and SIA . . . 12

C.1.8 Forward projections for serotype-1 population immunity . . . 12

C.2 Supplementary Results . . . 12

D Supplementary Figures and Tables 14

A Estimating population immunity to serotype 2 poliomyelitis

A.1 Supplementary Materials and Methods

A.1.1 Crude estimates of population immunity by district and 6-month period

We estimated population immunity against serotype-2 poliomyelitis for each district of Nigeria and Pakistan for children <36 months old for 6-month periods from Jan-Jun 2004 to Jan-Jun 2015 based on the number of doses of OPV reported by non-polio AFP cases, estimates of vaccine efficacy and the SIA calendar. We define the "crude" estimate of population immunity for a given district and 6-month period as the mean of the probability of protection for each child with non-polio AFP weighted to ensure equal representation of each single year age group (since the age distribution of children with AFP differs from the underlying population distribution, which is approximately uniform).

The probability that an individual child is protected against serotype-2 is obtained using the number of tOPV doses received, x_{tr}, and a per-dose efficacy against serotype 2 previously estimated at $e_{2,tr} = 0.485$ [1], proceeding as in previously published methods [2, 1]. The method assumes an all-or-nothing protective response to vaccination where the per-dose probability of inducing an immune response is independent of the number of previous doses. The probability that a child is protected is thus given by $1 - (1 - e_{2,tr})^{x_{tr}}$. These estimates do not account for immunity resulting from transmission of OPV among contacts of vaccinated children, maternally-derived antibodies or naturally-acquired immunity following exposure to cVDPV2.

The reported number of OPV doses received by a non-polio AFP case includes those received through routine immunisation (RI) and supplementary immunisation activities (SIAs). Only tOPV is used in RI (up until OPV2 withdrawal) whereas SIAs use different formulations of OPV (tOPV, mOPV1, mOPV3, bOPV) at different times depending on the setting, but the reported number of doses do not specify the vaccine type. To obtain the number of tOPV doses received through SIAs, the number of reported OPV doses received through SIAs was multiplied by the proportion of SIAs with tOPV that the child was exposed to based on the SIA calendar. Therefore, the number of tOPV doses that an individual has received, x_{tr}, corresponds to the sum of tOPV doses from RI and tOPV doses from SIAs.

In Pakistan, the number of tOPV doses received through RI and the number received through SIA are reported separately. However, in Nigeria only the total number of doses (RI+SIA) is reported. Therefore, for Nigeria we used district-specific coverage estimates for RI from [3] (Figure II). For each non-polio AFP case, we generated 1000 possible vaccination histories by defining a binary variable indicating whether the child was immunised through routine services, drawn from a Bernoulli distribution with probability of success given by the routine immunisation coverage in that district 6-month period. For each random draw, if a child was assumed to participate in routine immunisation, three of the total number of doses reported (or the maximum of doses reported, if <3 doses were reported) were assumed to be tOPV –corresponding to the routine immunisation doses– and the remainder were assumed to be received through SIAs. We therefore generated 1000 estimates of the probability of serotype-2 immunity per child, which were used to obtain 1000 estimates of immunity for each district 6-month period by averaging across all children and age group. The final crude estimates were taken as the median of those 1000 estimates.

The number of non-polio AFP cases used for the serotype-2 population immunity estimates were 40 614 for Nigeria, and 24 730 for Pakistan.

A.1.2 Spatio-temporal models of population immunity

We then fitted random-effects spatio-temporal models to the crude estimates to spatially and temporally smooth our estimates. This was important, because the number of non-polio AFP cases by district 6-month period is relatively small, sometimes zero, leading to district 6-month period with

no crude estimate or with crude estimates unusually low or high, extremely affected by the presence of outliers.

Let p_{ijt} be the crude estimate of population immunity in a district i (in a state j) at a 6-month period t, and n_{ijt} the number of non-polio AFP cases used to estimate p_{ijt}. We defined the following random-effects model for p_{ijt}:

$$p_{ijt} \sim \text{Normal}\left(\eta_{ijt}, \frac{\sigma^2}{n_{ijt}}\right)$$
$$\text{logit}(\eta_{ijt}) = \alpha + \gamma_{it} + \delta_{jt} + \omega_t + \mu_i + \nu_i \quad (1)$$

where α is the intercept, γ_{it} and δ_{jt} are two space-time interactions at two different spatial levels (the district, γ, and the state, δ), ω_t is a temporal random effect that accounts for a general time trend, and μ_i and ν_i are respectively the spatial-unstructured and the spatial-structured random effects of the Besag, York and Mollié (BYM) model [4]. With this formulation, η_{ijt} will therefore be the smoothed estimate of population immunity for the district i (in the state j) at a 6-month period t.

Having the state-level effect is important, because most of the SIAs are organised at the state level. The district level accounts for differences in vaccine coverage within states. The interactions with time are necessary, because the number of tOPV SIA in each district 6-month period changed over the study period (Figure IV and Figure V) and because changes in coverage are also likely to have occurred. We assume that both interactions have a structured temporal effect and an unstructured spatial effect, and we assume an autoregressive time structure of second order. That is, we assume a second-order random walk across time for each area (district or state) independently of all other areas. The time unit is 6 months. If T denotes the total number of time periods, I the total number of districts and J the total number of states, the densities of the two space-time random effects can be written as

$$\pi(\gamma|\sigma_\gamma^2) \propto \exp\left(-\frac{1}{2\sigma_\gamma^2}\sum_{t=3}^{T}\sum_{i=1}^{I}(\gamma_{i,t} - 2\gamma_{i,t-1} + \gamma_{i,t-2})^2\right)$$

and

$$\pi(\delta|\sigma_\delta^2) \propto \exp\left(-\frac{1}{2\sigma_\delta^2}\sum_{t=3}^{T}\sum_{j=1}^{J}(\delta_{j,t} - 2\delta_{j,t-1} + \delta_{j,t-2})^2\right)$$

respectively [5]. Finally, the general time trend ω_t is modelled as a second-order random walk

$$\omega_t - 2\omega_{t+1} + \omega_{t+2} \sim \text{Normal}(0, \sigma_\omega^2)$$

and the spatial effects are defined following the BYM model as

$$\mu_i \sim \text{Normal}(0, \sigma_\mu^2)$$

$$\nu_i \sim \text{Normal}\left(\frac{\sum_{k\neq i}\nu_k}{G_i}, \frac{\sigma_\nu^2}{G_i}\right)$$

where G_i is the number of neighbours of district i.

Note that the Normal approximation is used to model population immunity because it is unknown if non-polio AFP cases are immune, and instead only the probability that each case is immune can be derived based on their reported vaccination history and estimated vaccine efficacy. This allows uncertainty in the crude estimates to be scaled to the number of non-polio AFP cases informing those estimates. A similar approach has previously been used [6]. A caveat to this approximation is that this does not hold for very low numbers of non-polio AFP cases and in these instances the model may slightly overestimate low values of true immunity and underestimate high values of true immunity.

The models were fitted to the data using the Integrated Nested Laplace Approximation (INLA) approach [7] implemented in the R-INLA package [8]. The INLA algorithm, proposed by Rue et al. (2009), is a computationally efficient method for Bayesian inference with latent Gaussian models [7].

This approach provides accurate approximations of the posterior distributions in lower computational time than Markov Chain Monte Carlo methods, for example implemented through BUGS or STAN.

Note that we were not able to fit this model to the data from Nigeria, possibly because of important differences over time between northern and southern areas of the country, or due to correlations between different components of the model that made some parameters unidentifiable. We therefore used a simplified version of the model defined in (1) that included the intercept and the two space-time interactions at the district and state levels:

$$\text{logit}(\eta_{ijt}) = \alpha + \gamma_{it} + \delta_{jt}.$$

A.1.3 Accounting for SIAs with IPV

From 2014 onwards, a few districts in Nigeria and Pakistan have performed SIA campaigns with IPV (Figure X and Figure XI). The estimates of serotype-2 population immunity obtained with the methods described above and presented in Figure 1 and Figure 2 of the main text do not account for those campaigns. Unfortunately, it is not possible to accurately estimate population immunity accounting for SIA with IPV, because AFP cases do not report if they have received IPV doses. Furthermore, the relationship between IPV dose administration and immune response is likely to be different to the relationship observed for OPV (all-or-nothing response, independence of protection between doses). However we use an approximation to adjust the 6-month average population immunity estimates obtained above to account for SIA with IPV in the respective districts that received IPV.

Let η_{ijt} be the population immunity estimate for a given district i (in a state j) and a 6-month period t obtained as described above (i.e. accounting for tOPV doses only). If one or more SIA with IPV have taken place during the current or past 6-month periods, this estimate might be a bit higher, and will depend on the fraction of the population under consideration that has been exposed to IPV campaigns and still remains in the cohort of <36 months old. If we assume that SIA with IPV target all children ≥14 weeks and up to at least 36 months old, for a given SIA with IPV that occurred a number u of 6-month periods ago, the current fraction of the population under consideration (<36 months old) that was exposed to that campaign is given by

$$\rho(u) = \begin{cases} \frac{36-3}{36} - u\frac{6}{36} & u = 0, ..., 5 \\ 0 & \text{otherwise.} \end{cases}$$

In other words, $\rho(u)$ is the proportion of the current population of <36 months old that was above 3 months of age u 6-month periods ago. We further assume the coverage of IPV in SIA is the same as that of OPV. The estimate of population immunity accounting for SIA with IPV during the current ($u = 0$) and previous ($u = 1, .., 5$) 6-month periods is finally given by

$$p_{ijt}^{(+I)} = 1 - (1 - \eta_{ijt}) \prod_{u=0}^{5} (1 - \rho(u) c e_{2,I})^{h(u)}$$

where $h(u)$ is the number of SIA with IPV that took place in that district u 6-month periods before the current one, and c is the overall SIA coverage. The parameter $e_{2,I}$ is the IPV efficacy against serotype 2 for a first dose of IPV, set at $e_{2,I} = 0.6$ based on data from [9]. Note that the efficacy for a second dose of IPV is likely to be higher [9], but we did not account for this difference here. This assumption does not significantly affect the results, as only two districts in Nigeria and five in Pakistan have had more than one IPV campaign, and the maximum number of IPV campaigns per district in each 6-month period before July 2015 has been 2 (Figure X and Figure XI). In Pakistan, IPV SIAs targeted children <24 months of age (instead of <36), and therefore, we adapted $\rho(u)$ accordingly. Finally, when accounting for IPV SIAs in Pakistan, we multiplied the coverage by the fraction targeted (shown in Figure XI).

This adjustment to account for SIA with IPV in population immunity estimates is approximate as it is applied to the average 6-month estimates and assumes the same coverage as OPV.

A.1.4 Estimates of population immunity for other countries

We also estimated serotype-2 population immunity in other countries that have experienced recent outbreaks of VDPV2: Afghanistan, Chad, Cameroon, Democratic Republic of Congo (DRC), Niger and South Sudan. In those countries, serotype-2 population immunity was estimated between Jan-Jun 2010 and Jan-Jun 2015 as described above, with the exceptions detailed below.

In Afghanistan the number of tOPV doses received through RI was reported separately from the number of OPV doses received through SIAs. For the remaining countries only the total number of doses (RI + SIAs) was reported by each non-polio AFP case and therefore we had to derive the number of doses received through each delivery mechanism. To do this we obtained estimates of coverage of receiving at least one (d_1), two (d_2) and three doses (d_3) of tOPV received through RI at the state level from UNICEF's Multiple Indicator Cluster Survey (MICS) performed in 2010 for Chad, DRC, and South Sudan [10]. Then, for each child reporting receiving ≥1 OPV doses a Bernoulli trial was performed to determine if they received ≥1 RI dose, with a probability determined by d_{1j}, where j indicates the province of the non-polio AFP case. Conditioning on the children who then receive ≥1 RI dose, a Bernoulli trial was performed to evaluate which children received ≥2 doses, with probability d_{2j}/d_{1j}. Finally, conditioning on children who received two doses, a Bernoulli trial was performed to evaluate which children received 3 doses, with probability d_{3j}/d_{2j}. Immunity estimates were then generated for each non-polio AFP case. This was repeated 1000 times and the median immunity estimate per non-polio AFP case was taken before calculating the mean crude population immunity estimate across the district 6-month period.

Subnational estimates of RI coverage were not available for Cameroon or Niger from the MICS survey at the time writing and therefore the immunity estimates presented in this work assume all reported OPV doses were received through SIAs. This is likely to result in an underestimation of population immunity in settings when mOPV or bOPV was primarily used in SIAs.

The number of non-polio AFP cases reported at the district level was relatively low in Afghanistan, Chad, Cameroon, Democratic Republic of Congo (DRC), Niger and South Sudan and therefore we only smoothed our estimates at the state level, i.e.:

$$\mathrm{logit}(\eta_{ijt}) = \alpha + \delta_{jt}.$$

The number of non-polio AFP cases used for the estimates of serotype-2 population immunity were as follows: Afghanistan, 4 876; Chad, 878; Cameroon, 802; DRC, 4 222; Niger, 942; and South Sudan, 640.

A.1.5 Estimating population immunity to serotypes 1 and 3

We also estimated population immunity to serotypes 1 and 3 in Nigeria and serotype 1 in Pakistan using the methods described above and accounting for the different OPV vaccines and their respective efficacies against each serotype. The individual estimates of protection against poliomyelitis due to serotypes 1 and 3 are respectively given by

$$1 - (1 - e_{1,tr})^{x_{tr}}(1 - e_{1,b})^{x_b}(1 - e_{1,m1})^{x_{m1}}$$

and

$$1 - (1 - e_{3,tr})^{x_{tr}}(1 - e_{3,b})^{x_b}(1 - e_{3,m3})^{x_{m3}}$$

where $e_{1,tr}$, $e_{1,b}$ and $e_{1,m1}$ (resp. $e_{3,tr}$, $e_{3,b}$, and $e_{3,m3}$) are the estimates of efficacy against serotype 1 (resp. 3) per-dose of tOPV, bOPV and mOPV1 (resp. mOPV3); and x_{tr}, x_b, x_{m1} and x_{m3} are the estimated number of doses of tOPV, bOPV, mOPV1 and mOPV3 the individual has been exposed to, based on the number of reported doses and the SIA calendar, accounting for the estimates of routine coverage as explained above for serotype 2.

We used the following estimates of efficacy for the two countries. For Nigeria, $e_{1,tr} = 0.194$, $e_{1,b} = 0.295$, $e_{1,m1} = 0.321$, $e_{3,tr} = 0.18$, $e_{3,b} = 0.238$ and $e_{3,m3} = 0.432$, based on data from Nigeria [1]. For Pakistan: $e_{1,tr} = 0.125$, $e_{1,b} = 0.234$ and $e_{1,m1} = 0.345$, based on data from [11].

A.2 Supplementary Results for Nigeria and Pakistan

Coverage of three doses of tOPV delivered through RI was heterogeneous across both Nigeria (Figure II) and Pakistan (Figure III). In Nigeria estimated RI coverage from DHS data (based on data for 3 doses of Diphtheria-Tetanus-Pertussis, DTP3) was lower in the North compared to the South but generally increased nationally from 2003 (median 28%, interquartile range [IQR] 13–46%) to 2013 (median 52%, IQR 23–74%). In Pakistan, RI coverage (based on the % of children with non-polio AFP receiving 3 RI doses) in 2004 was greatest in Punjab (median 67%, IQR (60–79%)) and lowest in the Federally Administered Tribal Areas (FATA), Khyber Pakhtunkhwa (KPK) and Balochistan (median 49%, IQR (28–69%). This pattern was consistent, albeit with more pronounced heterogeneity over time, with RI coverage estimates of 89% (81–92%) in Punjab and 35% (16–68%) in FATA, KPK and Balochistan in the first half of 2015.

The number of tOPV campaigns in Nigeria (Figure IV) and Pakistan (Figure V) has varied considerably over time and within both countries. In Nigeria the largest number of tOPV campaigns nationally occurred in 2005 (with all the districts having between 4 and 7 SIAs with tOPV), followed by a progressive decrease, with no campaigns in 2008 as a result of increasing use of monovalent and subsequently bivalent OPV (Figure IV). This was subsequently followed by a slight increase, with all districts implementing at least one tOPV SIA per year since 2009, except districts in Kano and Borno that had no campaigns in 2013 (Figure IV). In Pakistan the number of tOPV campaigns was greatest in the first half of 2005 and remained relatively high until 2009, with all districts except one implementing 4–8 campaigns per year during that period (Figure V). From 2009, only 1–2 SIAs were performed per year, apart from a few additional focused sub-national campaigns targeting high-risk districts, as a result of increasing use of bOPV (Figure V).

Crude estimates of serotype-2 population immunity in both countries accounting for the use of tOPV only were obtained for the same period (Jan-Jun 2004 to Jan-Jun 2015) and spatio-temporal models were then fitted to the crude estimates. The intercept, α, the precision for the Gaussian observations and for the two space-time interactions, as well as for the general time trend and for the BYM spatial components of the model ($1/\sigma^2$, $1/\sigma^2_\gamma$, $1/\sigma^2_\delta$, $1/\sigma^2_\omega$, $1/\sigma^2_\mu$, and $1/\sigma^2_\nu$) were estimated (Table II). The smoothed estimates of immunity are presented in Figures 1 and 2 of the main text for Nigeria and Pakistan, respectively. A comparison between the crude and smoothed estimates are given in Figures S32 and S33.

SIAs with IPV have taken place in a few districts of Nigeria and Pakistan since 2014 (Figure X and Figure XI). The estimates of serotype-2 population immunity accounting for those campaigns are shown in Figure X and Figure XI. Note that this is an approximation, and the results must be interpreted as an upper bound of the levels of serotype-2 population immunity.

Smoothed estimates of population immunity to serotypes 1 and 3 in Nigeria from Jan-Jun 2004 to Jan-Jun 2015 are shown in Figure XXXI.

A.3 Supplementary Results for other countries

We also estimated serotype-2 population immunity from 2010 to the first half of 2015 in other high-risk countries that had reported cVDPV2 cases in 2013–2015 (Afghanistan (Figure XII), Cameroon (Figure XIII), Chad (Figure XIV), DRC (Figure XV), Niger (Figure XVI), and South Sudan (Figure XVII)) to investigate whether immunity had improved by the first half of 2015 (Figure XVIII). We proceeded as for Nigeria and Pakistan, but using crude estimates at the state level (the numbers of non-polio AFP were too low to obtain estimates at the district level). The parameter estimates of the spatio-temporal models used to smooth the crude estimates are given in Table II.

In Afghanistan immunity was estimated to be >80% in the majority of the country in Jan-Jun 2015 with the exception of Helmand, Kandahar, Zabul and Urozgan where immunity was estimated to be between 60% and 80% (although immunity had significantly improved in Helmand and Kandahar since 2011) (Figure XII). In Cameroon immunity was estimated to have sharply declined nationally in 2012 with province estimates ranging from <10% to <50% (Figure XIII). The Extreme Nord province,

which borders Northern Nigeria and Chad, has experienced a series of focused campaigns since mid-2013 and was estimated to have improved population immunity by Jan-Jun 2015, although <70%. Within Chad, population immunity increased nationally since 2010 with the majority of the country having estimates >70% in 2015 (Figure XIV). An improvement in immunity across DRC was also estimated to have occurred since 2010 onwards with province estimates of 70–80% in Jan-Jun 2015 (Figure XV). Population immunity in Niger was estimated to have experienced a decline from 2010 to 2013 but improved from the second half of 2013 with estimates in the majority of the country >60% in Jan-Jun 2015 (Figure XVI). Finally, population immunity in South Sudan was estimated to be mostly >60% in Jan-Jun 2015 with the exception of Unity state (where the two cVDPV2 cases were reported in 2014), which was estimated to have immunity of only 30–40% (Figure XVII). Overall, in all these countries immunity in the first half of 2015 (Figure XVIII, 95% CrI given in Figure XIX) was estimated to be the highest it has been with the exception of Southern Cameroon (although this region has never reported cVDPV2 cases).

B Estimation of SIA coverage

B.1 Supplementary Methods

B.1.1 Description of the methods to estimate SIA coverage

In order to make accurate projections of population immunity, robust estimates of SIA coverage were required in Nigeria and Pakistan. SIA coverage is defined as the proportion of the target population vaccinated during an SIA. Coverage is an important quantity to know but is difficult to directly estimate. Post-campaign monitoring such as lot quality assurance sampling and market surveys provide some indication of coverage but often over-estimate performance and are not carried out in all locations. Instead, we use data on the reported vaccination histories from non-polio AFP cases <36 months old and compare these values to the expected number of SIA doses received. The error in OPV dose recall is accounted for by estimating the true number of doses as augmented data. The full methods are available in [12] and we provide an overview below.

The statistical method tests for the presence of spatial and temporal heterogeneity by comparing the fit of a series of models to the data [12]. Four models are used to test for heterogeneity in coverage. The simplest model assumes that coverage is constant across all campaigns for all of the target population. The second model (homogeneous-temporal model) allows for coverage to change in time between two defined time periods. The third model (heterogeneous model) accounts for persistently under-vaccinated groups, where the population is divided into two groups that differ in their size and coverage, where the "better-vaccinated" group is consistently more likely to be vaccinated during SIAs than the "under-vaccinated" group. The fourth model is the heterogeneous-temporal model where coverage in the better-vaccinated and under-vaccinated group varies in time. Model parameters were estimated using Markov chain Monte Carlo (MCMC). The MCMC chains were run for 1 million iterations and thinned every 1000 iterations to obtain the posterior distributions of parameter estimates. The most parsimonious yet best fitting model was identified using a rescaled deviance information criteria that accounts for the fit of the model to the data and penalises models with more parameters [12].

The method was previously tested using simulation to establish the sensitivity of model inference and accuracy of model parameters and applied to AFP surveillance data from Pakistan [12].

Vaccination coverage was estimated from Jan 2009-Jun 2015 across two distinct time periods: Jan 2009-Dec 2013 and Jan 2014-Jun 2015. The projections of population immunity in April 2016 were based upon SIA coverage from the latter time period whereas the former time period provided a comparison as to how SIA coverage had changed over time. Estimates of coverage were provided at the state level in Nigeria and at the regional level in Pakistan (regions closely correspond to states but states were in some cases further divided based on the epidemiological situation; see Table I for details on the divisions).

B.1.2 Data analysis in Pakistan

Pakistan was divided into seven regions that were approximately similar to States; Balochistan, FATA & South KPK, North & Central KPK, East Punjab, West Punjab, Karachi and surrounding districts (Karachi+), and the rest of Sindh (Table I). These divisions were selected because they reflected typical subnational vaccination activities and the numbers of non-polio AFP cases available for analysis in each region from Jan 2009-Jun 2015 provided sufficient power to estimate evidence of heterogeneity in coverage. Coverage estimates were not carried out on non-polio AFP cases from Azad Jammu and Kashmir or Gilgit-Baltistan as the number of observations were small (<20 per year). The statistical models were applied to the seven regions separately, resulting in estimates of model fit and coverage estimates for each. Where under-vaccinated populations were identified, the regions were divided into district groupings to estimate the size of the under-vaccinated populations within these smaller divisions (Table I).

B.1.3 Data analysis in Nigeria

Non-polio AFP data from Jan 2009-Jun 2015 for each state in Nigeria were analysed separately, resulting in estimates of SIA coverage for each state. In Nigeria only the combined dose history of RI and SIAs were available, and so some modifications of the methodology were required. For each child we estimated the probability of receiving three RI doses by assigning a prior distribution of district-level RI coverage, based upon estimates from DHS data in [3]. Therefore, SIA coverage in Nigeria was estimated from the combined information regarding the total dose history from SIAs, the SIA calendar and the district estimates of RI from DHS data. When the methods were adapted to data from Nigeria, preliminary analyses suggested the homogeneous coverage model was the most parsimonious model across the majority of states, however, the heterogeneous model provided a better fit. Therefore, the results obtained with the heterogeneous-temporal coverage model were used for the projections. Note that if coverage was truly broadly homogeneous within a population, the estimated coverage for the better-vaccinated and the under-vaccinated groups would be similar in value and the projections would be similar to if a homogeneous model had been used. Due to the low number of observations at the district level, further analysis of the size of the under-vaccinated populations by district was not carried out in Nigeria.

B.2 Supplementary Results

B.2.1 Nigeria

SIA coverage within Nigeria between Jan 2009-Jun 2015 varied considerably across the country, but largely an improvement in coverage was observed over time (Figure XXI). In all states the size of the under-vaccinated group was approximately 50%. Coverage prior to Jan 2014 was very variable, whereby coverage in the better-vaccinated group was sometimes estimated to be <50% (in the Northern states of Borno, Kano, Yobe and Zamfara and the Southern states of Abia, Anambra and Rivers), and coverage in the under-vaccinated group was typically <20%. In 2014-2015 vaccination coverage was estimated to have improved substantially in most states, whereby coverage in the better-vaccinated group was >60% across Nigeria, and coverage in the under-vaccinated group had much spatial variation (varying from 16-70%, where Bauchi, Kano, Kaduna, Taraba and Yobe were estimated to have coverage that was approximately 35% and Borno state was estimated to have the lowest coverage in the under-vaccinated group of 16.8% (95% credible intervals (CrI) 13.3-20.3%), indicating little change when compared to 2009-2012 estimates).

B.2.2 Pakistan

Estimates of SIA coverage in Pakistan from Jan 2009-Jun 2015 varied considerably across regions and by time period (Figure XXII). The heterogeneous-temporal model provided the most parsimonious

yet best fit to the data in FATA & South KPK, Balochistan, North & Central KPK, Sindh and Karachi+, indicating the presence of under-vaccinated populations in these areas and a change in coverage in Jan 2014-Jun 2015. In east and west Punjab the homogeneous-temporal model provided the most parsimonious yet best fit to the data indicating there was no evidence for under-vaccinated populations. There was evidence for improvements in SIA coverage in the better vaccinated group over the two time periods in North & Central KPK [estimated coverage in this group 2009-2013 was 65.3% (95% CrI 63.3-67.6%), and in 2014-2015 coverage was 77.4% (95% CrI 73.0-81.3%)], Karachi+ [estimated coverage 2009-2013 was 52.2% (95% CrI 49.0-55.6%) and in 2014-2015 was 66.5% (95% CrI 58.8-74.2%]. Coverage was estimated to be low in the under-vaccinated groups in Balochistan (coverage in 2014-2015 was 24.6% (95% CrI 19.8-29.7%) and FATA & South KPK (coverage in 2014-2015 was 10.0% (95% CrI 7.9-12.2%). Within Balochistan, FATA & South KPK, North & Central KPK, Sindh and Karachi+, there was considerable variation in the size of the under-vaccinated populations. Over 60% of the population was estimated to be under-vaccinated within the district groupings of Quetta, Khyber, Wazir and Gadap Town in Karachi (Figure XXII).

C Serotype-2 population immunity projections

C.1 Supplementary Methods

C.1.1 General overview

We projected the levels of serotype-2 population immunity in each district of Nigeria and Pakistan from Jan-Jun 2015 (baseline period) to April 2016 (date for OPV2 withdrawal) using a cohort model of children 0–2 years old, where individuals are divided into 36 age classes of one month ($a = 0, 1, ..., 35$). We therefore used a time-step of one month for the forward projections. At each month, children from the age class a ($a = 0, 1, ..., 34$) move to the age class $a + 1$, those in the age class $a = 35$, leave the cohort, and newborns enter the age class $a = 0$. We further assumed that all age classes have the same size, which is a reasonable hypothesis for the young age group we consider and the time period we project over.

Forward projections of immunity account for: 1) the protection acquired through RI, including the introduction of one dose of IPV into RI programs, and 2) the protection acquired through the SIA with tOPV and IPV planned from July 2015 to March 2016 (Figure XXIV and Figure XXV). The population reached by both systems of vaccination is assumed independent. That is, the probability that a child receives the doses of RI is considered independent of the probability of receiving the SIA doses. We thus can write the population immunity in a given district at month t after June 2015 (last month of the baseline period) as

$$p^{(+I)}(t) = 1 - s^{SIA(+I)}(t)s^{RI(+I)}(t) \qquad t = 1, ..., 9$$

where $s^{SIA(+I)}(t)$ and $s^{RI(+I)}(t)$ are the estimates of susceptibility considering only the doses administered through routine or supplementary vaccinations respectively and the extension $(+I)$ in the superscript indicates that those estimates account for SIA with IPV and the introduction of one dose of IPV into RI programmes. Note that the assumption of independence allows us to make the projections of susceptibility for RI and SIA separately, which simplifies the problem.

C.1.2 Age-specific estimates for the baseline period (Jan-Jun 2015)

To obtain the age-specific baseline estimates of susceptibility for RI and SIA respectively accounting for tOPV doses only ($s_a^{RI}(0)$ and $s_a^{SIA}(0)$, for $a = 0, ..., 35$), we performed the same analyses as in Section A.1.2, but dividing the non-polio AFP cases into 12 age-classes of 3 months (0–2, 3–5,..., 33–35), and dividing the number of OPV doses reported by each individual into routine or SIA doses. We added a component to the spatio-temporal model presented in equation (1) to account for a general age trend that was modelled as a second-order random walk.

We then used a cubic spline to estimate the levels of susceptibility by one-month age classes, assuming that the values obtained through fitting the age-structured spatio-temporal models to the crude estimates corresponded to the middle point values of the age intervals used (i.e. 1.5, 4.5,..., 34.5 months). We further assumed that individuals entering the cohort model do not have any protection from SIAs (i.e., $s_0^{SIA}(t) = 1$) nor RI (i.e., $s_0^{RI}(t) = 1$).

Forward projections for SIA account for the presence of under-vaccinated groups within states. For those districts with evidence of an under-vaccinated group, we split the population into two groups: the better-vaccinated and the under-vaccinated, with the proportion in each group defined by the results in Section B. To estimate the susceptibility from SIA in each group at baseline, and therefore start the projections with different susceptibility estimates for the under-vaccinated and the better-vaccinated groups, we did the following approximation. Given the estimated baseline proportion of susceptibles in a given age class a, $s_a^{SIA}(0)$, the estimated size of the under-vaccinated group z_u, and the campaign coverage c_b and c_u in the better- and the under-vaccinated groups respectively, we solved the following equation for $m \in \mathbb{R}^+$:

$$\min[(s_a^{SIA}(0) - ((1 - z_u)(1 - c_b e_{2,tr})^m + z_u(1 - c_u e_{2,tr})^m))^2]. \tag{2}$$

Note that z_u, c_b and c_u are assumed the same across age classes, and are estimated at the state-level for Nigeria and at the region-level for Pakistan (see Section B). In Pakistan, SIA coverage was not estimated for Gilgit-Baltistan and Azad Jammu and Kashmir. For these areas, we used the weighted mean of the better- and under-vaccinated populations of Sindh, because coverage in these regions are likely to be high as demonstrated by the absence of cases. If we denote by $\hat{m}$ the solution of equation (2), the proportion of children susceptible at baseline in the better- and the under-vaccinated groups are then given by

$$s_{a,b}^{SIA}(0) = (1 - c_b e_{2,tr})^{\hat{m}} \qquad \text{and} \qquad s_{a,u}^{SIA}(0) = (1 - c_u e_{2,tr})^{\hat{m}}$$

respectively, where $e_{2,tr}$ is the per-dose of tOPV efficacy against serotype 2 and is set at $e_{2,tr} = 0.485$, based on previously published estimates [1].

We also account for SIAs with IPV until June 2015 (included) in the baseline estimates. Districts that did not have any SIA with IPV are not affected. For those districts that had at least one SIA with IPV, the age-specific baseline estimates were updated as follows. For any age class a, with $a \geq 3$ in June 2015 (note that IPV is administered to children ≥14 weeks, and so age classes under 3 months are not affected), let q_a be the number of SIA with IPV that occurred since the children in that age class were 3 months of age (i.e. during the $(a - 3)$ months up to and including June 2015; e.g. for the age class of 6 months in June 2015, we will take into account the number of SIA with IPV that occurred during March to June 2015 inclusive). The approximation is then given by

$$s_{a,b}^{SIA(+I)}(0) = s_{a,b}^{SIA}(0)(1 - c_b e_{2,I})^{q_a} \qquad a = 3, ..., 35$$

$$s_{a,u}^{SIA(+I)}(0) = s_{a,u}^{SIA}(0)(1 - c_u e_{2,I})^{q_a} \qquad a = 3, ..., 35$$

where c_b and c_u are the estimates of SIA coverage in the better- and under-vaccinated groups respectively and $e_{2,I}$ is the per-dose efficacy of IPV against serotype 2, which is set at $e_{2,I} = 0.6$ [9].

C.1.3 Forward projections for routine immunisation

We assume RI coverage during the projections remains the same as in the baseline period. Therefore, the susceptibility by age class for RI during the projections is the same as in the baseline period (Jan-Jun 2015), except that we also account for the introduction of one dose of IPV into routine programs at 14 weeks (i.e. 3 months). The date of introduction of such differs among states. In Nigeria, the introduction of one dose of IPV was assumed to start in February 2015 in Borno, Yobe, Bauchi and Jigawa; in June 2015 in Rivers and Plateau; and in March 2015 in the other states. In Pakistan, it was assumed to start in July 2015 in Punjab and nationwide in August 2015.

Forward projections of susceptibility for RI at month t (in $1, ..., 9$) accounting for the introduction of one dose of IPV are as follows. Let T_{IPV}^{RI} be the month of introduction of one dose of IPV with respect to June 2015 (e.g. if IPV was introduced in February 2015, $T_{IPV}^{RI} = -4$). If $t < T_{IPV}^{RI}$ (i.e. the introduction of one dose of IPV occurs after t), then the projections at t are not affected and thus $s_a^{RI}(t) = s_a^{RI}(0)$ for all age classes, $a = 0, ..., 35$. If $t \geq T_{IPV}^{RI}$, we denote $A_t = \min(t - T_{IPV}^{RI}, 35)$ the number of age classes that will have been exposed to IPV at month t, and the age-specific estimate of susceptibility is given by

$$s_a^{RI(+I)}(t) = \begin{cases} s_a^{RI}(0)(1 - re_{2,I}) & a = 3, ..., 3 + A_t \\ s_a^{RI}(0) & \text{otherwise} \end{cases}$$

where r is the RI coverage and $e_{2,I}$ is the per-dose efficacy of IPV against serotype 2, which is set at 0.6 [9].

C.1.4 Forward projections for SIAs

Forward projections for SIA are assumed to occur according to the plans of the GPEI (Figure XXIV and Figure XXV) with coverage estimated as in Section B and shown in Figure XXI and Figure XXII for Nigeria and Pakistan respectively. Projections thus account for the presence of under-vaccinated communities within states.

Forward projections start with different susceptibility estimates for the under-vaccinated group and the better-vaccinated group, and account for SIA with IPV until June 2015, $s_{a,u}^{SIA(+I)}(0)$ and $s_{a,b}^{SIA(+I)}(0)$. The projections of susceptibility for SIA from the baseline are as follows. For a given month t in $1, ..., 9$ of the projections, population susceptibility in each group is updated as:

$$s_{a,b}^{SIA(+I)}(t) = s_{a,b}^{SIA(+I)}(t-1)(1 - c_b e_{2,tr})^{k_t}(1 - c_b e_{2,I})^{l_t} \qquad a = 1, ..., 35$$

$$s_{a,u}^{SIA(+I)}(t) = s_{a,u}^{SIA(+I)}(t-1)(1 - c_u e_{2,tr})^{k_t}(1 - c_u e_{2,I})^{l_t} \qquad a = 1, ..., 35$$

where k_t and l_t are respectively the number of SIA with tOPV and IPV planned during the month t. Note that a maximum of one tOPV or IPV campaign is planned per month (Figure XXIV and Figure XXV), and therefore k_t and l_t only take the values 0 or 1. Note also that only a few districts of Pakistan administered OPV and IPV at the same time (Figure XXV).

The estimate of susceptibility for the age class a accounting for the better- and under-vaccinated groups is then

$$s_a^{SIA(+I)}(t) = (1 - z_u)s_{a,b}^{SIA(+I)}(t) + z_u s_{a,u}^{SIA(+I)}(t).$$

where z_u is the proportion of the population in the under-vaccinated group.

C.1.5 Forward projections for routine immunisation and SIA

Finally, the estimated level of population immunity in the age class a at month t after baseline, $p_a^{(+I)}(t)$, is given by

$$p_a^{(+I)}(t) = 1 - s_a^{RI(+I)}(t)s_a^{SIA(+I)}(t)$$

and the final estimate for the population <36 months old is taken as the mean over the 36 age classes considered, $p_0^{(+I)}(t), p_1^{(+I)}(t), ..., p_{35}^{(+I)}(t)$.

C.1.6 Analysis of uncertainty in the projections

For each district of Nigeria and Pakistan, we quantified the uncertainty in the projections due to the propagation of uncertainty in 29 input parameters: the 12 baseline estimates of susceptibility due to SIA for each 3-month age class ($s_{age}^{SIA}(0)$ for *age* in 0–2, 3–5,..., 33–35 months), the 12 baseline

estimates of susceptibility due to RI for each 3-month age class ($s_{age}^{RI}(0)$ for age in 0–2, 3–5,..., 33–35 months), the SIA coverage in the better- and the under-vaccinated groups (c_b and c_u), the size of the under-vaccinated group (z_u), and the per-dose of tOPV and IPV efficacy against serotype-2 poliomyelitis ($e_{2,tr}$ and $e_{2,i}$).

Uncertainty analyses were conducted using the Latin Hypercube Sampling (LHS) [13] to efficiently and simultaneously sample from the posterior distributions of the estimated input parameters. Briefly, for each input parameter, its probability density function is stratified into m equiprobable intervals, and a single value is then selected from every interval and used only once. Here, for each district we created $m = 500$ input samples generated with the LHS and ran the projections for each input sample.

Note that in Pakistan, as SIA coverage was not estimated for Gilgit-Baltistan and Azad Jammu and Kashmir, we assumed a uniform distribution 0-100% for SIA coverage in the better-vaccinated population (assuming no under-vaccinated population).

C.1.7 Testing the assumption of non-independence between RI and SIA

Constructing a set of realistic hypotheses to assume non-independence between RI and SIA in the projections is not possible using the data available. However, to test to what extent non-independence between RI and SIA could lead to lower values of immunity in the projections, we ran a sensitivity analysis assuming that under-vaccinated groups had both lower SIA and RI coverage. We examined the extreme example wherein we assumed that individuals in the under-vaccinated group do not receive any doses of tOPV through RI (adjusting the estimated immunity from RI in the better-vaccinated population accordingly so that the mean RI immunity in the district remained the same). Note that in this extreme scenario, maintaining the mean RI estimate at the district level to be the same is not always possible, because the size of the under- and better-vaccinated groups is fixed, and thus a RI coverage >100% in the better-vaccinated group would be needed to compensate for the strong hypothesis that the under-vaccinated group does not received any dose through RI. This limitation leads to bigger differences in the projected immunity in districts where RI is high (e.g. South of Nigeria and Sindh & KPK of Pakistan).

As expected, this scenario resulted in lower projections everywhere, but the difference was higher in districts with high RI coverage. In Nigeria, those districts were mostly in the South, whereas in Pakistan, they were in parts of Sindh and KPK (Table III). The projections in the remainder of Nigeria and Pakistan remain similar to the main projections (Table III).

C.1.8 Forward projections for serotype-1 population immunity

For Pakistan, we also did forward projections of population immunity to serotype 1 at the moment of the switch, proceeding as described above for serotype 2. The projections account for the use of IPV in SIA and RI, and we used the following estimate of IPV efficacy against serotype 1: $e_{1,I} = 0.46$ [9].

To split the baseline estimates of susceptibility to serotype 1 into an estimate for the better-vaccinated group and an estimate for the under-vaccinated group (to start the projections with different estimates for the two groups), we used equation (2) and replaced $e_{2,tr}$ by 0.25, a mid-point between the efficacy estimates used for tOPV (0.125), bOPV (0.234) and mOPV1 (0.345) (i.e. we did not distinguish between the different OPV formulations that induce protection against serotype 1, and used a single estimate of efficacy for the three of them and estimated a single number of doses).

C.2 Supplementary Results

Projected estimates of serotype-2 population immunity in Nigeria and Pakistan at the moment of OPV2 withdrawal (April 2016) based on the planned SIAs (Figure XXIV and Figure XXV) are shown in Figure 4 of the main text (95% uncertainty intervals are given in Figure XXVI and Figure XXVII).

For Pakistan, projections of immunity for the planned SIAs and assuming improvements in access to vaccination (as described in Figure XXIII) are shown in Figure XXIX.

In Pakistan, wild poliovirus type 1 is still circulating and therefore ensuring high serotype-1 population immunity is also essential. We therefore tested alternative scenarios where different combinations of vaccine types were used (Figure XXXA). Projected immunity estimates for serotype 1 and serotype 2 at the moment of OPV2 withdrawal for those scenarios are presented in Figure XXXB. We also projected immunity estimates for the same scenarios and assuming improved access to SIA in all of Karachi, Peshawar and FATA (North and South Waziristan, Kurram and Khyber), as detailed in Figure XXIII. The projections for the scenarios with improved access are shown in Figure XXXC.

D Supplementary Figures and Tables

Figure I: Maps of Nigeria (top) and Pakistan (bottom), with the first-level administrative boundaries and their names. The publication of this map does not imply the expression of any opinion whatsoever on the part of WHO concerning the legal status of any territory, city or area or of its authorities, or concerning the delimitation of its frontiers or boundaries.

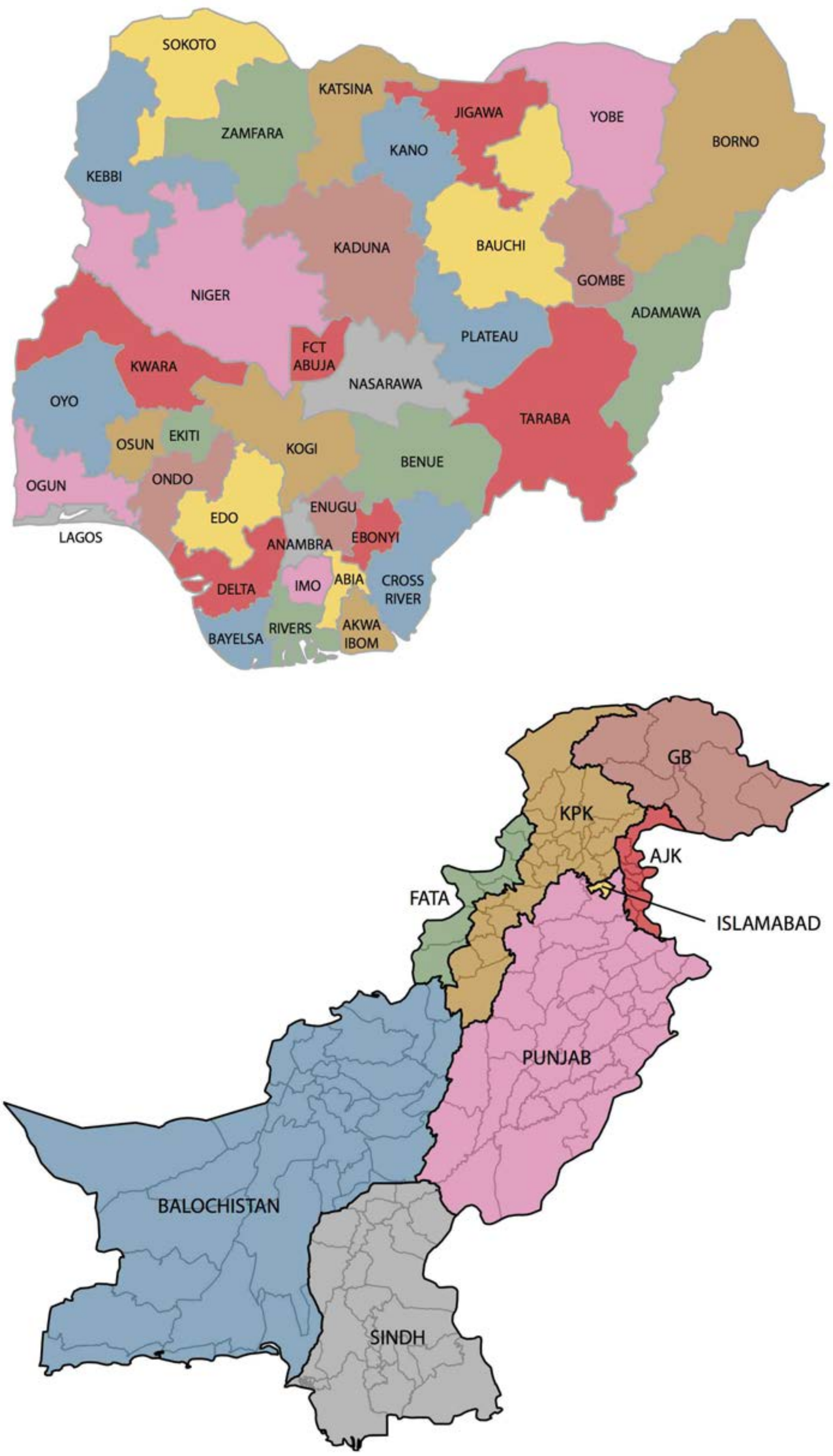

Figure II: Estimates of routine immunisation coverage in Nigeria (based on data from DHS for 3 doses of DTP). The publication of this map does not imply the expression of any opinion whatsoever on the part of WHO concerning the legal status of any territory, city or area or of its authorities, or concerning the delimitation of its frontiers or boundaries.

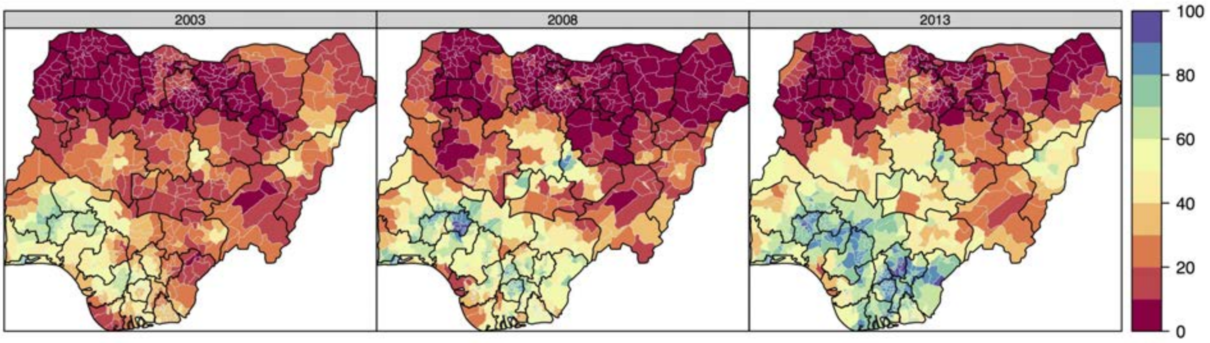

Figure III: Estimates of routine immunisation coverage (3 doses of tOPV) in Pakistan. The publication of this map does not imply the expression of any opinion whatsoever on the part of WHO concerning the legal status of any territory, city or area or of its authorities, or concerning the delimitation of its frontiers or boundaries.

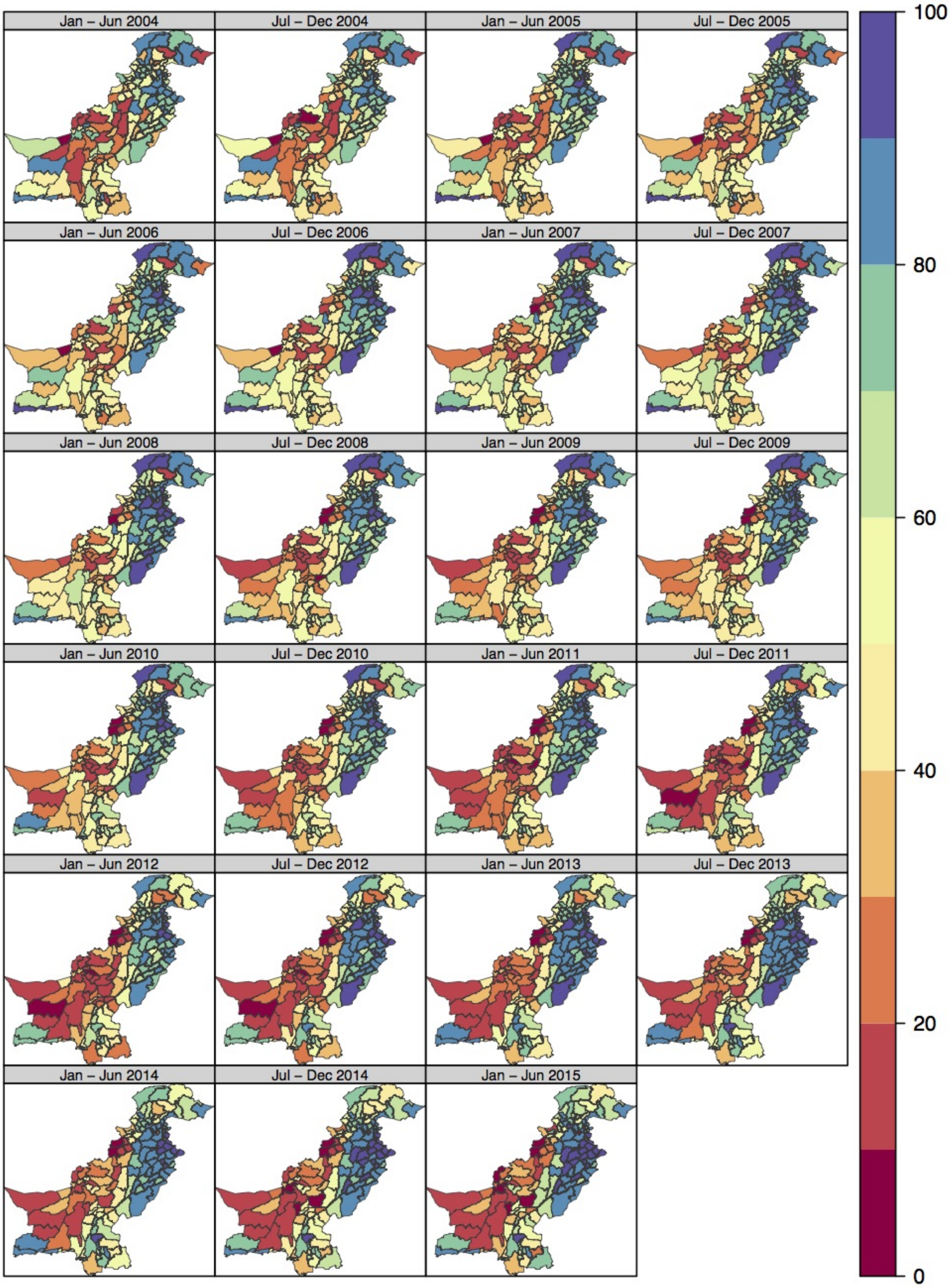

Figure IV: Number of tOPV SIAs per district and 6-month period in Nigeria. The publication of this map does not imply the expression of any opinion whatsoever on the part of WHO concerning the legal status of any territory, city or area or of its authorities, or concerning the delimitation of its frontiers or boundaries.

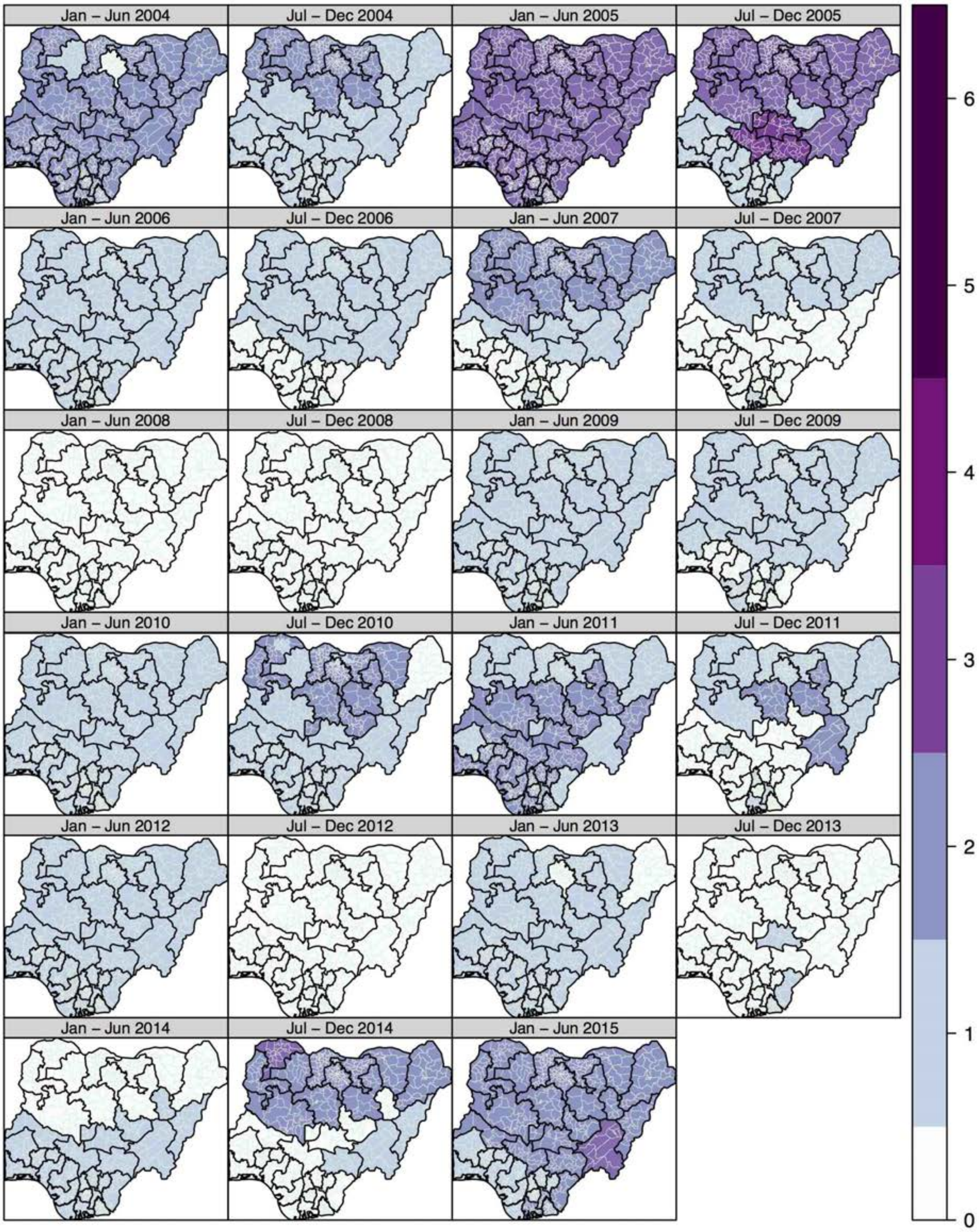

Figure V: Number of tOPV SIAs per district and 6-month period in Pakistan. The publication of this map does not imply the expression of any opinion whatsoever on the part of WHO concerning the legal status of any territory, city or area or of its authorities, or concerning the delimitation of its frontiers or boundaries.

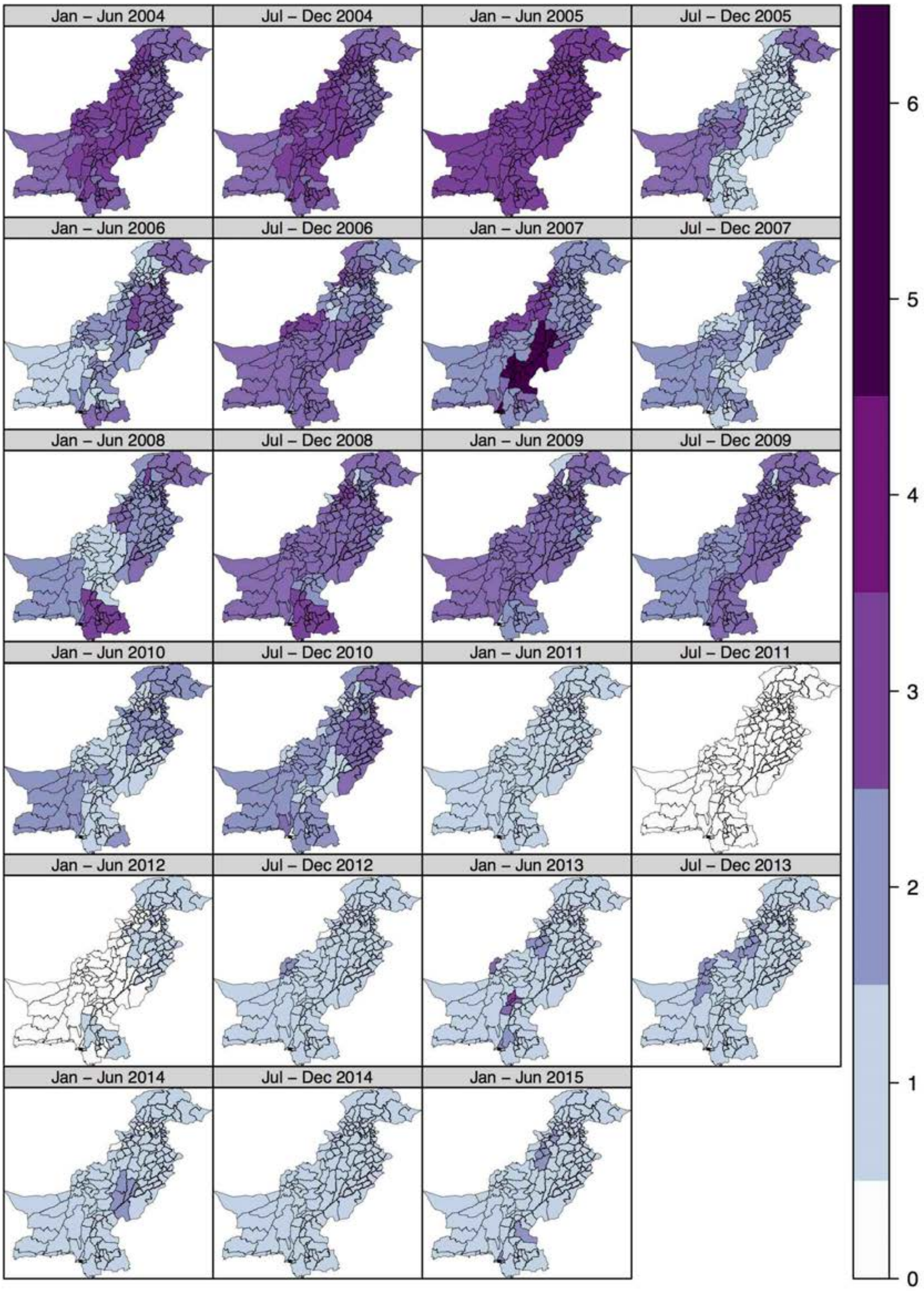

Figure VI: Annual proportion of SIAs with tOPV per district in Nigeria. The publication of this map does not imply the expression of any opinion whatsoever on the part of WHO concerning the legal status of any territory, city or area or of its authorities, or concerning the delimitation of its frontiers or boundaries.

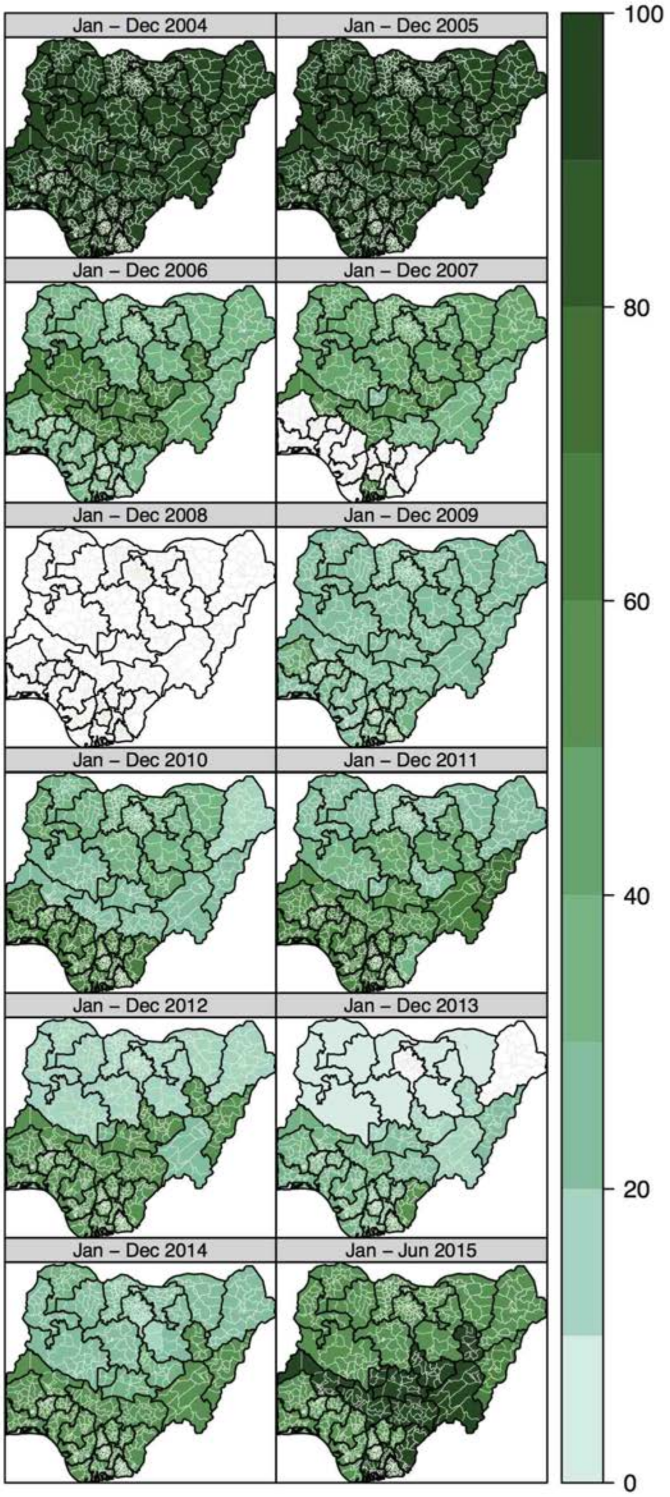

Figure VII: Annual proportion of SIAs with tOPV per district in Pakistan. The publication of this map does not imply the expression of any opinion whatsoever on the part of WHO concerning the legal status of any territory, city or area or of its authorities, or concerning the delimitation of its frontiers or boundaries.

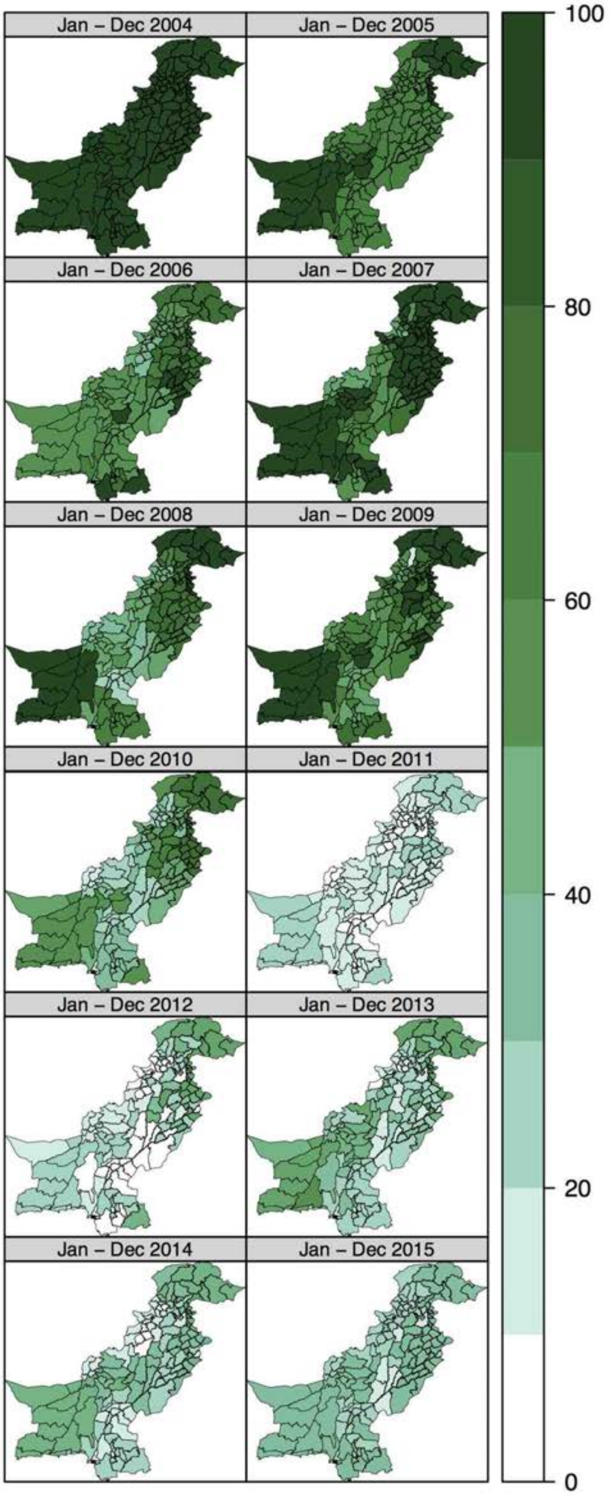

Figure VIII: 95% credible intervals for the estimates of serotype-2 population immunity per district and 6-month period in Nigeria. The publication of this map does not imply the expression of any opinion whatsoever on the part of WHO concerning the legal status of any territory, city or area or of its authorities, or concerning the delimitation of its frontiers or boundaries.

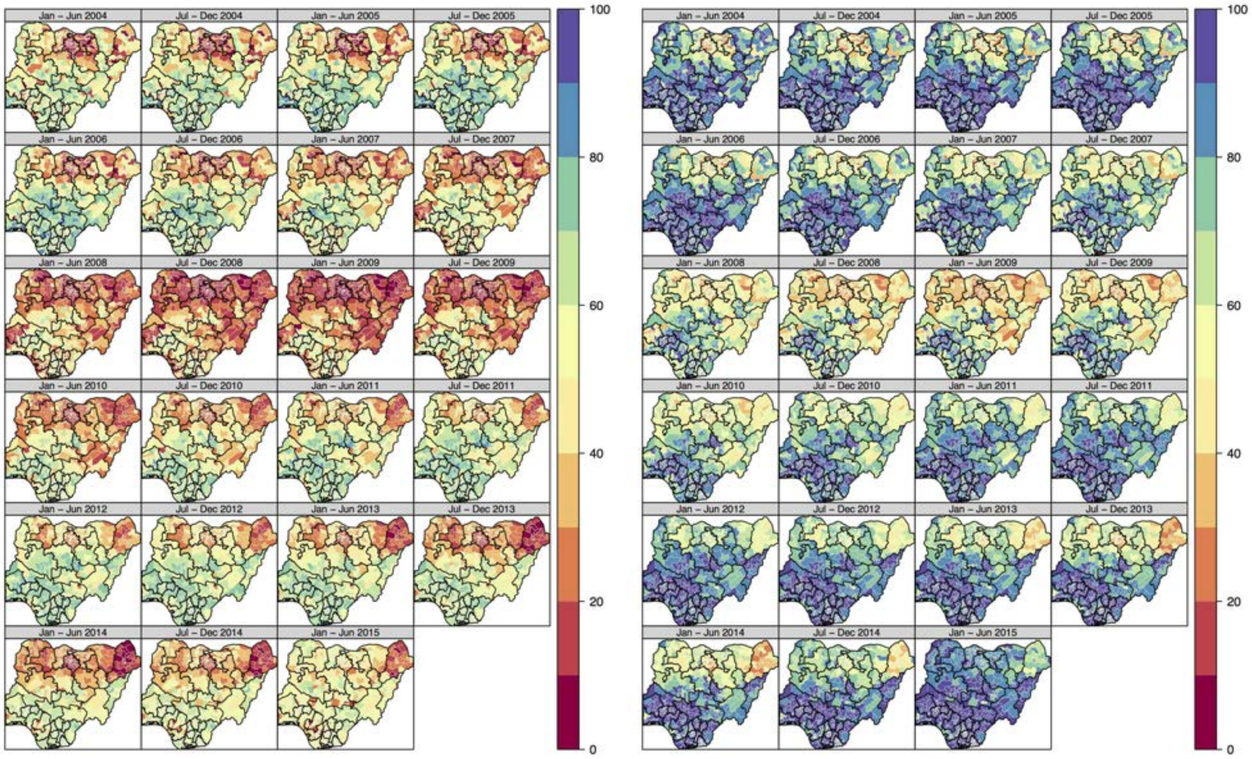

Figure IX: 95% credible intervals for the estimates of serotype-2 population immunity per district and 6-month period in Pakistan. The publication of this map does not imply the expression of any opinion whatsoever on the part of WHO concerning the legal status of any territory, city or area or of its authorities, or concerning the delimitation of its frontiers or boundaries.

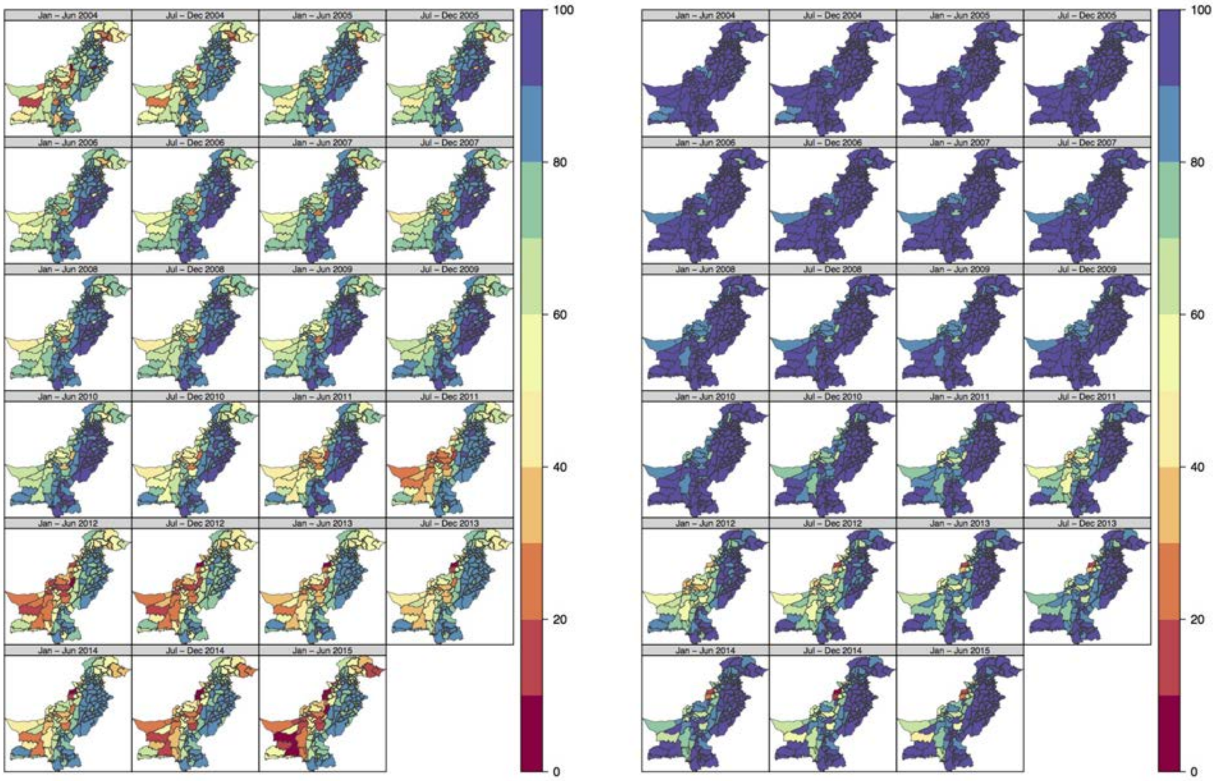

Figure X: Number of SIAs with IPV (first line) and estimates of serotype-2 population immunity not accounting for (second line) and accounting for SIAs with IPV (third line) in Nigeria from Jan-Jun 2014 to Jan-Jun 2015. The publication of this map does not imply the expression of any opinion whatsoever on the part of WHO concerning the legal status of any territory, city or area or of its authorities, or concerning the delimitation of its frontiers or boundaries.

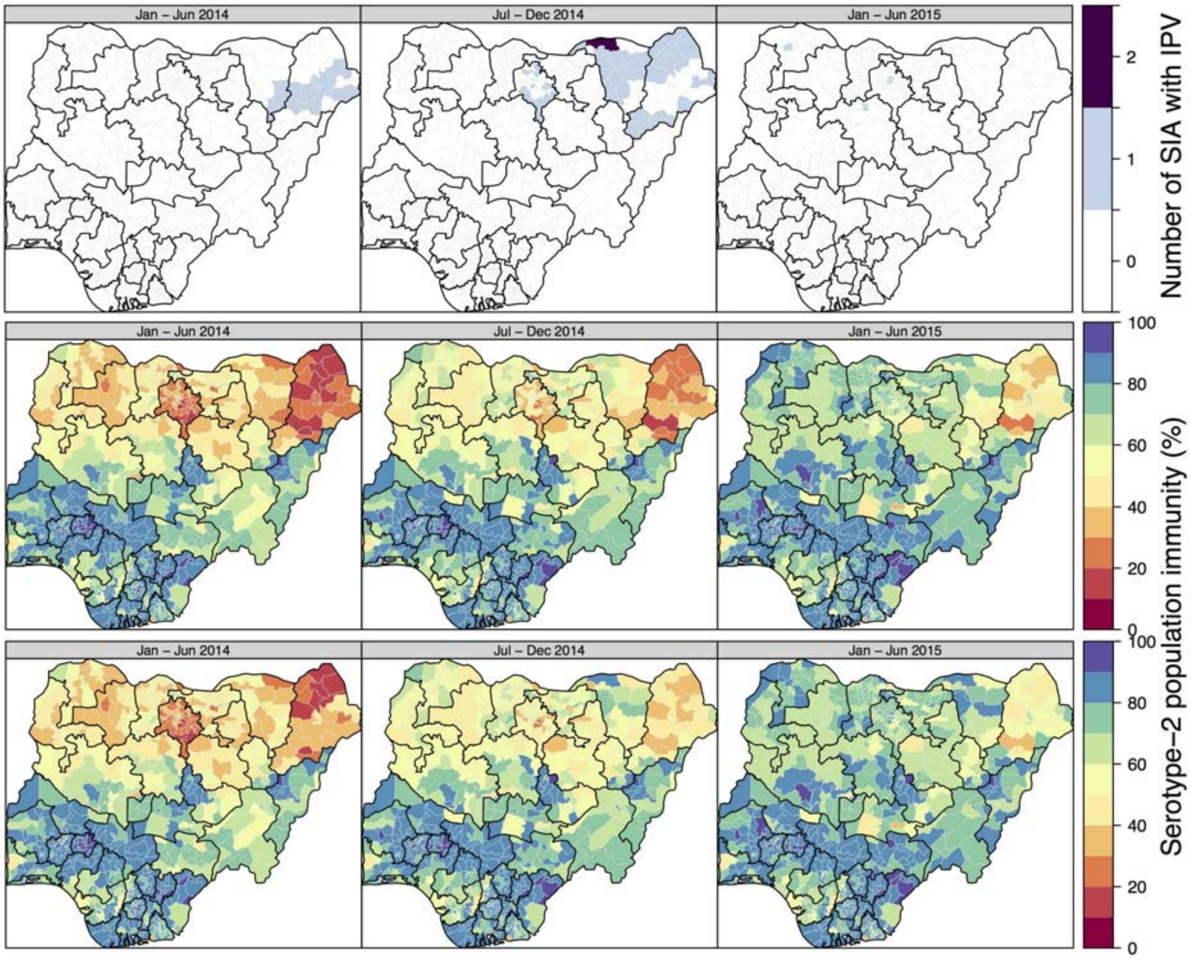

Figure XI: Proportion of the population targeted by the SIA with IPV (first line) and estimates of serotype-2 population immunity not accounting for (second line) and accounting for SIAs with IPV (third line) in Pakistan from Jan-Jun 2014 to Jan-Jun 2015. Note that maximum one SIA with IPV is implemented in each 6-month period. The publication of this map does not imply the expression of any opinion whatsoever on the part of WHO concerning the legal status of any territory, city or area or of its authorities, or concerning the delimitation of its frontiers or boundaries.

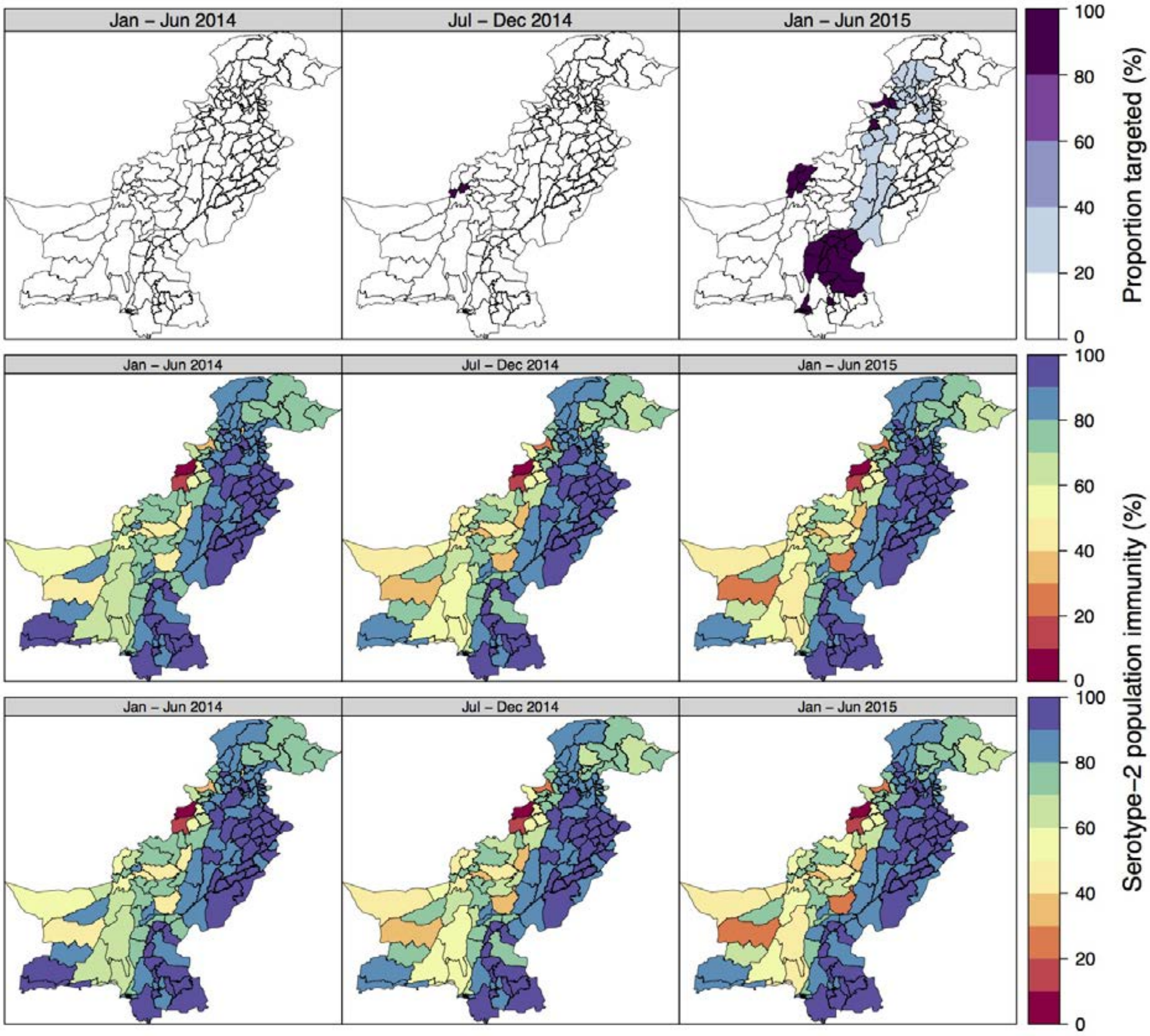

Figure XII: Estimates of serotype-2 population immunity in children <36 months per state and 6-month period in Afghanistan, from Jan-Jun 2010 to Jan-Jun 2015. The publication of this map does not imply the expression of any opinion whatsoever on the part of WHO concerning the legal status of any territory, city or area or of its authorities, or concerning the delimitation of its frontiers or boundaries.

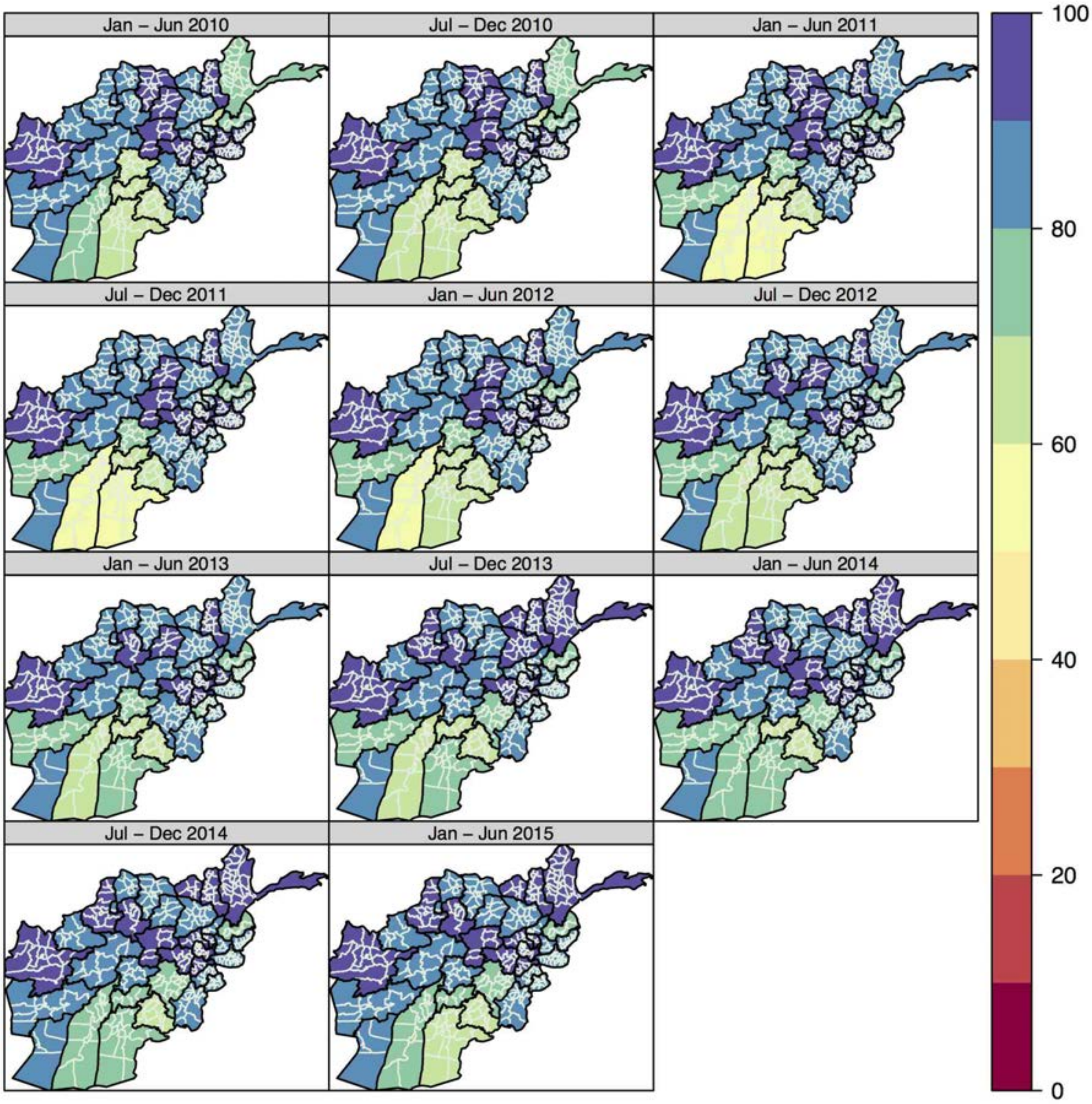

Figure XIII: Estimates of serotype-2 population immunity in children <36 months per state and 6-month period in Cameroon, from Jan-Jun 2010 to Jan-Jun 2015. The publication of this map does not imply the expression of any opinion whatsoever on the part of WHO concerning the legal status of any territory, city or area or of its authorities, or concerning the delimitation of its frontiers or boundaries.

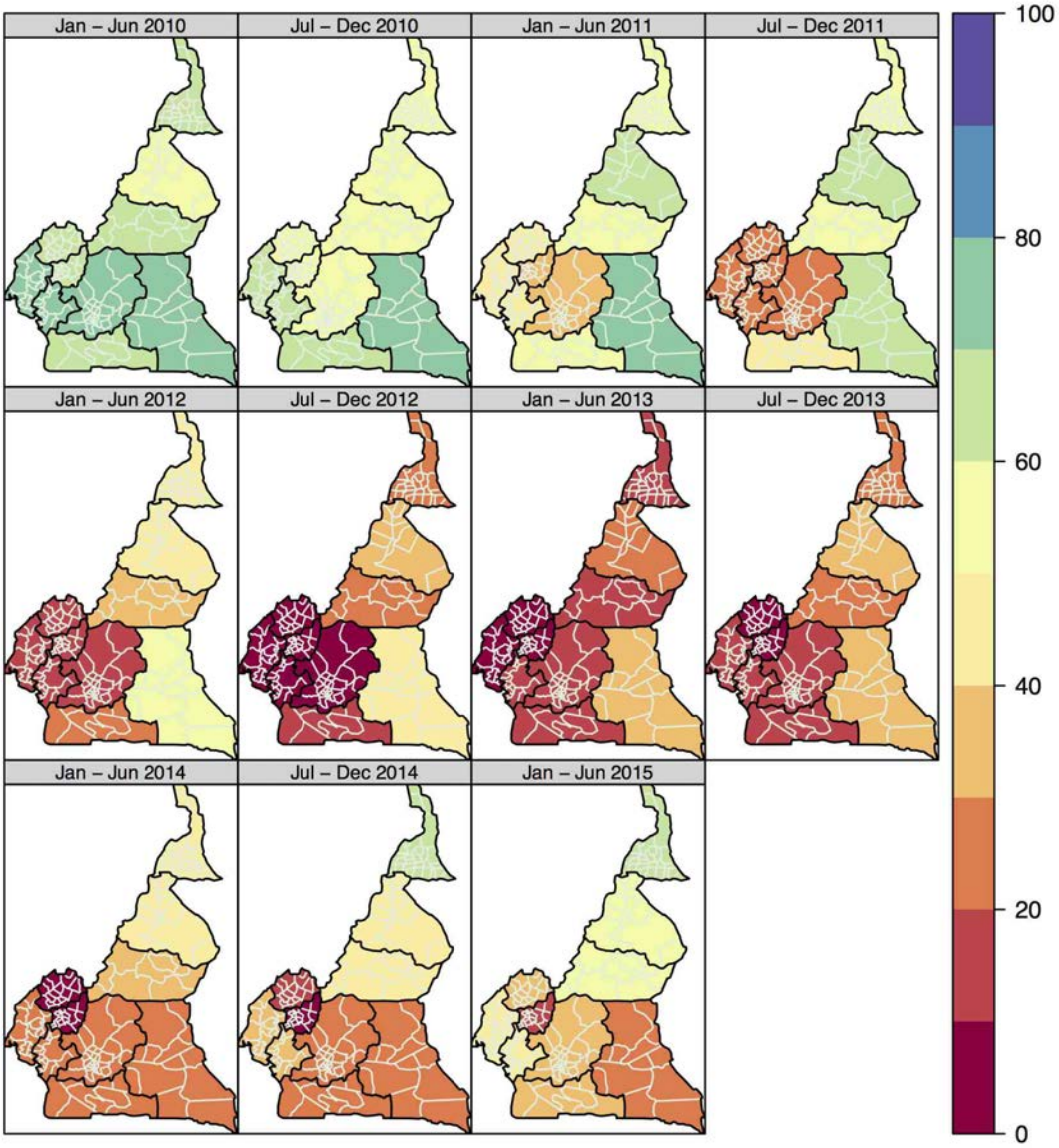

Figure XIV: Estimates of serotype-2 population immunity in children <36 months per state and 6-month period in Chad, from Jan-Jun 2010 to Jan-Jun 2015. The publication of this map does not imply the expression of any opinion whatsoever on the part of WHO concerning the legal status of any territory, city or area or of its authorities, or concerning the delimitation of its frontiers or boundaries.

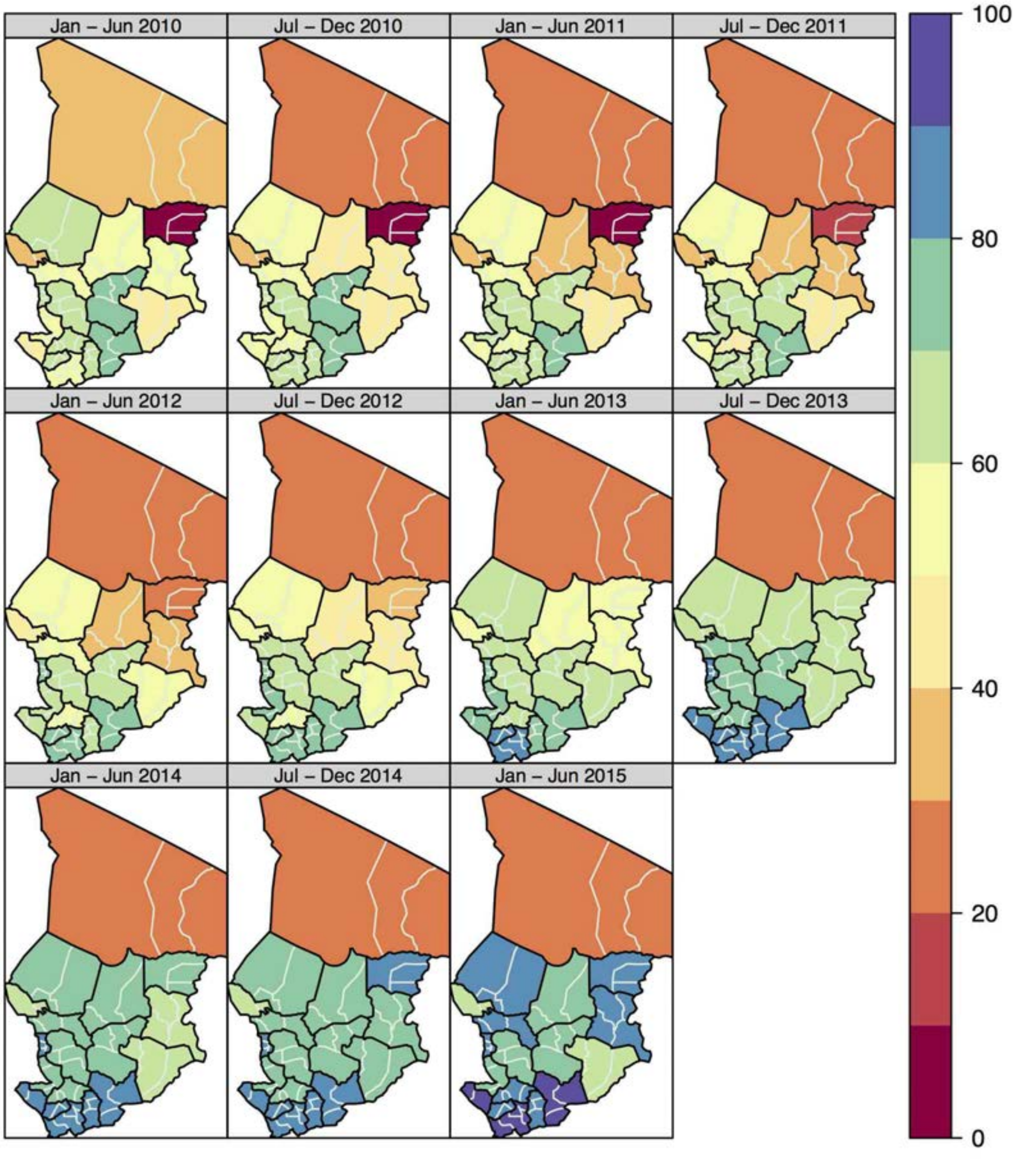

Figure XV: Estimates of serotype-2 population immunity in children <36 months per state and 6-month period in Democratic Republic of Congo, from Jan-Jun 2010 to Jan-Jun 2015. The publication of this map does not imply the expression of any opinion whatsoever on the part of WHO concerning the legal status of any territory, city or area or of its authorities, or concerning the delimitation of its frontiers or boundaries.

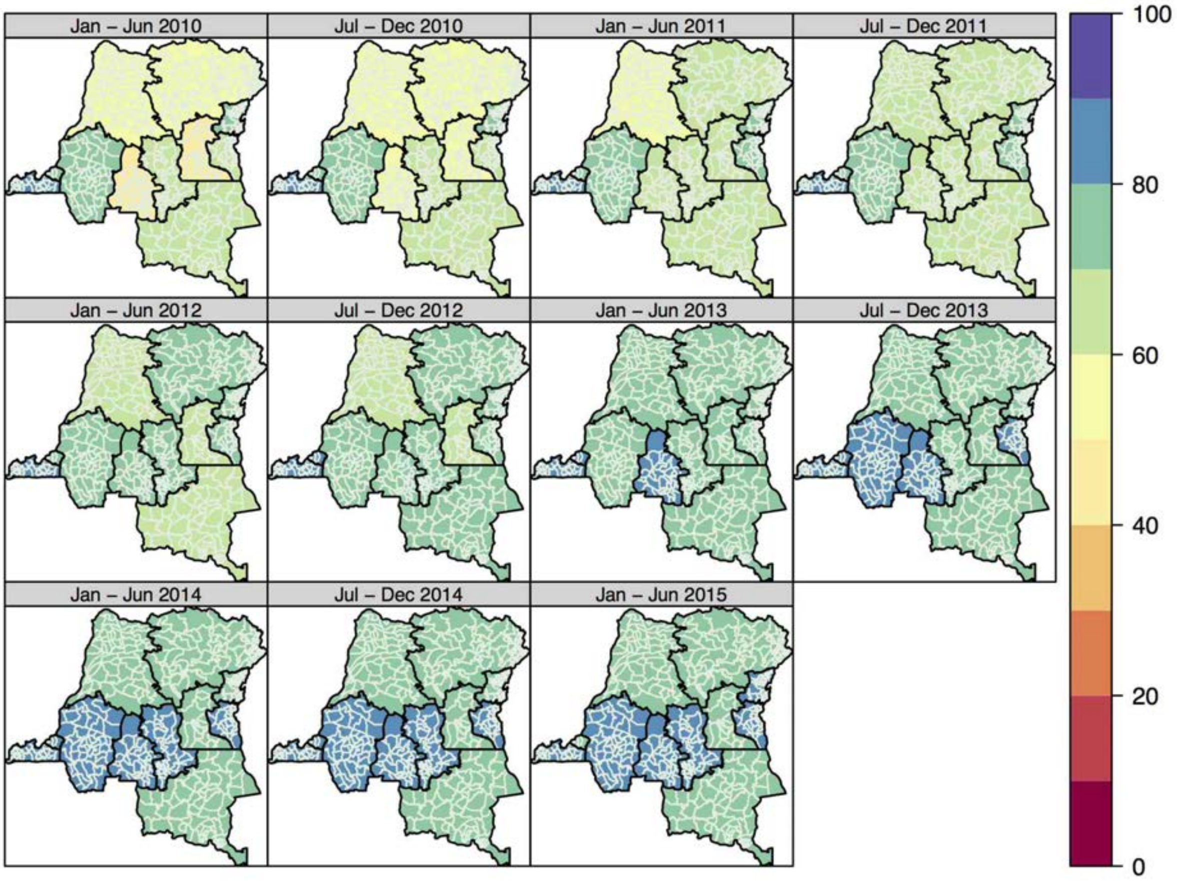

Figure XVI: Estimates of serotype-2 population immunity in children <36 months per state and 6-month period in Niger, from Jan-Jun 2010 to Jan-Jun 2015. The publication of this map does not imply the expression of any opinion whatsoever on the part of WHO concerning the legal status of any territory, city or area or of its authorities, or concerning the delimitation of its frontiers or boundaries.

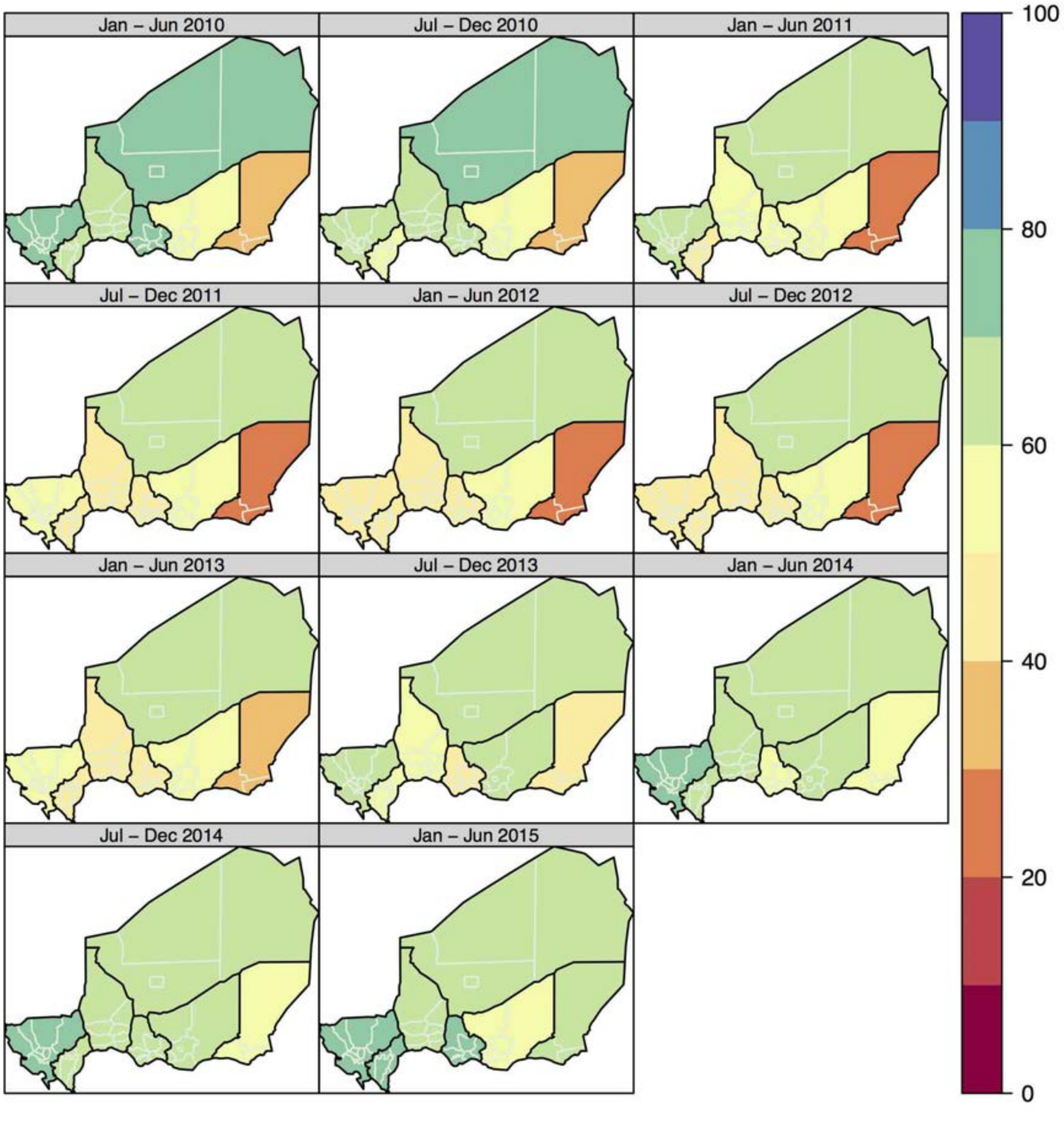

Figure XVII: Estimates of serotype-2 population immunity in children <36 months per state and 6-month period in South Sudan, from Jan-Jun 2010 to Jan-Jun 2015. The publication of this map does not imply the expression of any opinion whatsoever on the part of WHO concerning the legal status of any territory, city or area or of its authorities, or concerning the delimitation of its frontiers or boundaries.

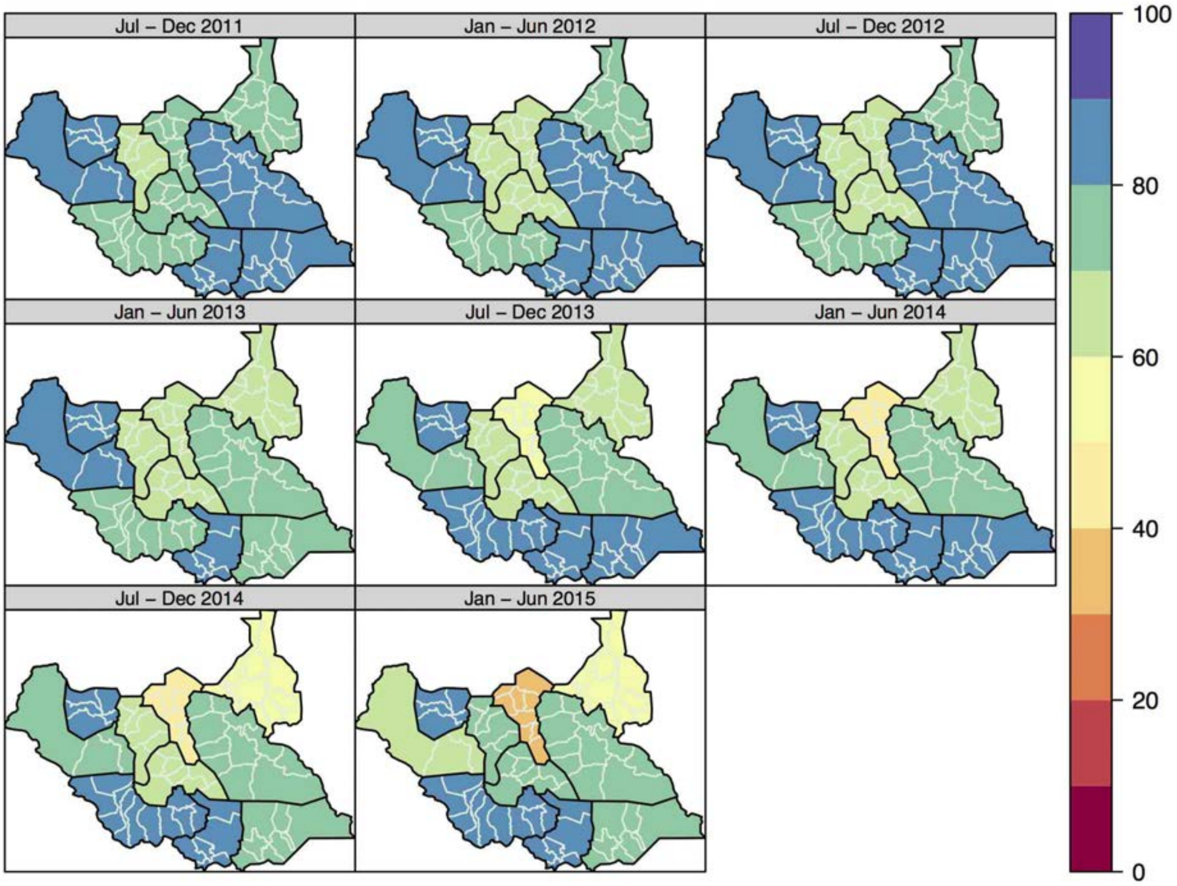

Figure XVIII: Estimated serotype-2 population immunity in children <36 months of age for Jan-Jun 2015 in countries that have reported cVDPV2 cases between 2013 and 2015. (A) Countries in the African Region: Cameroon, Chad, DRC, Niger, Nigeria and South Sudan. (B) Countries in the Eastern Mediterranean Region: Afghanistan and Pakistan. First administrative level boundaries are shown in light grey, lakes at borders in pale blue, and disputed borders and areas are shown in dark grey. The publication of this map does not imply the expression of any opinion whatsoever on the part of WHO concerning the legal status of any territory, city or area or of its authorities, or concerning the delimitation of its frontiers or boundaries. WHO does not endorse or approve the use of sub-national boundaries in this map. (C) Geographic location of these countries. (D) Annual number of reported cVDPV2 cases since 2010 by country as of 20 October 2015.

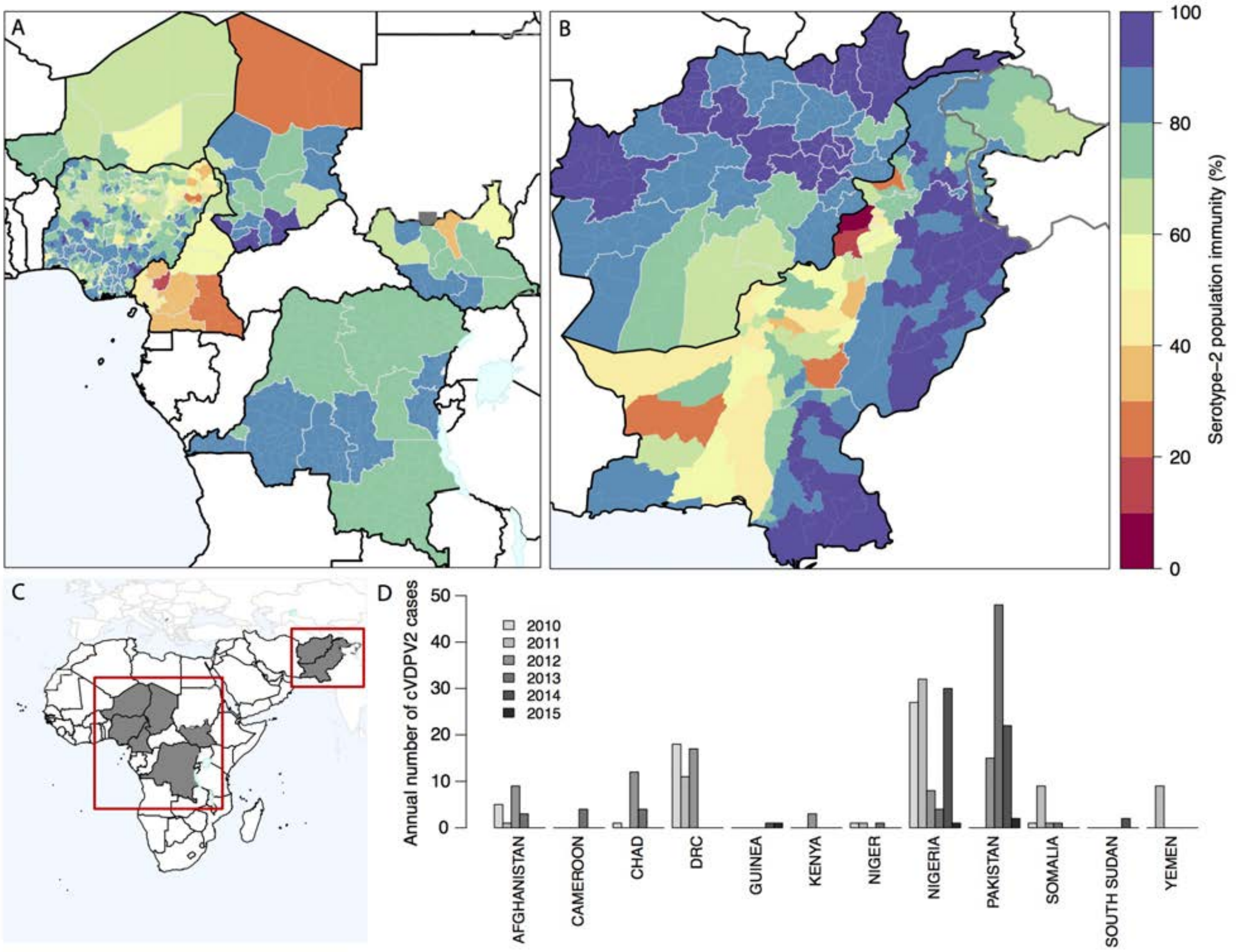

Figure XIX: 95% credible intervals for the estimates of serotype-2 population immunity in Jan-Jun 2015 in countries of the African Region (top) and countries of the East Mediterranean Region (bottom). The publication of this map does not imply the expression of any opinion whatsoever on the part of WHO concerning the legal status of any territory, city or area or of its authorities, or concerning the delimitation of its frontiers or boundaries.

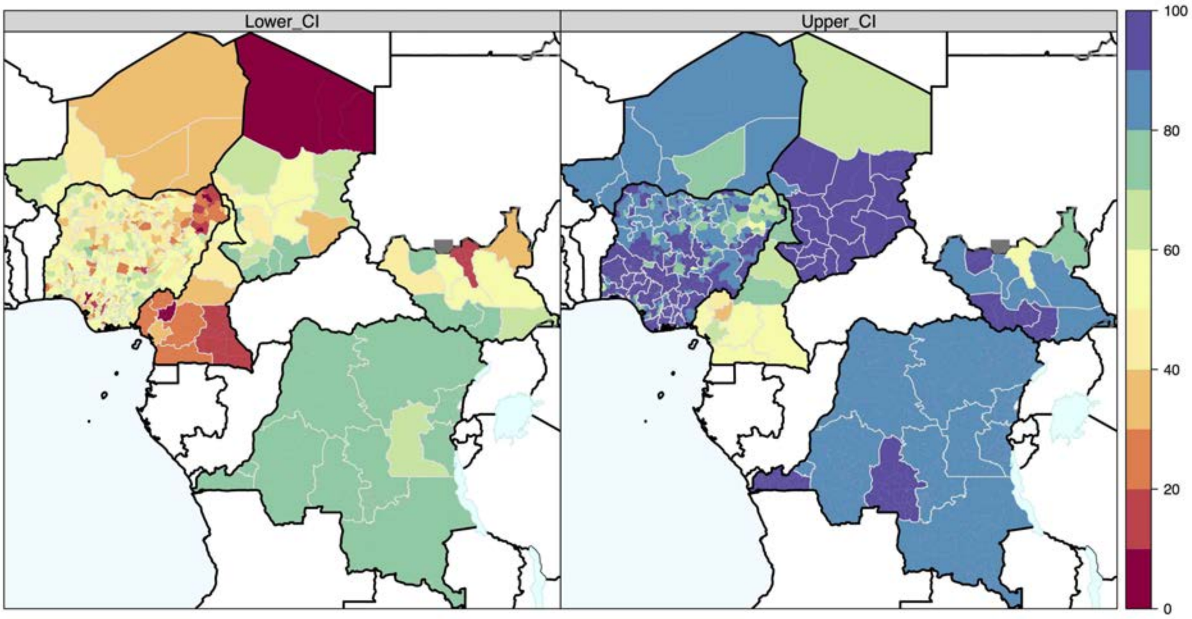

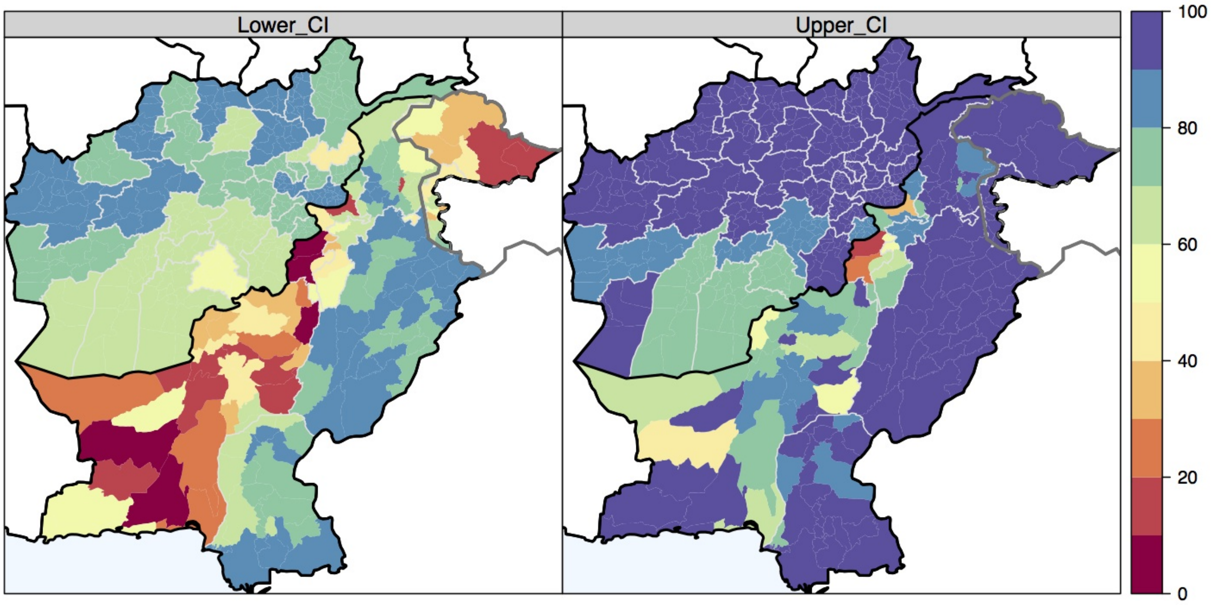

Figure XX: Number of tOPV (top) and IPV (bottom) SIA planned between July 2015 and March 2016 in countries that have reported cVDPV2 cases between 2013 and 2015 other than Nigeria and Pakistan. The publication of this map does not imply the expression of any opinion whatsoever on the part of WHO concerning the legal status of any territory, city or area or of its authorities, or concerning the delimitation of its frontiers or boundaries.

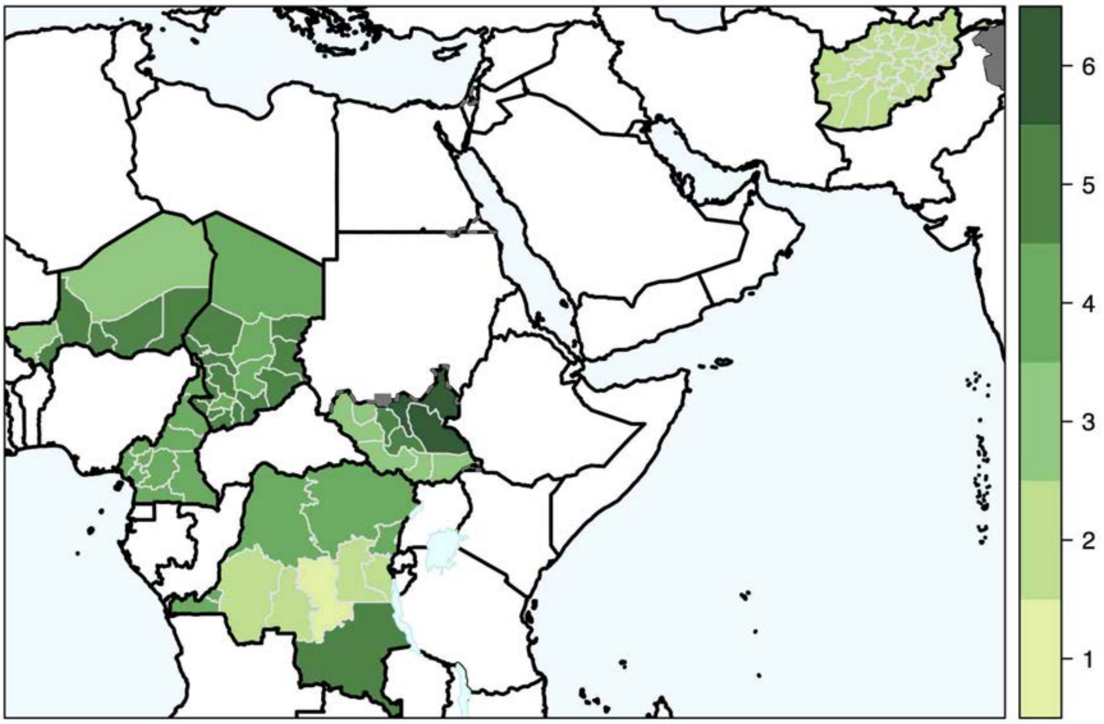

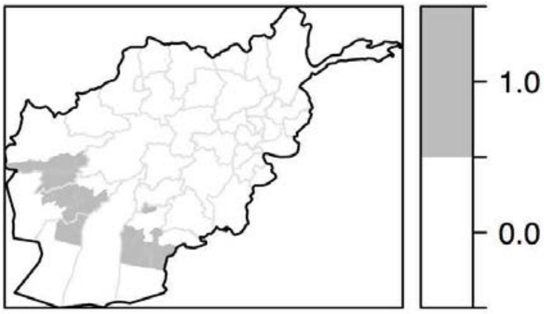

Figure XXI: (A,D) Estimated size of the under-vaccinated groups within states in Nigeria. (B,E) Estimated SIA coverage in the better-vaccinated group. (C,F) Estimated SIA coverage in the under-vaccinated group. (A,B,C) Correspond to estimates for January 2014 – June 2015, whereas (D,E,F) correspond to estimates for January 2009 – December 2013. (G,H,I) 95% credible intervals for the SIA coverage estimates for January 2014 – June 2015. The publication of this map does not imply the expression of any opinion whatsoever on the part of WHO concerning the legal status of any territory, city or area or of its authorities, or concerning the delimitation of its frontiers or boundaries.

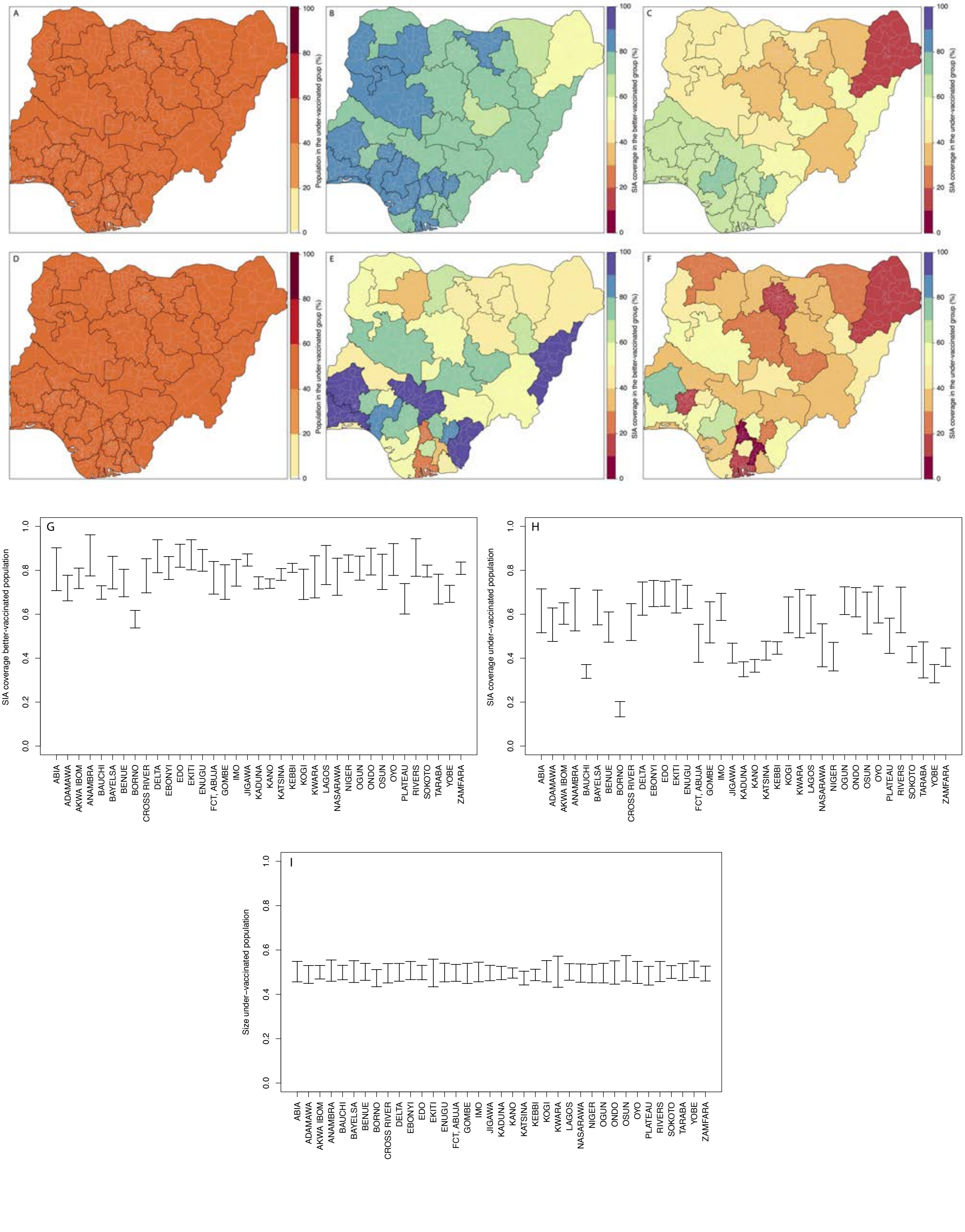

Figure XXII: (A,D) Estimated size of the under-vaccinated groups within regions in Pakistan. White indicates no evidence for the presence of an under-vaccinated group. (B,E) Estimated SIA coverage in the better-vaccinated group. (C,F) Estimated SIA coverage in the under-vaccinated group. (A,B,C) Correspond to estimates for January 2014 – June 2015, whereas (D,E,F) correspond to estimates for January 2009 – December 2013. Gray indicates that SIA coverage was not estimated in those districts. (G,H,I) 95% credible intervals for the SIA coverage estimates for January 2014 – June 2015. The publication of this map does not imply the expression of any opinion whatsoever on the part of WHO concerning the legal status of any territory, city or area or of its authorities, or concerning the delimitation of its frontiers or boundaries.

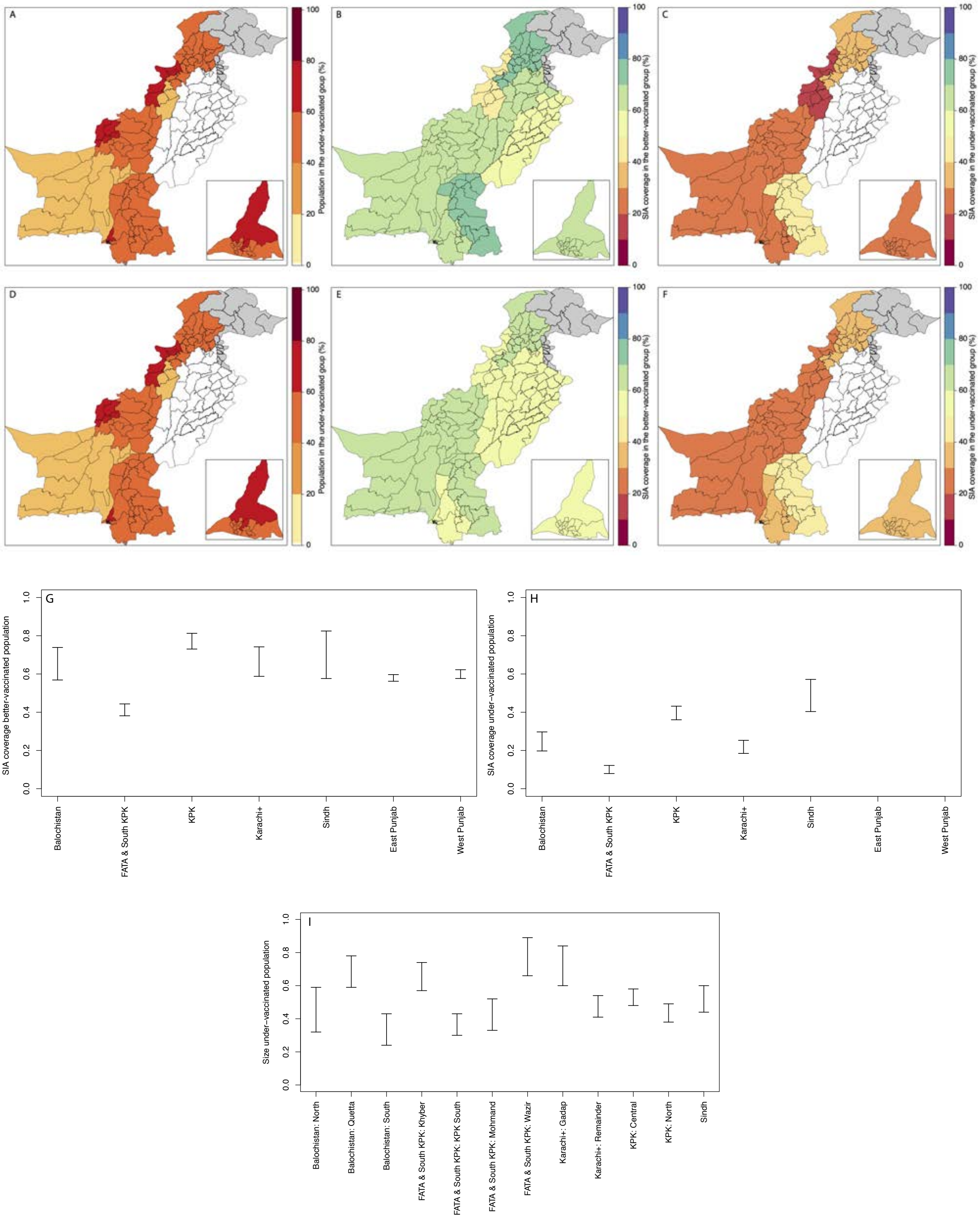

Figure XXIII: Percentage of the population in the under-vaccinated groups used in the alternative scenarios for the projections assuming improvements in access by vaccinators to SIAs during 2015 in all of Karachi (13%), Peshawar (2%) and FATA (North and South Waziristan, Kurram and Khyber, 50%). Gray indicates that SIA coverage was not estimated in those districts. The publication of this map does not imply the expression of any opinion whatsoever on the part of WHO concerning the legal status of any territory, city or area or of its authorities, or concerning the delimitation of its frontiers or boundaries.

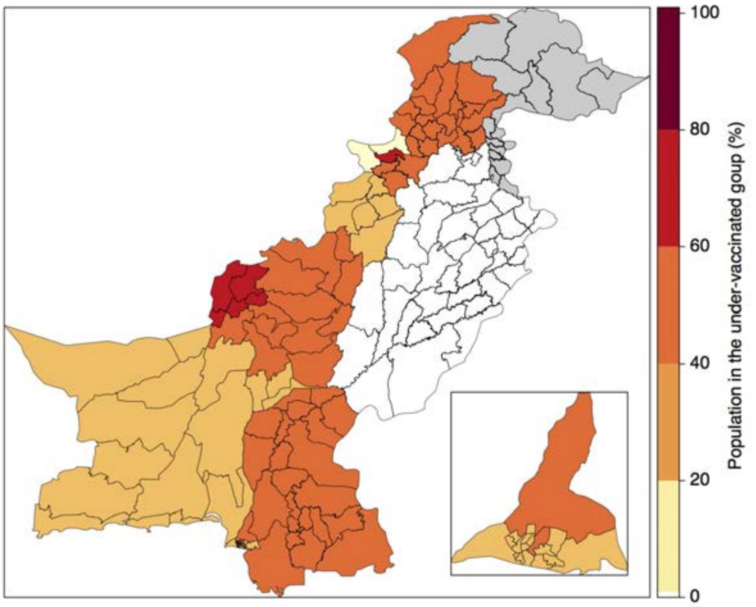

Figure XXIV: Planned SIAs with tOPV from July 2015 to March 2016 in Nigeria. The publication of this map does not imply the expression of any opinion whatsoever on the part of WHO concerning the legal status of any territory, city or area or of its authorities, or concerning the delimitation of its frontiers or boundaries.

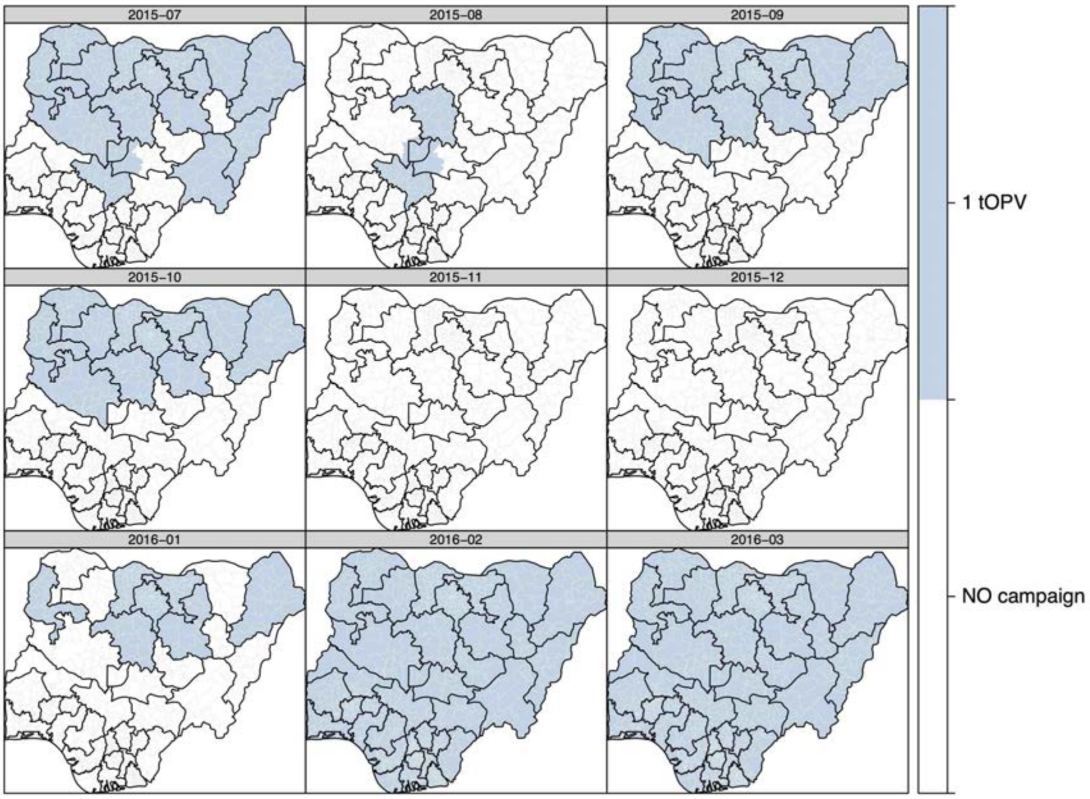

Figure XXV: Planned SIAs with tOPV, bOPV and IPV from July 2015 to March 2016 in Pakistan. The publication of this map does not imply the expression of any opinion whatsoever on the part of WHO concerning the legal status of any territory, city or area or of its authorities, or concerning the delimitation of its frontiers or boundaries.

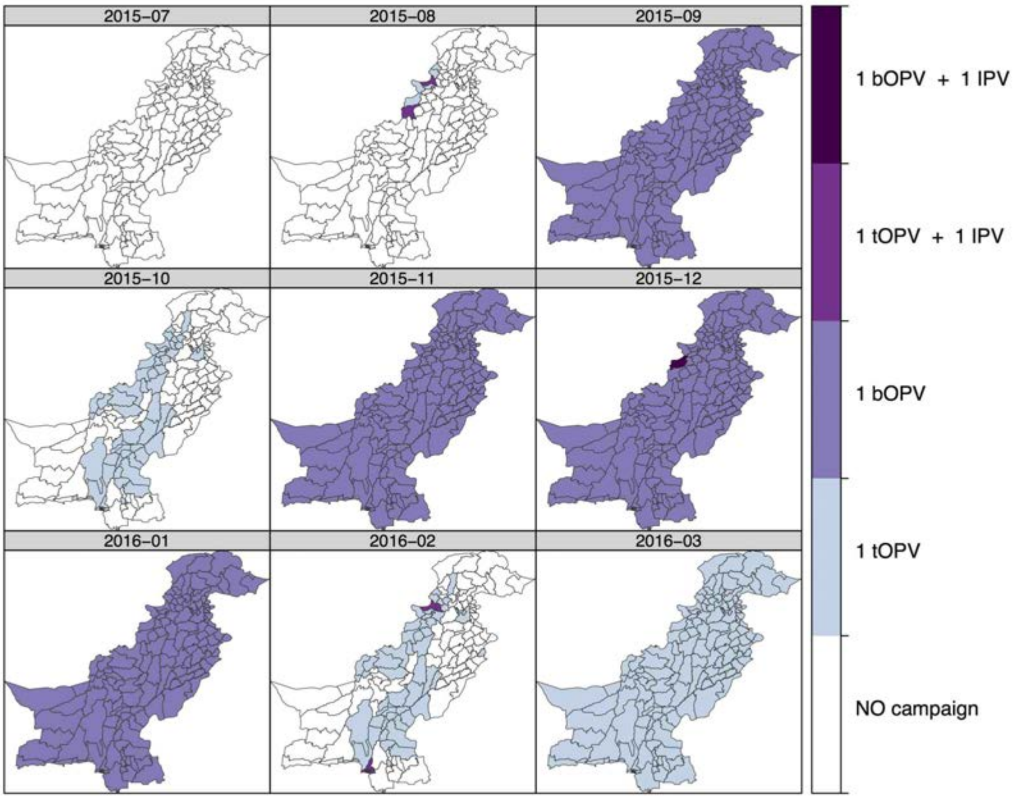

Figure XXVI: Lower and upper 95% uncertainty interval bounds of projected immunity against serotype 2 in April 2016 in Nigeria calculated through uncertainty analysis as described in Section C.1.6. The publication of this map does not imply the expression of any opinion whatsoever on the part of WHO concerning the legal status of any territory, city or area or of its authorities, or concerning the delimitation of its frontiers or boundaries.

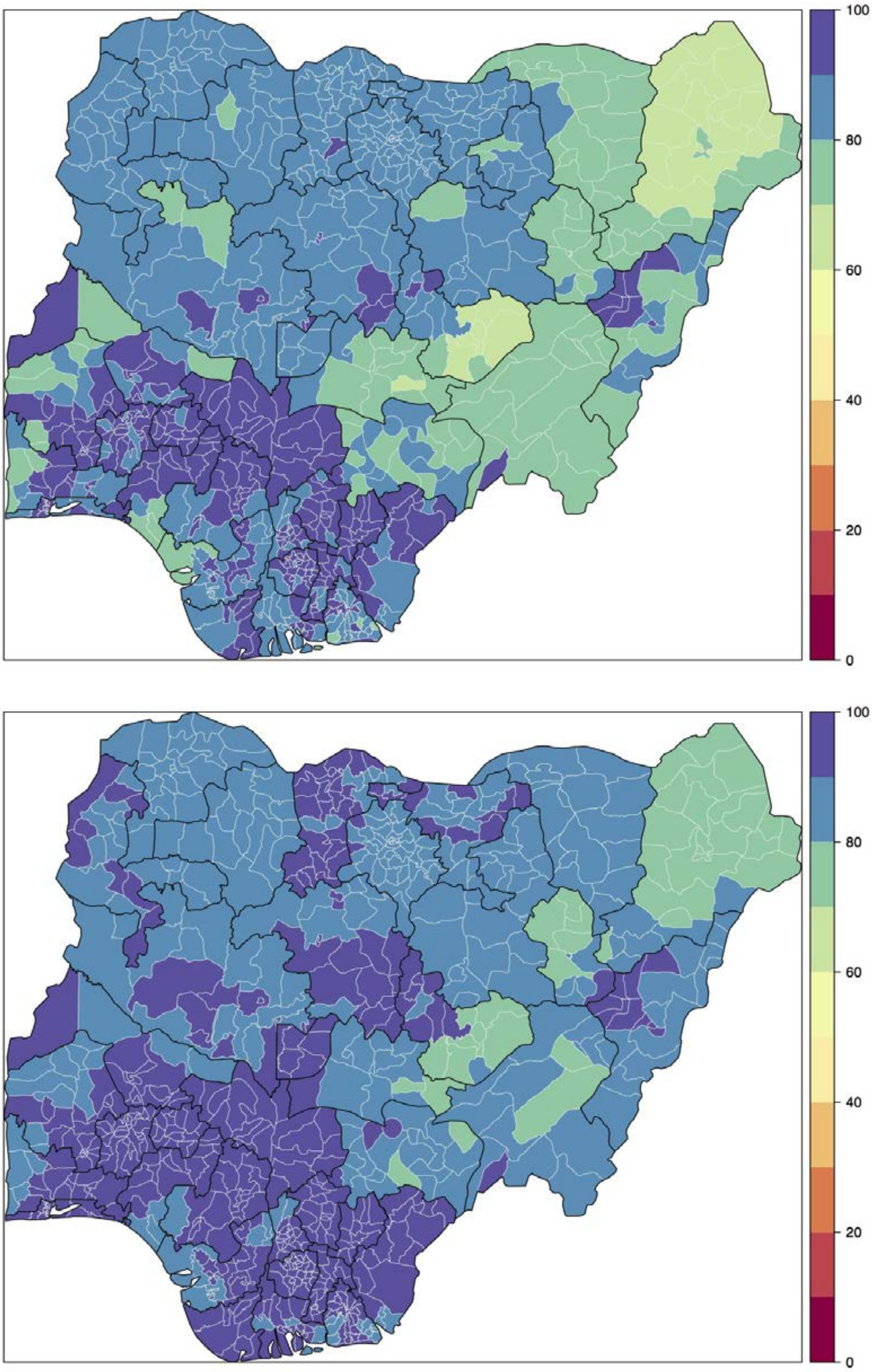

Figure XXVII: Lower and upper 95% uncertainty interval bounds of immunity against serotype 2 in April 2016 in Pakistan calculated through uncertainty analysis as described in Section C.1.6. The publication of this map does not imply the expression of any opinion whatsoever on the part of WHO concerning the legal status of any territory, city or area or of its authorities, or concerning the delimitation of its frontiers or boundaries.

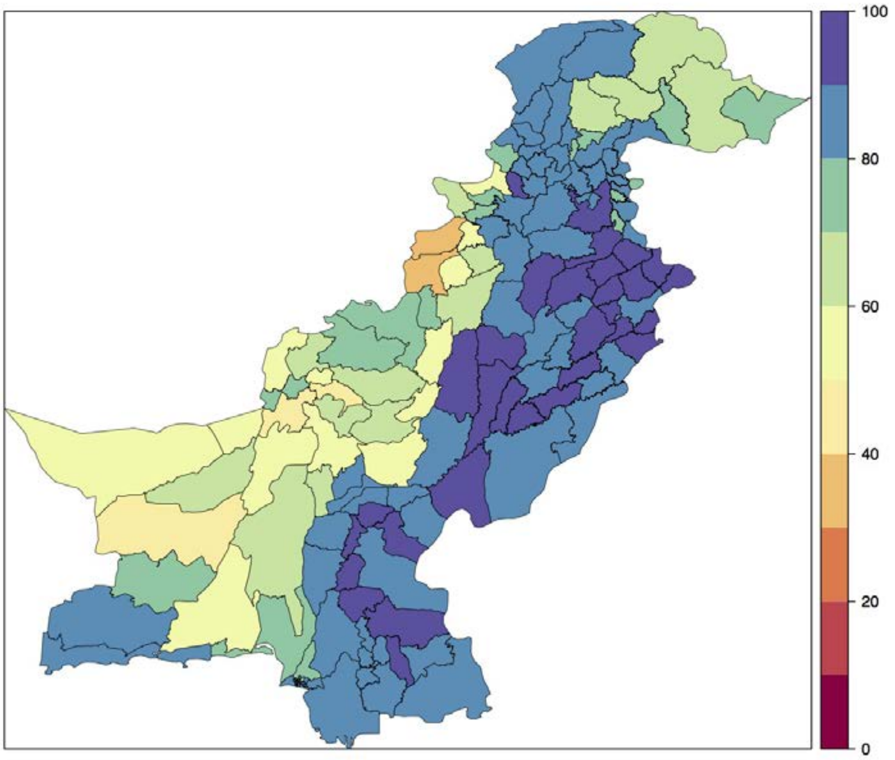

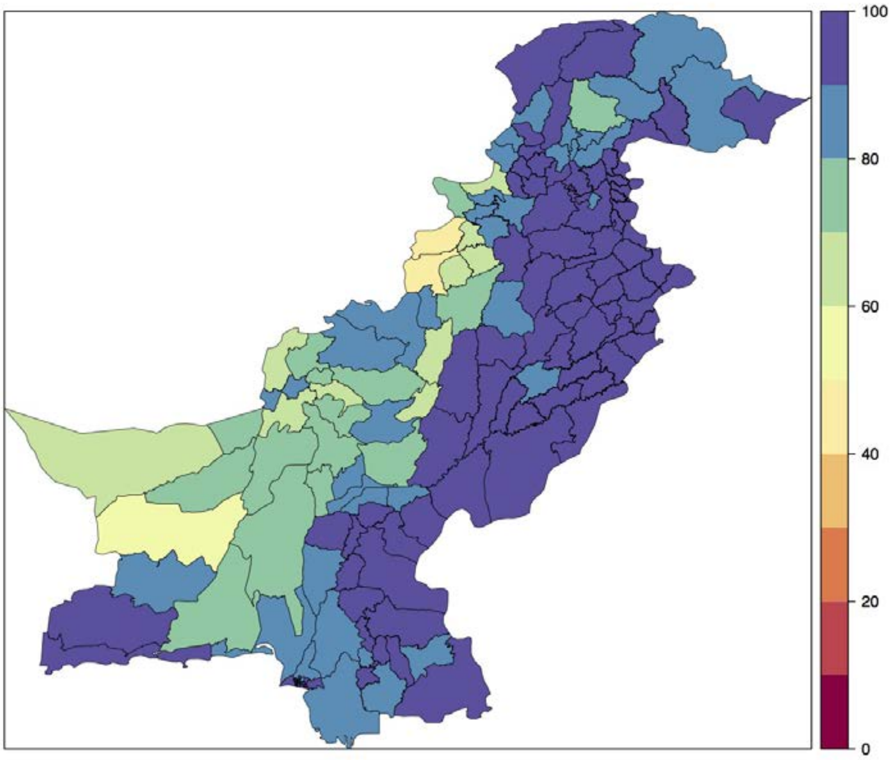

Figure XXVIII: Estimated serotype-1 population immunity in children <36 months of age for Jan-Jun 2015 in Pakistan. The publication of this map does not imply the expression of any opinion whatsoever on the part of WHO concerning the legal status of any territory, city or area or of its authorities, or concerning the delimitation of its frontiers or boundaries.

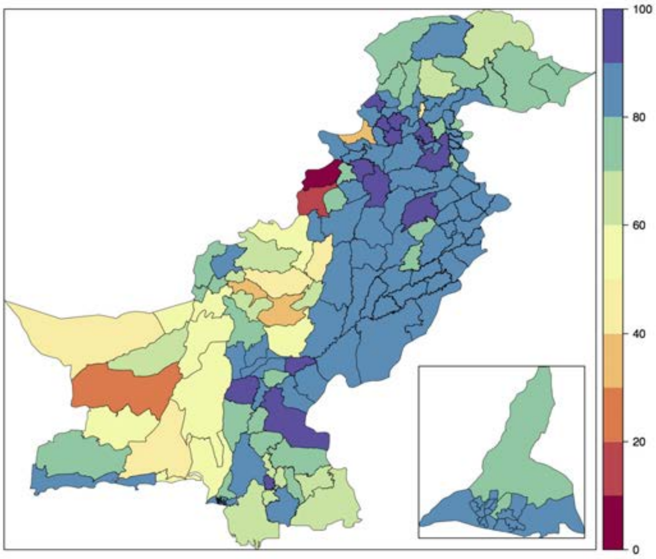

Figure XXIX: Projected immunity against serotypes 1 and 2 in April 2016 in Pakistan for planned SIAs (Figure XXV) assuming the same SIA coverage as that of Jan 2014 – June 2015 (top) and assuming improvements in SIA coverage as described in Figure XXIII (bottom). The publication of this map does not imply the expression of any opinion whatsoever on the part of WHO concerning the legal status of any territory, city or area or of its authorities, or concerning the delimitation of its frontiers or boundaries.

Serotype 1 | Serotype 2

Planned SIAs and SIA coverage similar to that of Jan 2014 – June 2015

Planned SIAs and assuming improvements in SIA coverage

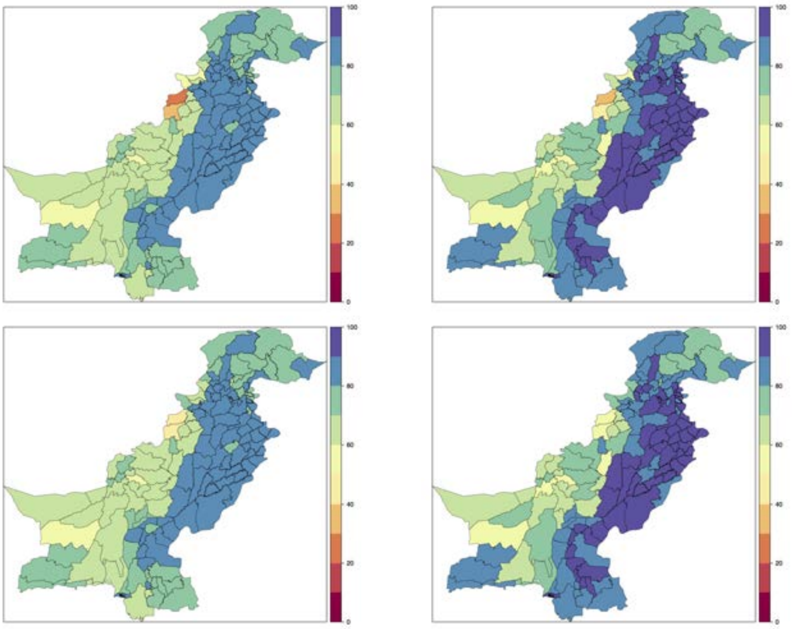

Figure XXX: Projected immunity against serotypes 1 and 2 in April 2016 in Pakistan for five alternative scenarios of supplementary campaigns. (A) Maps describing the SIAs between July 2015 and March 2016 for the alternative scenarios. (B) Projected immunity against serotypes 1 and 2 in April 2016 for the scenarios described in (A). (C) Same as (B) assuming improvements in access in Karachi, Peshawar and FATA (North and South Waziristan, Kurram and Khyber), as detailed in Figure XXIII. The publication of this map does not imply the expression of any opinion whatsoever on the part of WHO concerning the legal status of any territory, city or area or of its authorities, or concerning the delimitation of its frontiers or boundaries.

A

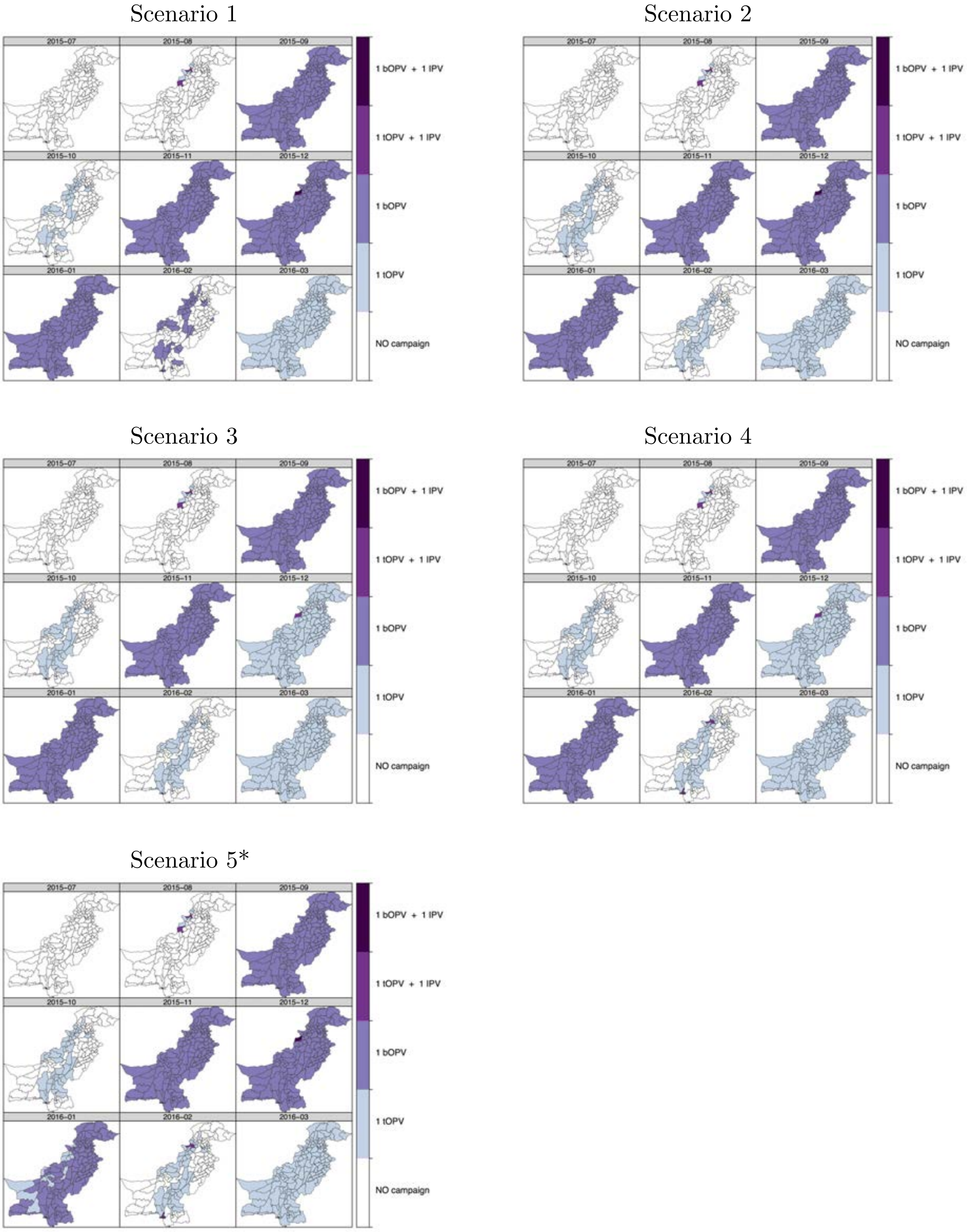

*In January 2016, districts receiving tOPV were also included in the national bOPV SIA.

B

Serotype 1 | Serotype 2

Scenario 1

Scenario 2

Scenario 3

Scenario 4

Scenario 5

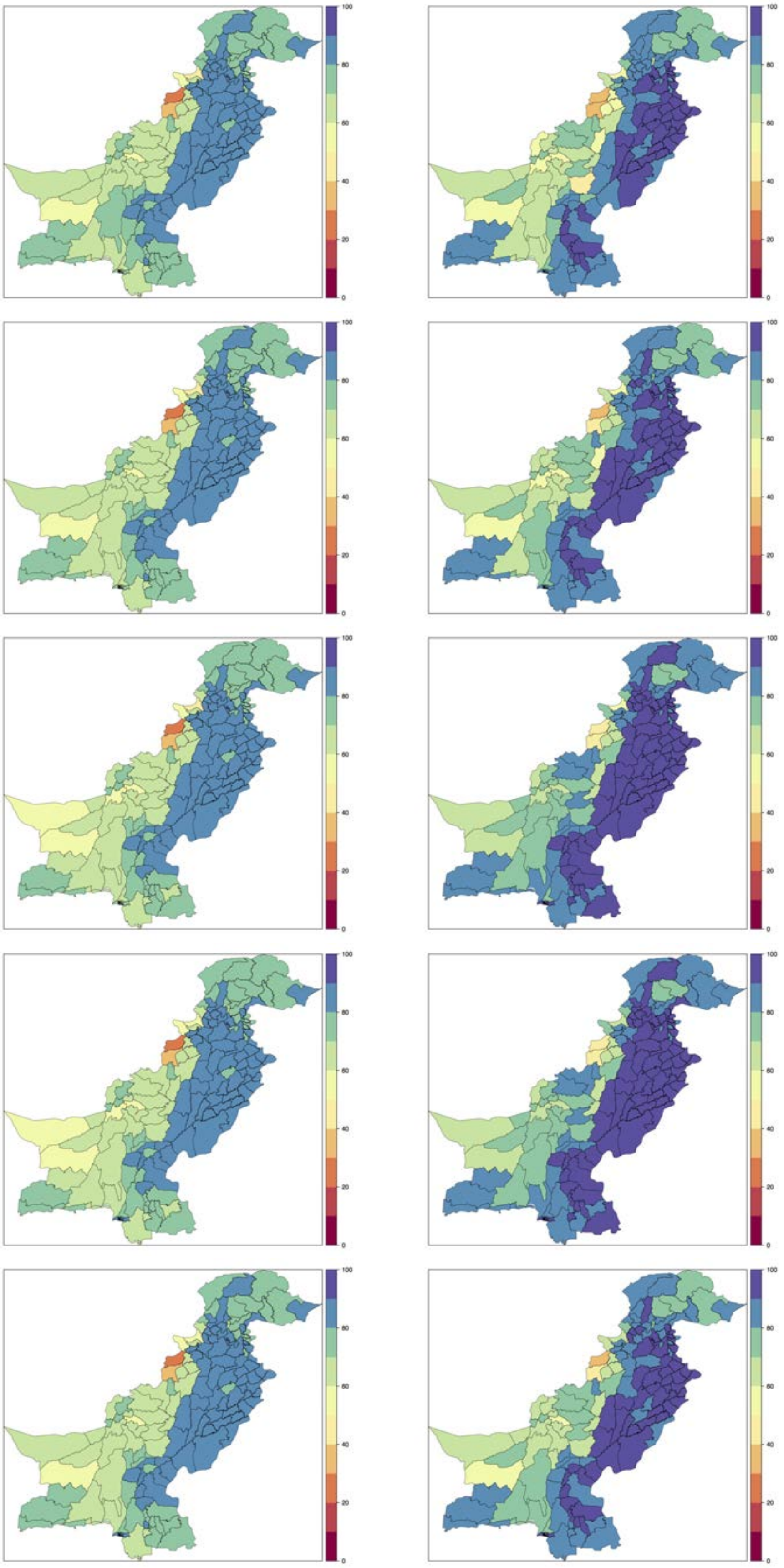

C

Serotype 1 | Serotype 2

Scenario 1

Scenario 2

Scenario 3

Scenario 4

Scenario 5

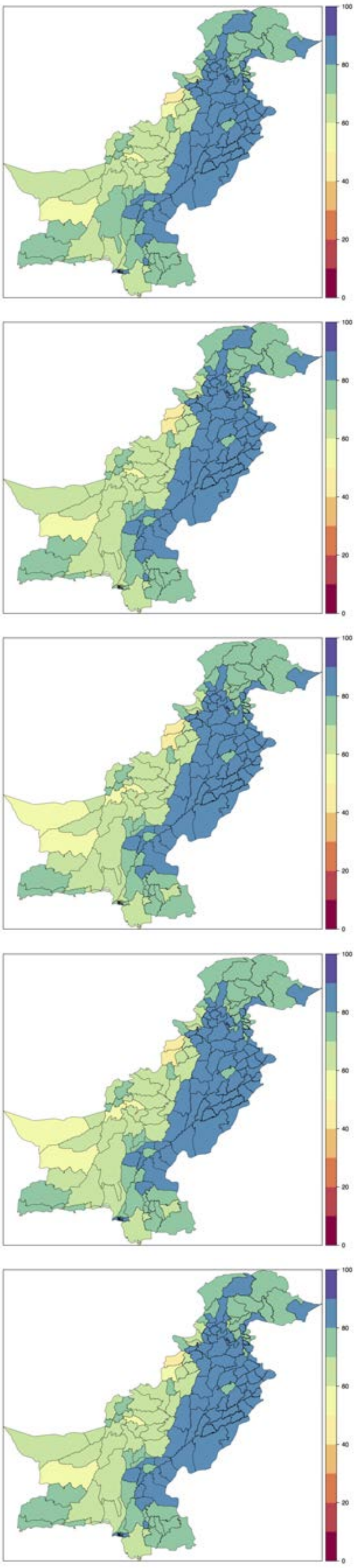

Figure XXXI: Estimated serotype-1 (left) and serotype-3 (right) population immunity in children <36 months of age Jan-Jun 2004 to Jan-Jun 2015 in Nigeria. Bottom: Estimates for the whole country weighted by population size. The publication of this map does not imply the expression of any opinion whatsoever on the part of WHO concerning the legal status of any territory, city or area or of its authorities, or concerning the delimitation of its frontiers or boundaries.

Serotype-1 population immunity

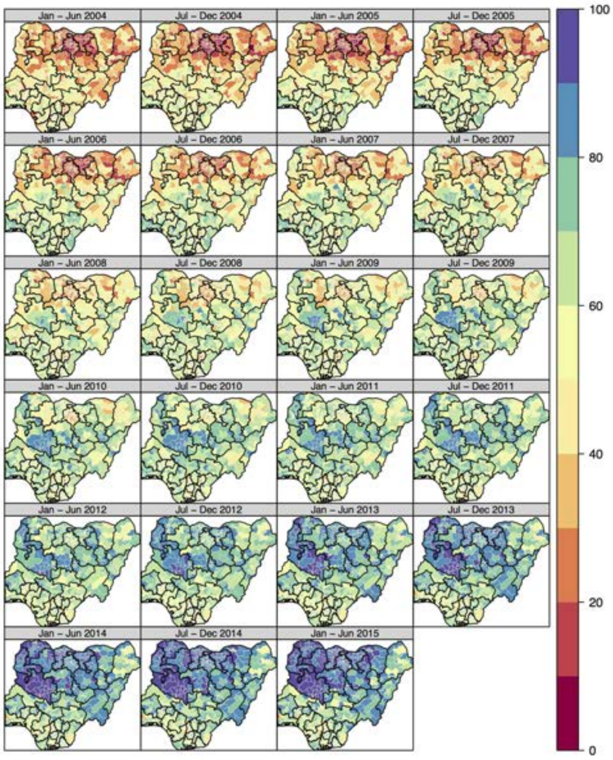

Serotype-3 population immunity

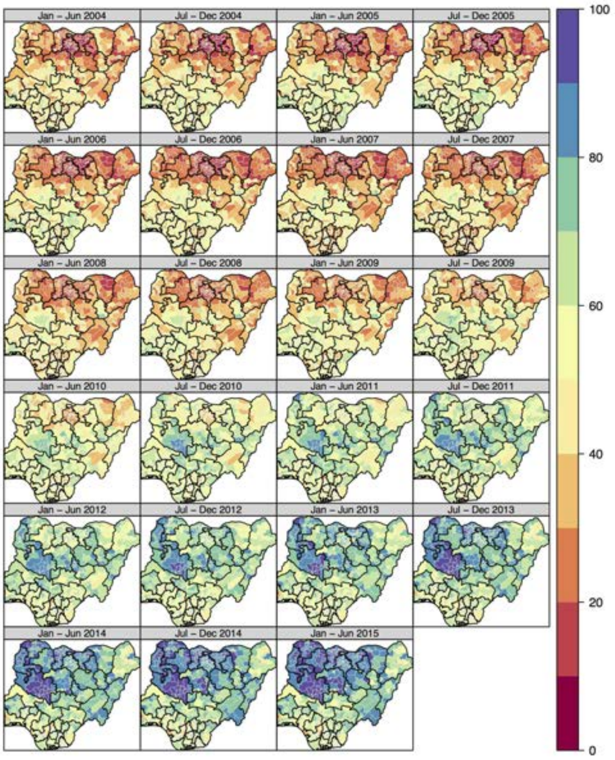

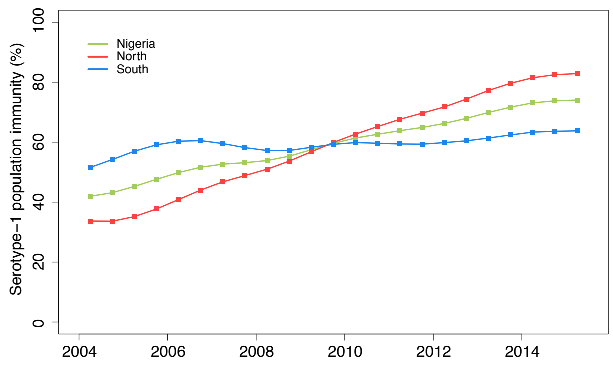

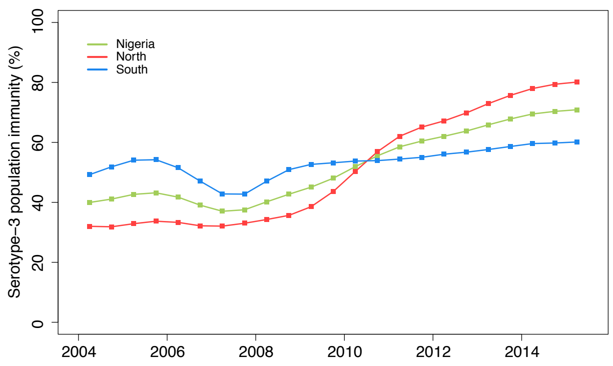

Table I: District groupings used in the estimation of SIA coverage in Pakistan.

Region	District grouping	Districts within the grouping
Balochistan	North	Barkhan, Bolan, Dera Bugti, Harnai, Kohlu, Killa Saifullah, Loralai, Mastung, Musakhel, Sibi, Zhob
	Quetta	Kabdulah, Pishin, Quetta, Ziarat
	South	Awaran, Chaghai, Gwadur, Jaffarabad, Jhal Magsi, Kalat, Kech, Kharan, Khuzdar, Lasbela, Noshki, Nasirabad, Panjgour, Washuk
FATA and south KPK	Wazir	Wazir North, Wazir South
	Khyber	Khyber, Kurram, Orakzai
	Mohmand	Mohmand, Bajour
	South KPK	Bannu, Dera Ismail Khan, Lakki Marwat, Tank
KPK	North	Abbottabad, Batagram, Bunir, Chitral, Dir Lower, Dir Upper, Haripur, Kohistan, Malakand, Mansehra, Shangla, Swat, Torghar
	Central	Charsadda, Hangu, Karak, Kohat, Mardan, Nowshera, Peshawar, Swabi
Karachi+ (and neighbouring districts of Sindh)	Gadap	Gadap Town and Gulshan Iqbal
	Rest of Karachi	Badin, Dadu, Hyderabad, Jamshoro, Karachi (excluding Gadap Town and Gulshan Iqbal), Matiari, Thatta, Tando Muhammad Khan
Sindh	none	Ghotki, Jacobabad, Kambar, Kashmore, Khairpur, Larkana, Mirpurkhas, Naushahro Feroze, Sanghar, Shaheed Benazirabad, Shikarpur, Sukkur, Tando Allahyar, Tharparkar, Umarkot
East Punjab	none	Bahawalpur, Bahwlnagar, Chiniot, Faisalabad, Gujranwala, Hafizabad, Jhang, Kasur, Khanewal, Lahore, Lodhran, Mandi Bahauddin, Multan, Nankanasahib, Narowal, Okara, Pakpatten, Rahim Yar Khan, Sahiwal, Sheikhupura, Sialkot, Toba Tek Singh, Vehari
West Punjab (and Islamabad)	none	Attock, Bhakkar, Chakwal, Dera Ghazi Khan, Gujrat, Islamabad (CDA and ICT), Jhelum, Khushab, Layyah, Mianwali, Muzfargarh, Rajanpur, Rawalpindi, Sargodha

Table II: Parameter estimates (mean and 95% credible interval) for the spatio-temporal random-effects models of serotype-2 population immunity.

Parameters	Nigeria Mean (95% CI)	Pakistan Mean (95% CI)
Intercept, α	0.29 (0.16–0.44)	0.66 (0.55–0.78)
Precision Gaussian observations, $1/\sigma^2$	37.68 (37.67–37.70)	22.28 (21.98–22.66)
Precision district-time interaction, $1/\sigma^2_\gamma$	110.01 (109.97–113.26)	104.66 (102.75–739.87)
Precision state-time interaction, $1/\sigma^2_\delta$	35.49 (35.48–35.52)	46.39 (42.47–504976.56)
Precision general temporal trend, $1/\sigma^2_\omega$	–	37.73 (34.55–404710.45)
Precision spatially-unstructured effect, $1/\sigma^2_\mu$	–	1898.56 (168.05–6908.86)
Precision spatially-structured effect, $1/\sigma^2_\nu$	–	2044.77 (126.26–7099.03)

Parameters	Afghanistan Mean (95% CI)	Cameroon Mean (95% CI)	Chad
Intercept, α	0.93 (0.65–1.20)	-0.005 (-3353.68–3350.87)	0.33 (-0.36–1.03)
Precision Gaussian observations, $1/\sigma^2$	13.72 (13.57–13.87)	17.73 (14.38–20.73)	15.34 (14.18–16.36)
Precision district-time interaction, $1/\sigma^2_\gamma$	–	–	–
Precision state-time interaction, $1/\sigma^2_\delta$	49.57 (44.70–55.64)	4.36 (1.96–9.35)	31.39 (8.29–92.41)
Precision general temporal trend, $1/\sigma^2_\omega$	–	–	–
Precision spatially-unstructured effect, $1/\sigma^2_\mu$	–	–	–
Precision spatially-structured effect, $1/\sigma^2_\nu$	–	–	–

Parameters	DRC Mean (95% CI)	Niger Mean (95% CI)	South Sudan
Intercept, α	0.53 (0.29–0.77)	0.16 (-0.68–0.99)	0.54 (-0.18–1.26)
Precision Gaussian observations, $1/\sigma^2$	14.79 (13.94–15.67)	16.11 (13.56–18.92)	15.01 (12.85–17.06)
Precision district-time interaction, $1/\sigma^2_\gamma$	–	–	–
Precision state-time interaction, $1/\sigma^2_\delta$	258.71 (59.94–797.24)	19.92 (7.00–45.04)	565.28 (13.90–3702.75)
Precision general temporal trend, $1/\sigma^2_\omega$	–	–	–
Precision spatially-unstructured effect, $1/\sigma^2_\mu$	–	–	–
Precision spatially-structured effect, $1/\sigma^2_\nu$	–	–	–

Table III: Summary of the distribution of the absolute difference between the projected immunity assuming independence between RI and SIA, and the projected immunity under the extreme scenario described in Section C.1.7, for different areas of Nigeria and Pakistan.

Nigeria	Min	1st Q	Median	Mean	3rd Q	Max
North	0.21	0.60	1.39	2.70	3.29	15.27
South	0.58	9.14	11.26	10.53	13.02	16.97

Pakistan	Min	1st Q	Median	Mean	3rd Q	Max
Balochistan	2.01	3.78	5.26	6.25	8.06	13.50
FATA	2.36	6.27	11.29	11.44	13.90	26.09
KPK	2.38	7.22	9.26	9.79	12.91	20.06
Sindh	2.91	4.49	6.15	8.28	11.36	21.41

References

[1] Mangal, T. D. *et al.* Key issues in the persistence of poliomyelitis in Nigeria: a case-control study. *The Lancet Global Health* **2**, e90–e97 (2014).

[2] Grassly, N. C. *et al.* New strategies for the elimination of polio from India. *Science* **314**, 1150–1153 (2006).

[3] Pons-Salort, M. *et al.* Preventing vaccine-derived poliovirus emergence during the polio endgame. *PLoS Pathogens* **12**, 1–18 (2016).

[4] Besag, J., York, J. & Mollie, A. Bayesian Image-Restoration, with 2 Applications in Spatial Statistics. *Annals of the Institute of Statistical Mathematics* **43**, 1–20 (1991).

[5] Knorr-Held, L. Bayesian modelling of inseparable space-time variation in disease risk. *Statistics in Medicine* **19**, 2555–2567 (2000).

[6] Upfill-Brown, A. M. *et al.* Predictive spatial risk model of poliovirus to aid prioritization and hasten eradication in Nigeria. *BMC Medicine* **12**, 1 (2014).

[7] Rue, H., Martino, S. & Chopin, N. Approximate Bayesian inference for latent Gaussian models by using integrated nested Laplace approximations. *Journal of the Royal Statistical Society: Series B* **71**, 319–392 (2009).

[8] R-INLA. URL www.r-inla.org.

[9] Grassly, N. C. Immunogenicity and Effectiveness of Routine Immunization With 1 or 2 Doses of Inactivated Poliovirus Vaccine: Systematic Review and Meta-analysis. *Journal of Infectious Diseases* **210**, S439–S446 (2014).

[10] UNICEF, Multiple Indicator Cluster Survey (MICS). URL www.unicef.org/statistics/index_24302.html.

[11] O'Reilly, K. M. *et al.* The effect of mass immunisation campaigns and new oral poliovirus vaccines on the incidence of poliomyelitis in Pakistan and Afghanistan, 2001–11: a retrospective analysis. *Lancet* **380**, 491–498 (2012).

[12] O'Reilly, K. M. *et al.* A new method to estimate the coverage of mass vaccination campaigns against poliomyelitis from surveillance data. *American Journal of Epidemiology* **182**, 961–970 (2015).

[13] Carnell, R. *lhs: Latin Hypercube Samples* (2016). URL http://CRAN.R-project.org/package=lhs. R package version 0.13.
