# Supplementary material for: Population Immunity against Serotype-2 Poliomyelitis Leading up to the Global Withdrawal of the Oral Poliovirus Vaccine: Spatio-temporal Modelling of Surveillance Data
Source: PLoS Med. 2016 Oct 4;13(10):e1002140. doi: 10.1371/journal.pmed.1002140 (PMC5049753; doi:10.1371/journal.pmed.1002140)
Supplement: S2 Text — (PDF) [file pmed.1002140.s002.pdf]

S2 Text

Crude and estimated serotype-2 population immunity in Nigeria by district and 6-month period from Jan-Jun 2004 to Jan-Jun 2015

Figure XXXII. Crude (black circles) and estimated (red points) serotype-2 population immunity in each district of Nigeria and 6-month period from Jan-Jun 2004 to Jan-Jun 2015 presented by state in alphabetical order. The size of the black circles indicates the number of non-polio AFP cases that informed that estimate.

ABIA

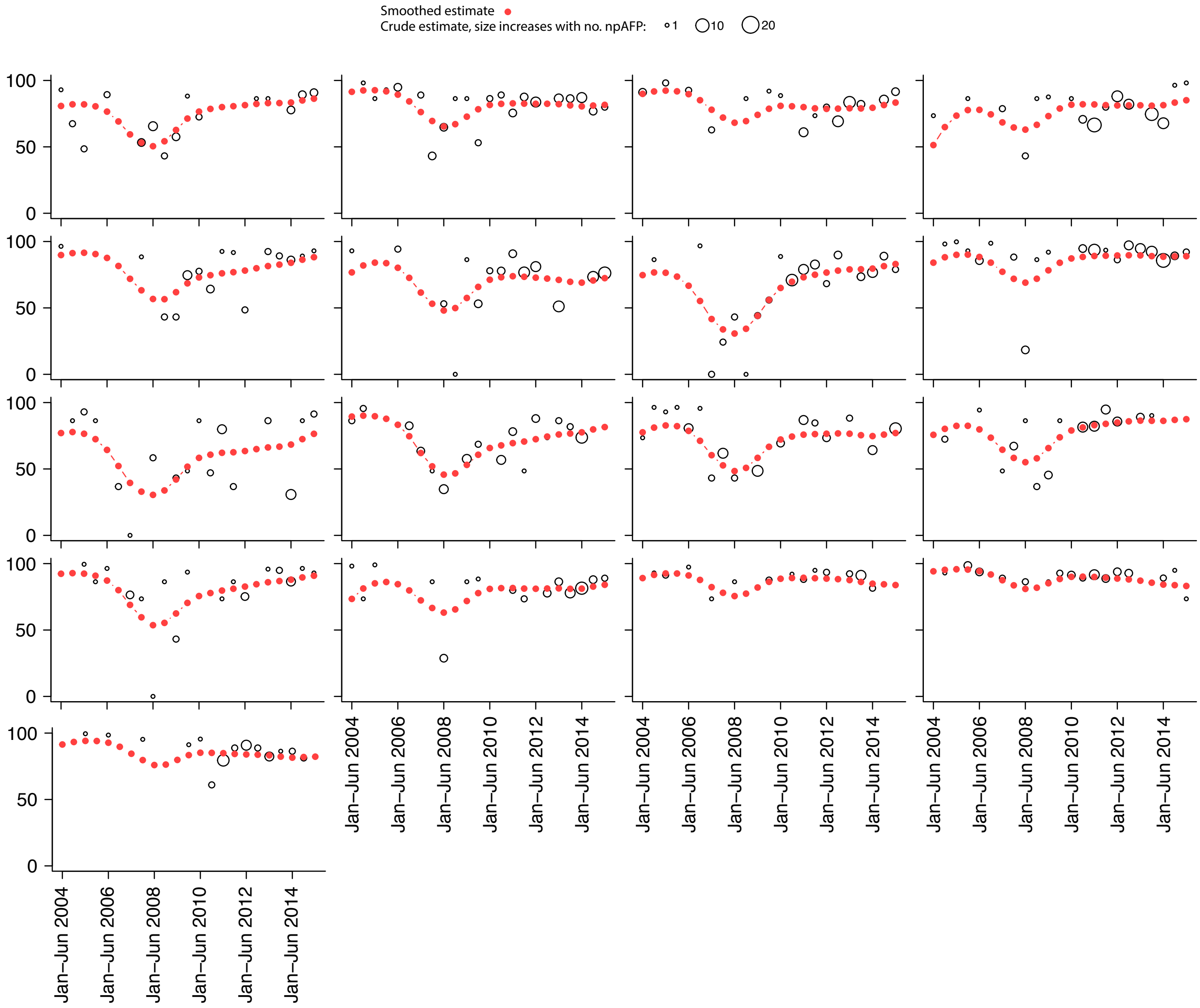

ADAMAWA

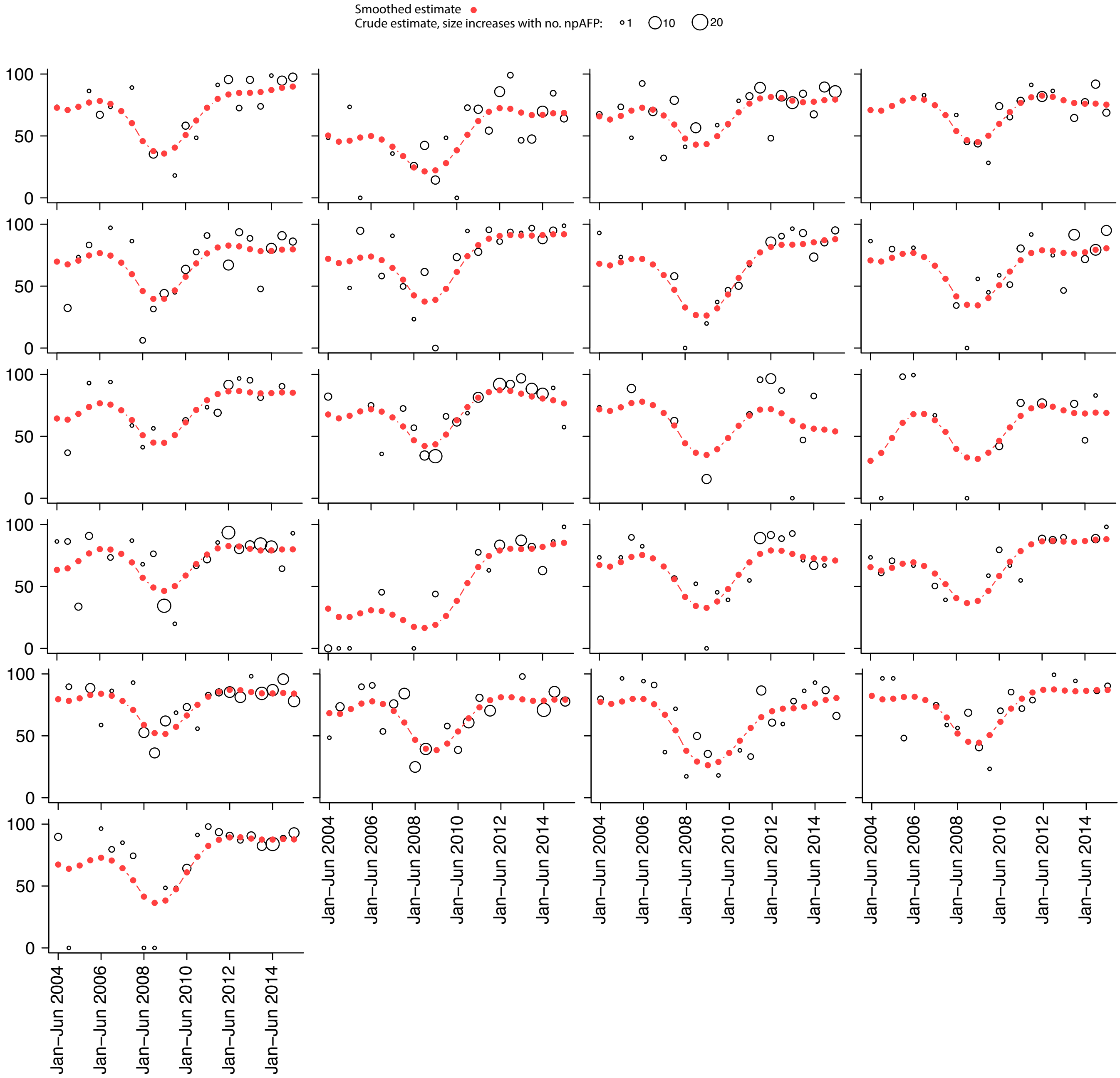
Smoothed estimate
Crude estimate, size increases with no. npAFP:
1
10
20
100
50
0
Jan–Jun 2004
Jan–Jun 2006
Jan–Jun 2008
Jan–Jun 2010
Jan–Jun 2012
Jan–Jun 2014

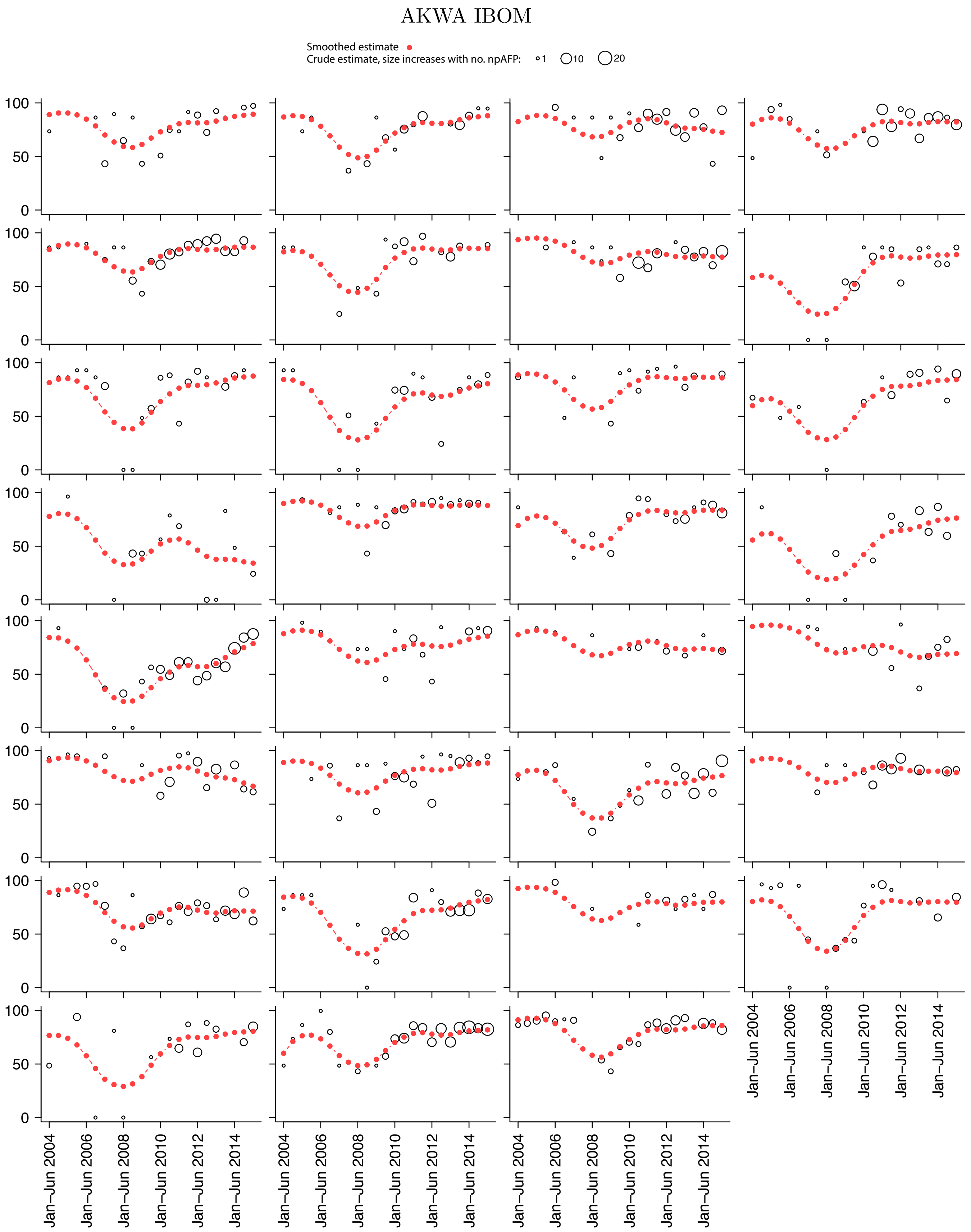
AKWA IBOM
Smoothed estimate
Crude estimate, size increases with no. npAFP:
1
10
20
100
50
0
Jan–Jun 2004
Jan–Jun 2006
Jan–Jun 2008
Jan–Jun 2010
Jan–Jun 2012
Jan–Jun 2014

ANAMBRA

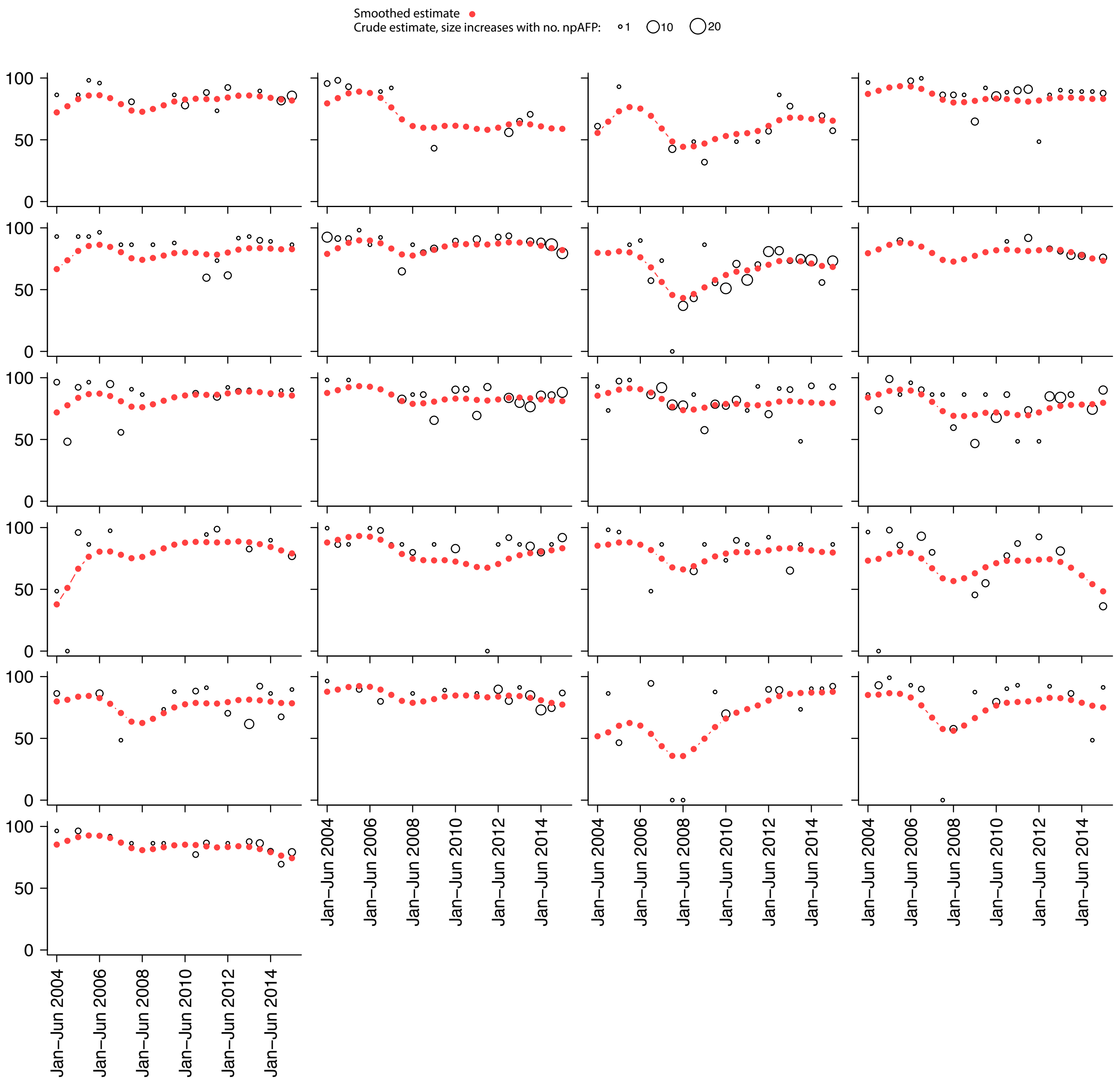

Smoothed estimate
Crude estimate, size increases with no. npAFP:
1
10
20
100
50
0
Jan–Jun 2004
Jan–Jun 2006
Jan–Jun 2008
Jan–Jun 2010
Jan–Jun 2012
Jan–Jun 2014

BAUCHI

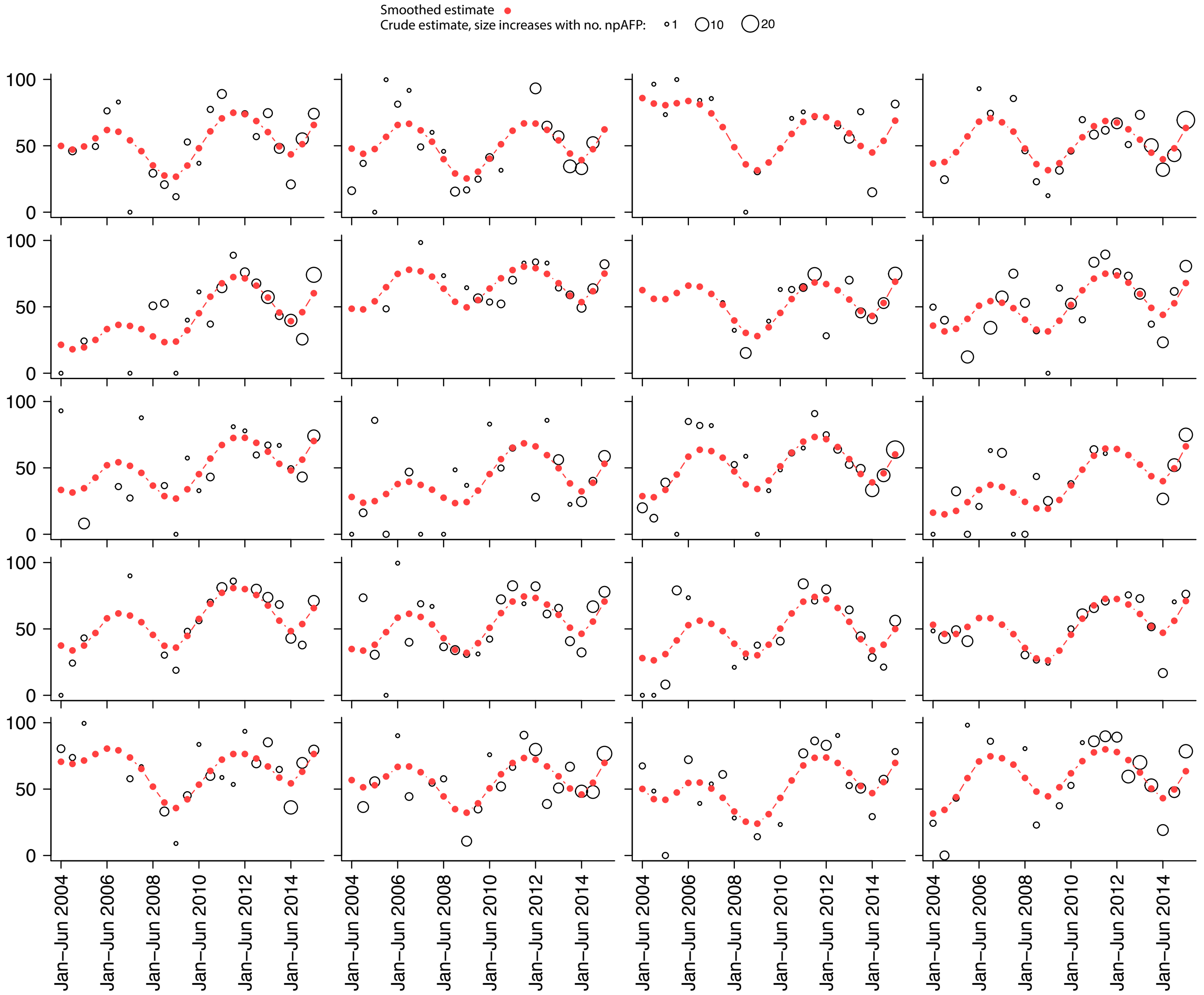

Smoothed estimate
Crude estimate, size increases with no. npAFP:
1
10
20
100
50
0
Jan–Jun 2004
Jan–Jun 2006
Jan–Jun 2008
Jan–Jun 2010
Jan–Jun 2012
Jan–Jun 2014

BAYELSA

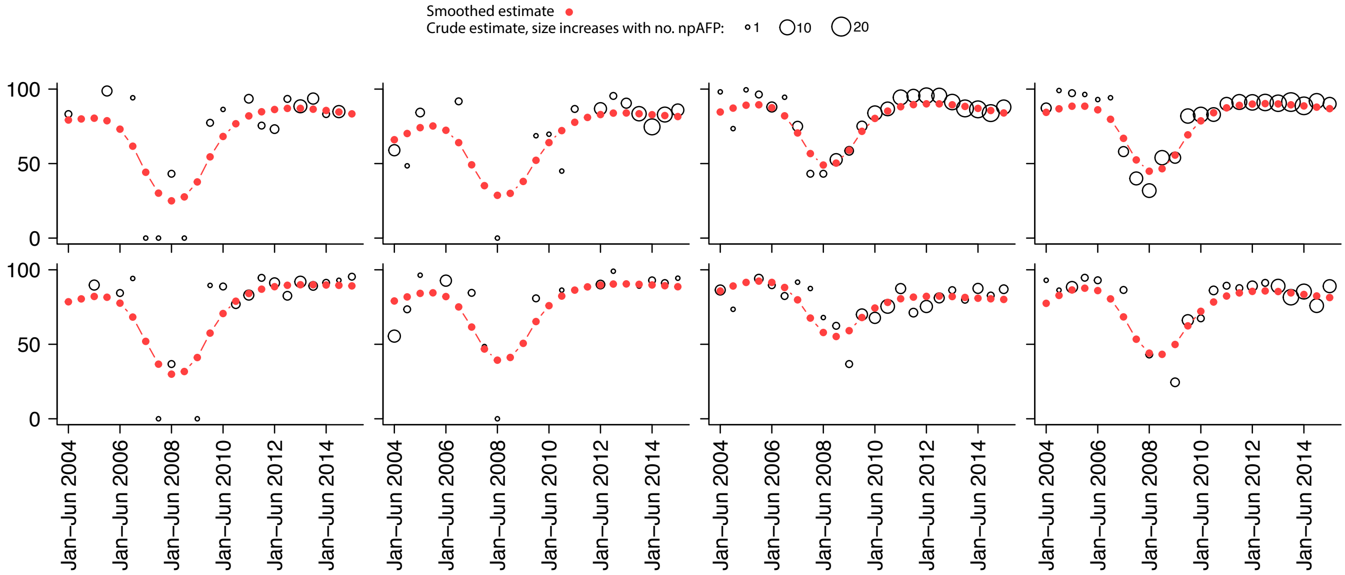

Smoothed estimate
Crude estimate, size increases with no. npAFP:
1
10
20
100
50
0
Jan–Jun 2004
Jan–Jun 2006
Jan–Jun 2008
Jan–Jun 2010
Jan–Jun 2012
Jan–Jun 2014

BENUE

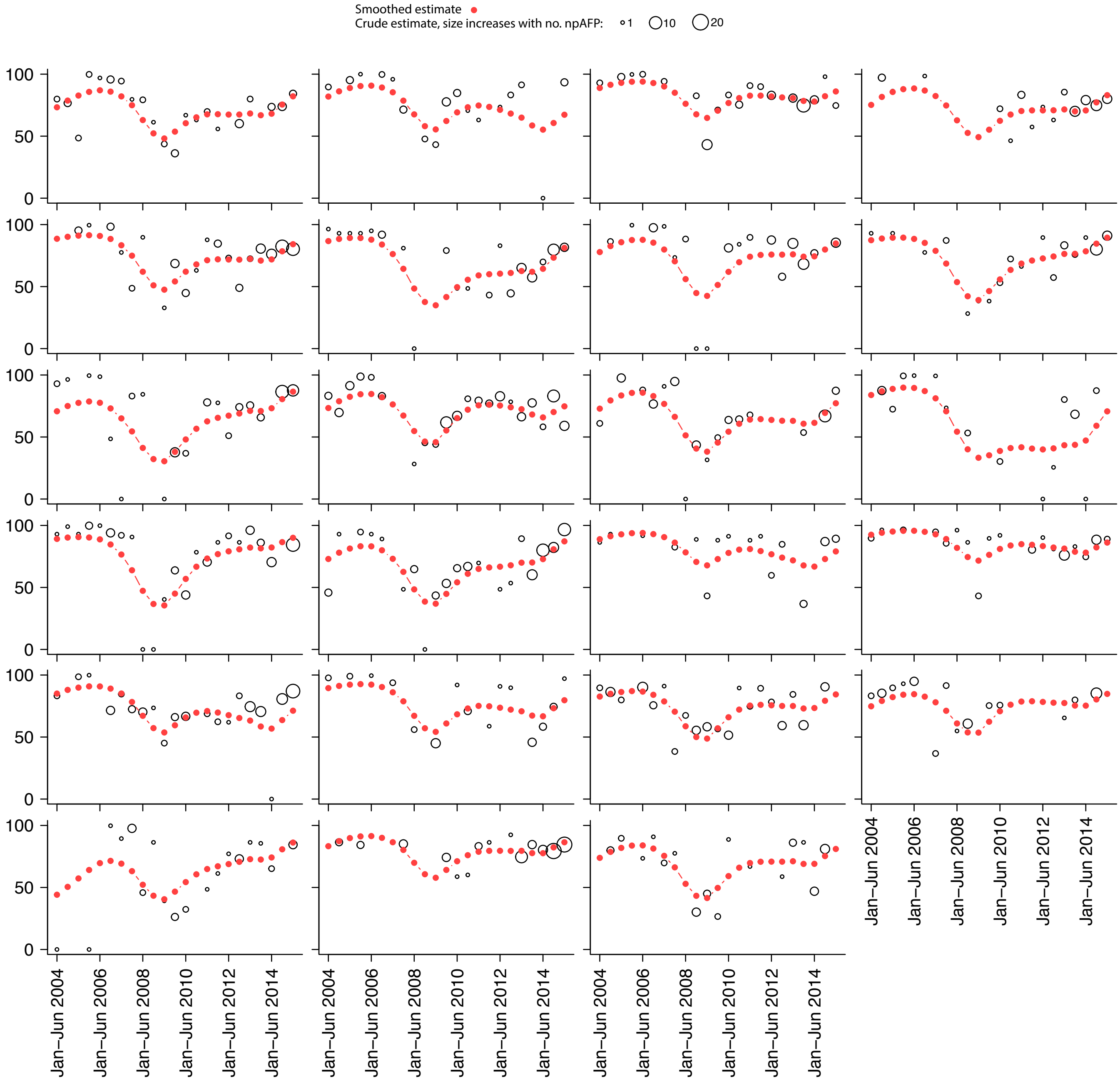

Smoothed estimate
Crude estimate, size increases with no. npAFP: 1 10 20
100
50
0
Jan–Jun 2004
Jan–Jun 2006
Jan–Jun 2008
Jan–Jun 2010
Jan–Jun 2012
Jan–Jun 2014

BORNO

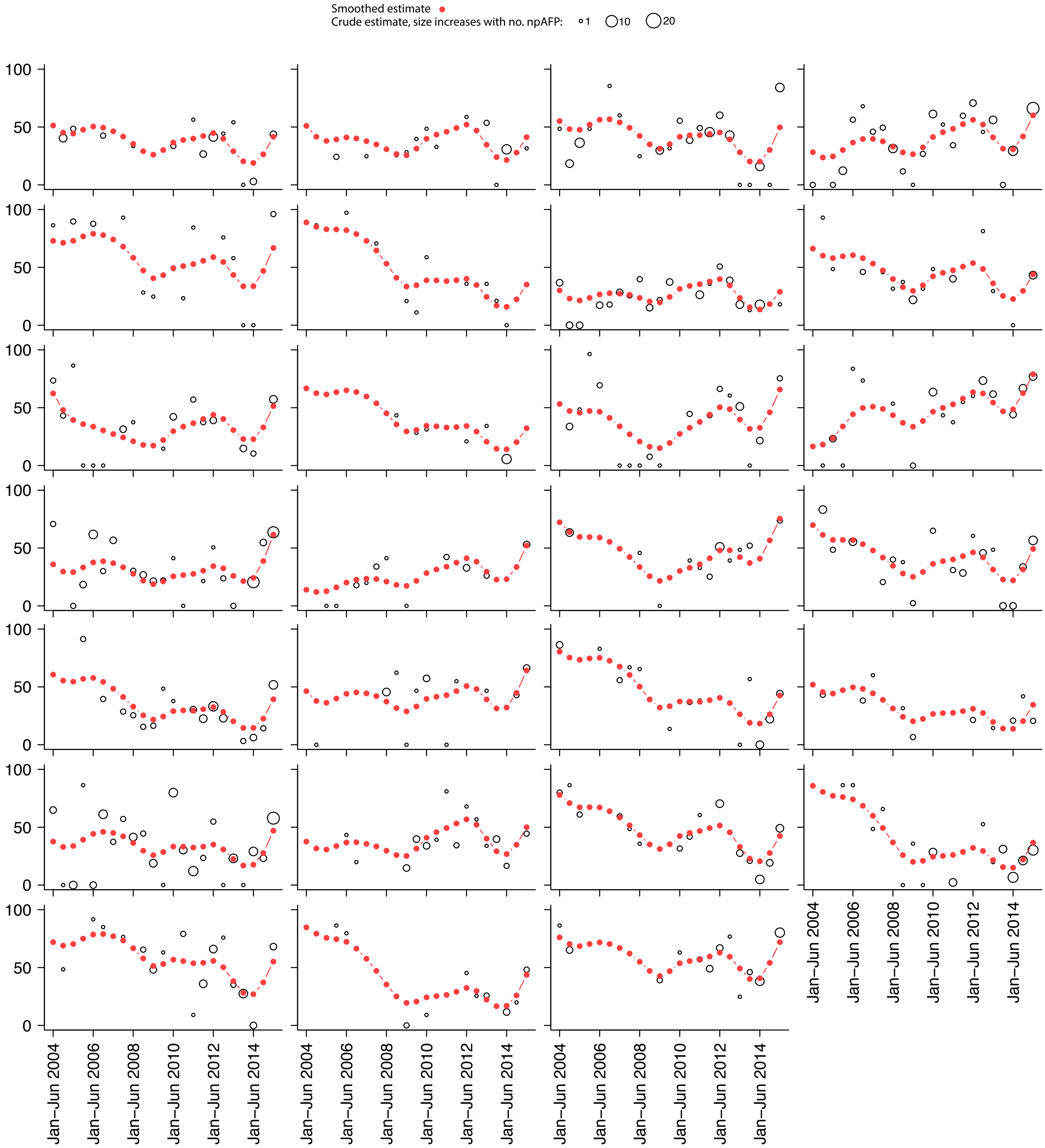

Smoothed estimate
Crude estimate, size increases with no. npAFP:
1
10
20
100
50
0
Jan–Jun 2004
Jan–Jun 2006
Jan–Jun 2008
Jan–Jun 2010
Jan–Jun 2012
Jan–Jun 2014

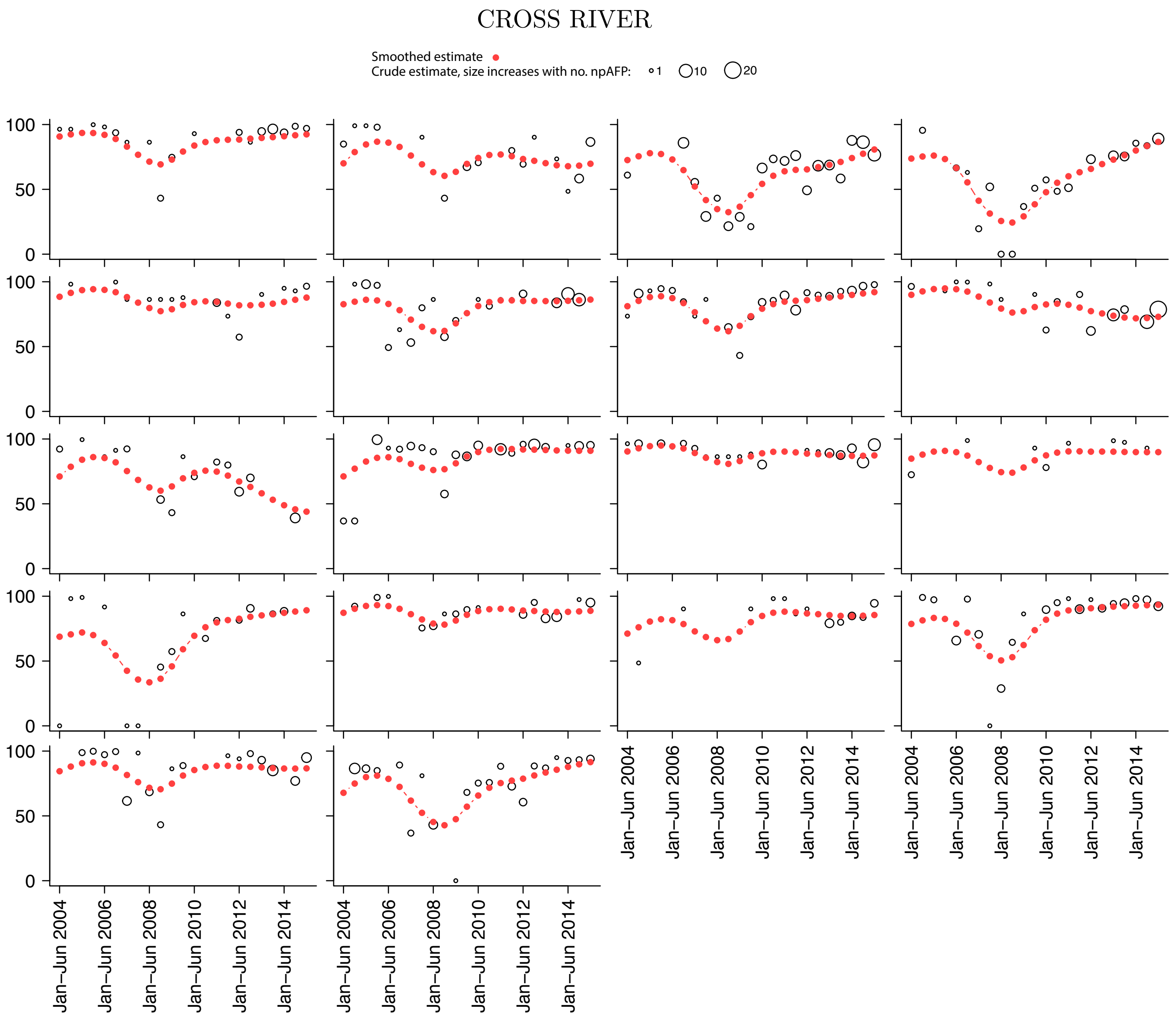

CROSS RIVER
Smoothed estimate
Crude estimate, size increases with no. npAFP:
1
10
20
100
50
0
Jan–Jun 2004
Jan–Jun 2006
Jan–Jun 2008
Jan–Jun 2010
Jan–Jun 2012
Jan–Jun 2014

DELTA

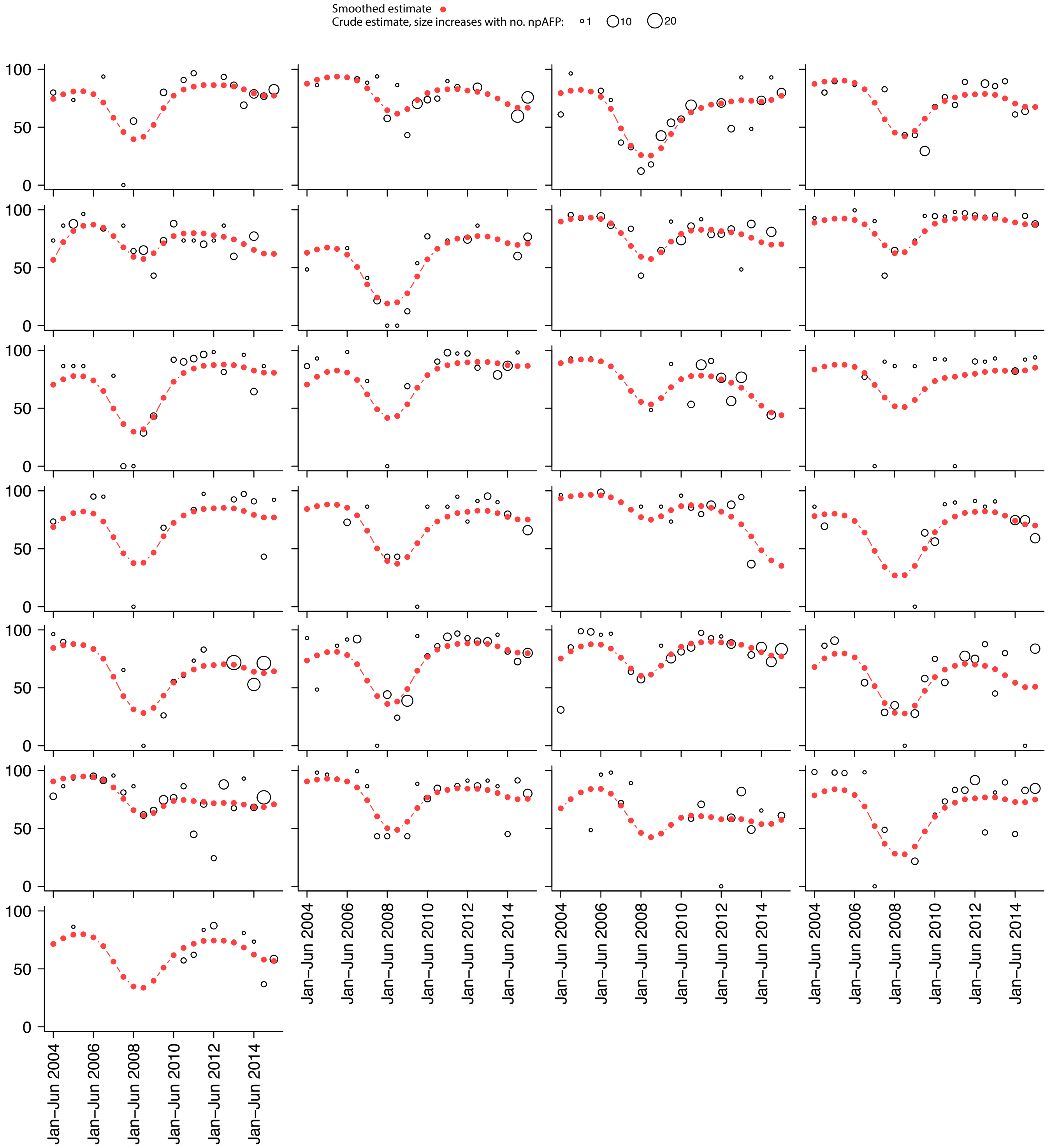

Smoothed estimate
Crude estimate, size increases with no. npAFP:
1
10
20
100
50
0
Jan–Jun 2004
Jan–Jun 2006
Jan–Jun 2008
Jan–Jun 2010
Jan–Jun 2012
Jan–Jun 2014

EBONYI

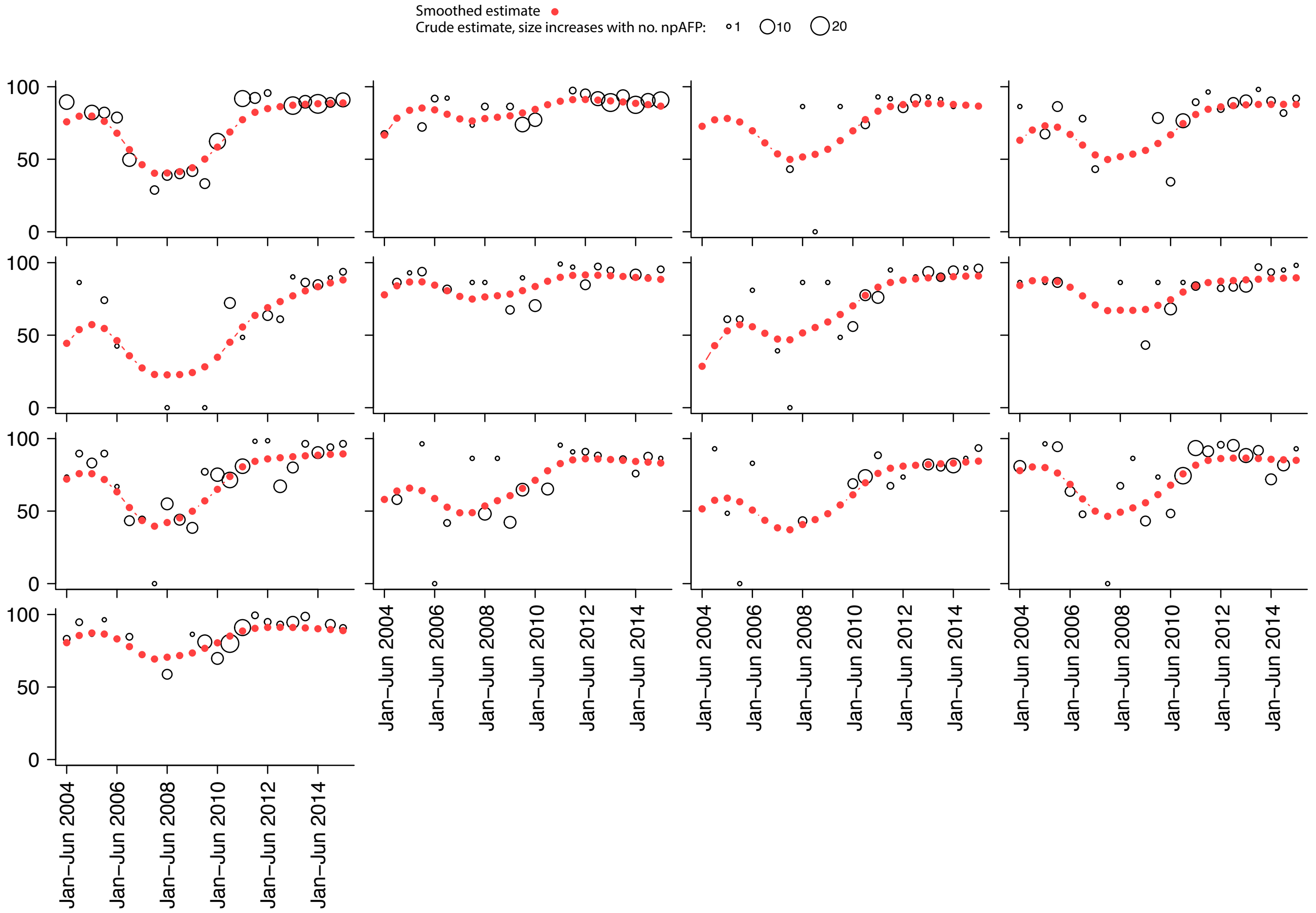

Smoothed estimate
Crude estimate, size increases with no. npAFP: 1 10 20
100
50
0
Jan–Jun 2004
Jan–Jun 2006
Jan–Jun 2008
Jan–Jun 2010
Jan–Jun 2012
Jan–Jun 2014

EDO

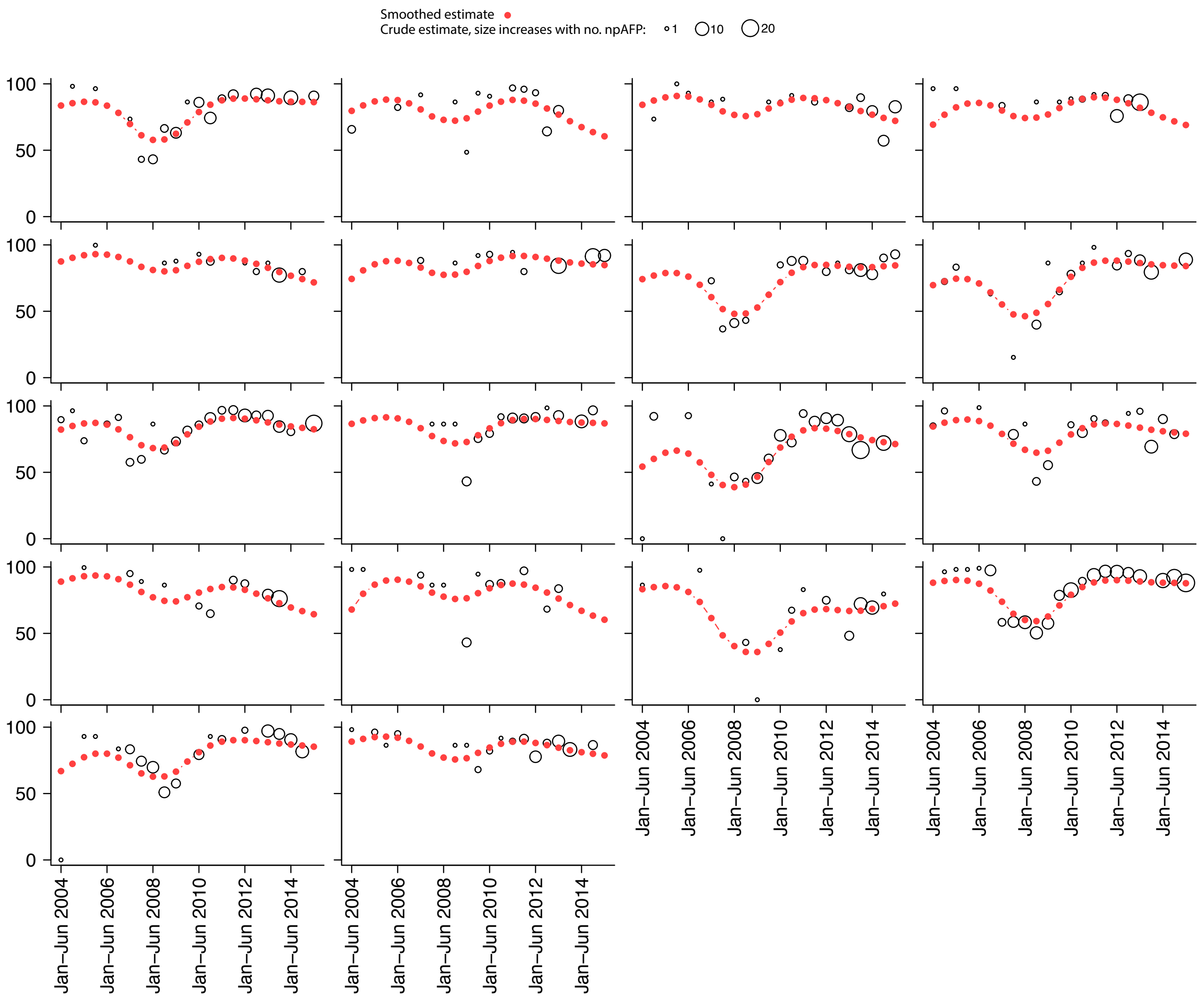

Smoothed estimate
Crude estimate, size increases with no. npAFP:
1
10
20
100
50
0
Jan–Jun 2004
Jan–Jun 2006
Jan–Jun 2008
Jan–Jun 2010
Jan–Jun 2012
Jan–Jun 2014

EKITI

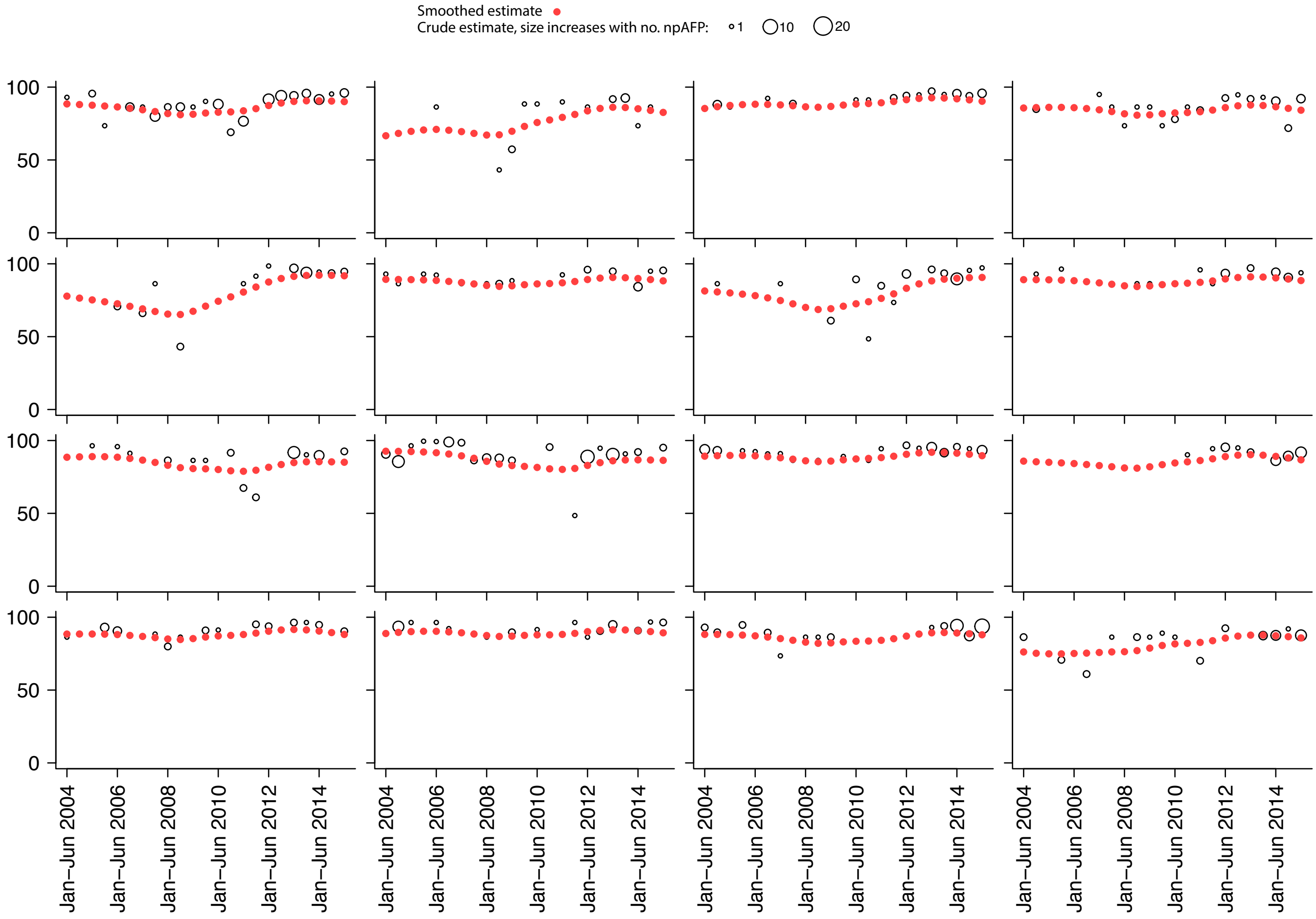

Smoothed estimate
Crude estimate, size increases with no. npAFP: 1 10 20
100
50
0
Jan–Jun 2004
Jan–Jun 2006
Jan–Jun 2008
Jan–Jun 2010
Jan–Jun 2012
Jan–Jun 2014

ENUGU

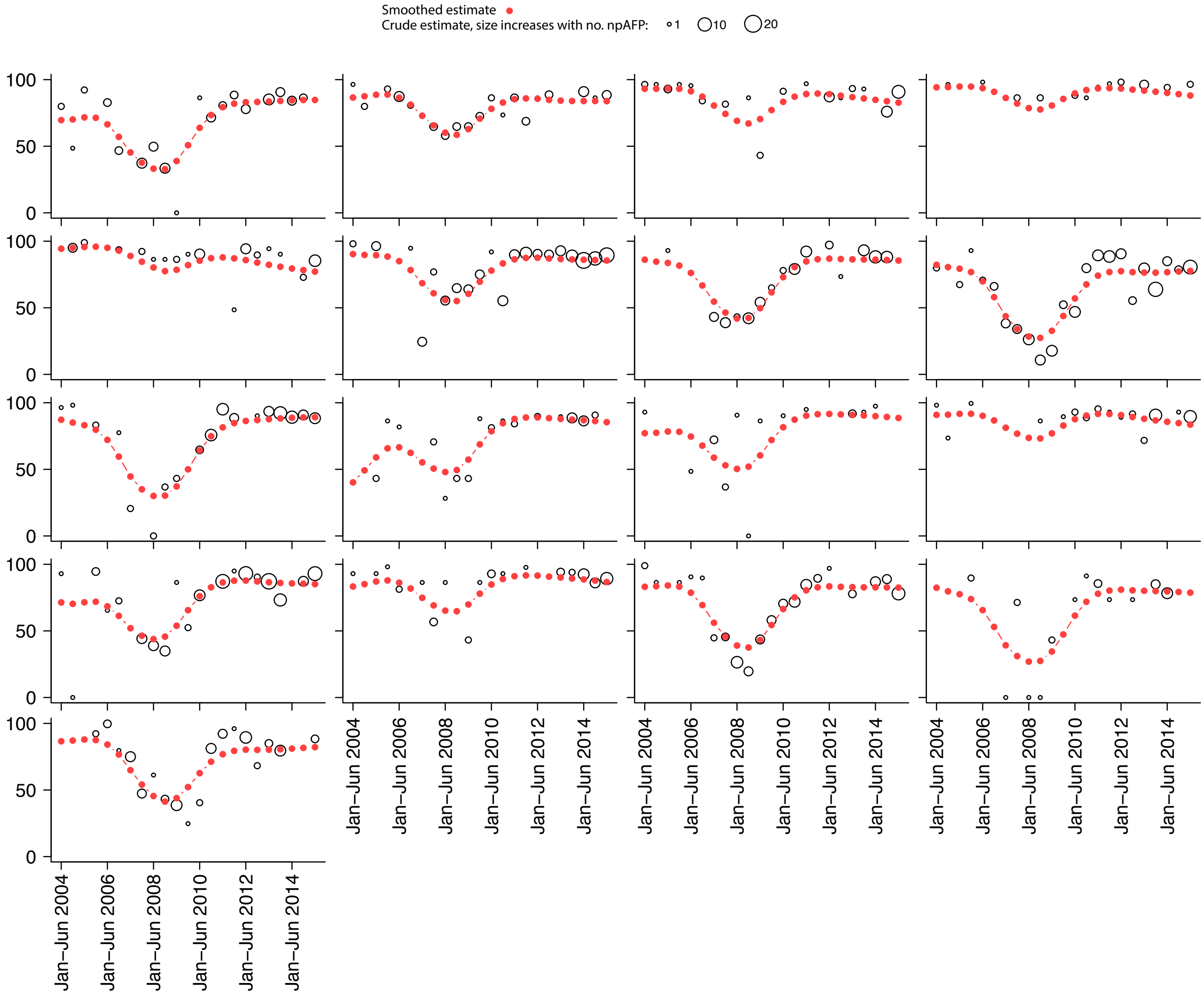

Smoothed estimate
Crude estimate, size increases with no. npAFP: 1 10 20
100
50
0
Jan–Jun 2004
Jan–Jun 2006
Jan–Jun 2008
Jan–Jun 2010
Jan–Jun 2012
Jan–Jun 2014

FCT ABUJA

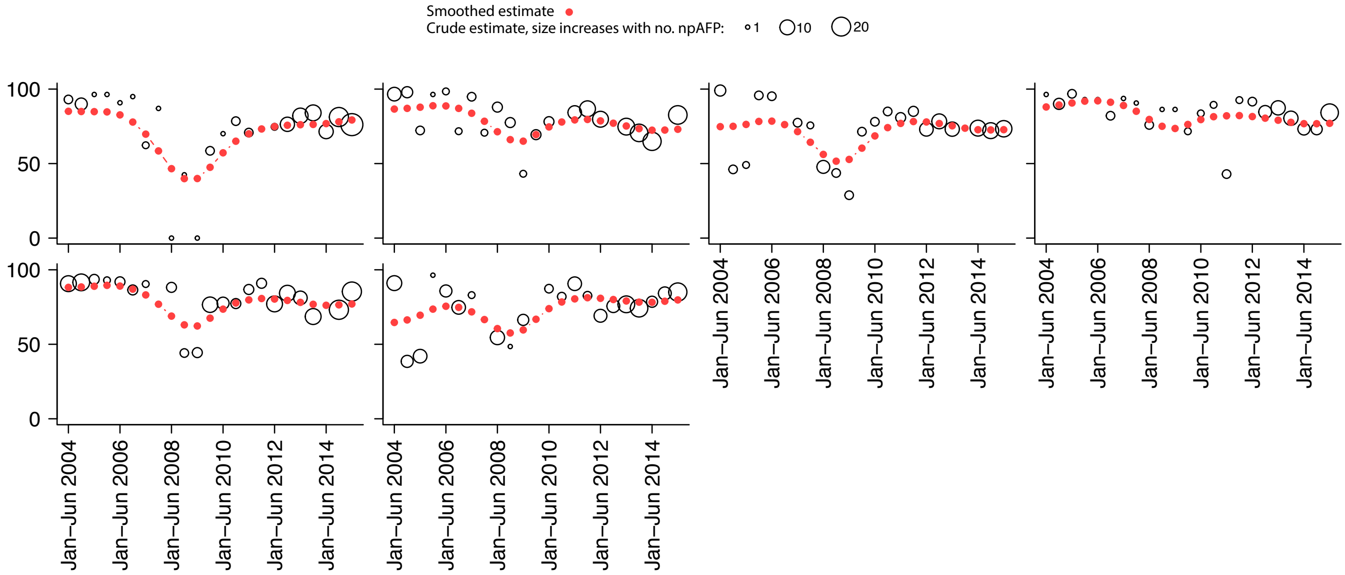

Smoothed estimate
Crude estimate, size increases with no. npAFP:
1
10
20
100
50
0
Jan–Jun 2004
Jan–Jun 2006
Jan–Jun 2008
Jan–Jun 2010
Jan–Jun 2012
Jan–Jun 2014

GOMBE

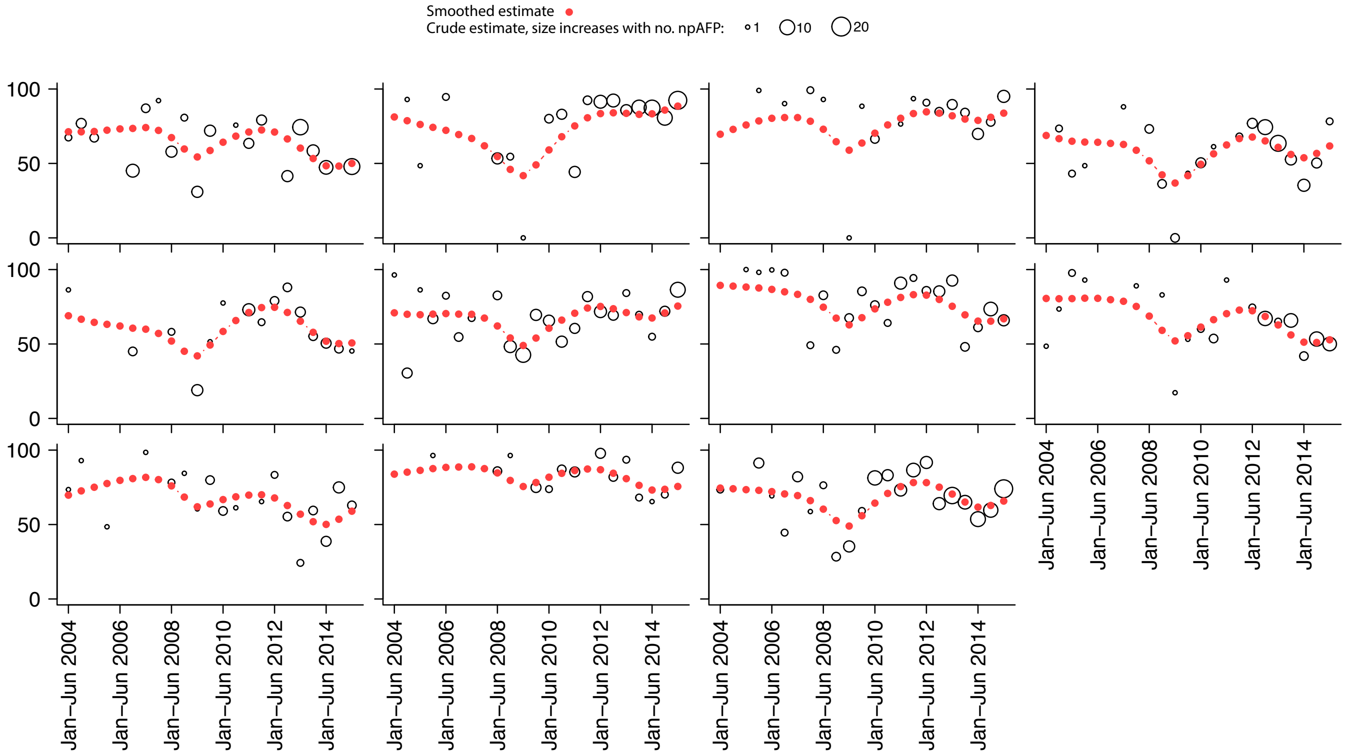
Smoothed estimate
Crude estimate, size increases with no. npAFP: 1 10 20
100
50
0
Jan–Jun 2004
Jan–Jun 2006
Jan–Jun 2008
Jan–Jun 2010
Jan–Jun 2012
Jan–Jun 2014

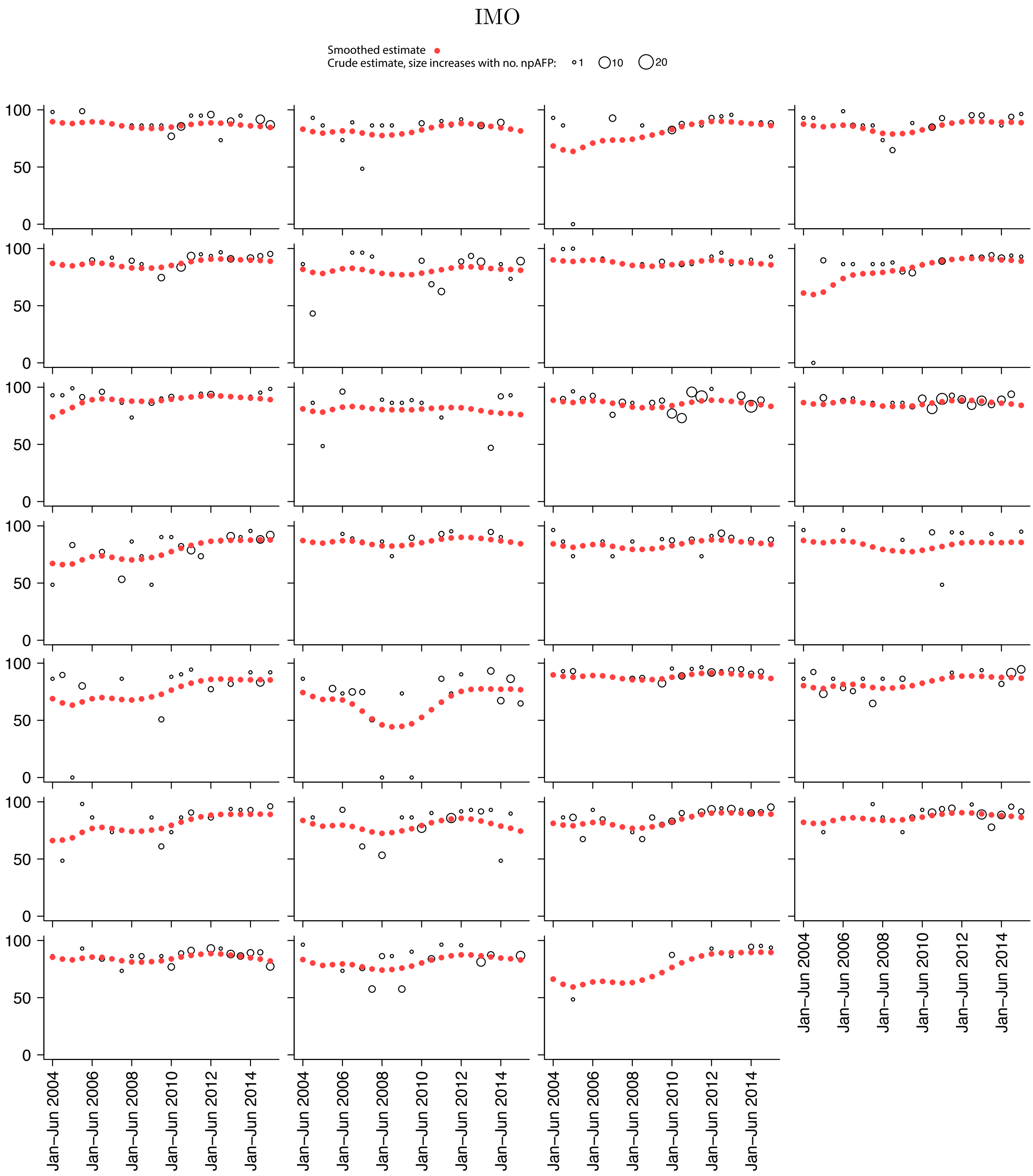
IMO
Smoothed estimate
Crude estimate, size increases with no. npAFP: 1 10 20
100
50
0
Jan–Jun 2004
Jan–Jun 2006
Jan–Jun 2008
Jan–Jun 2010
Jan–Jun 2012
Jan–Jun 2014

JIGAWA

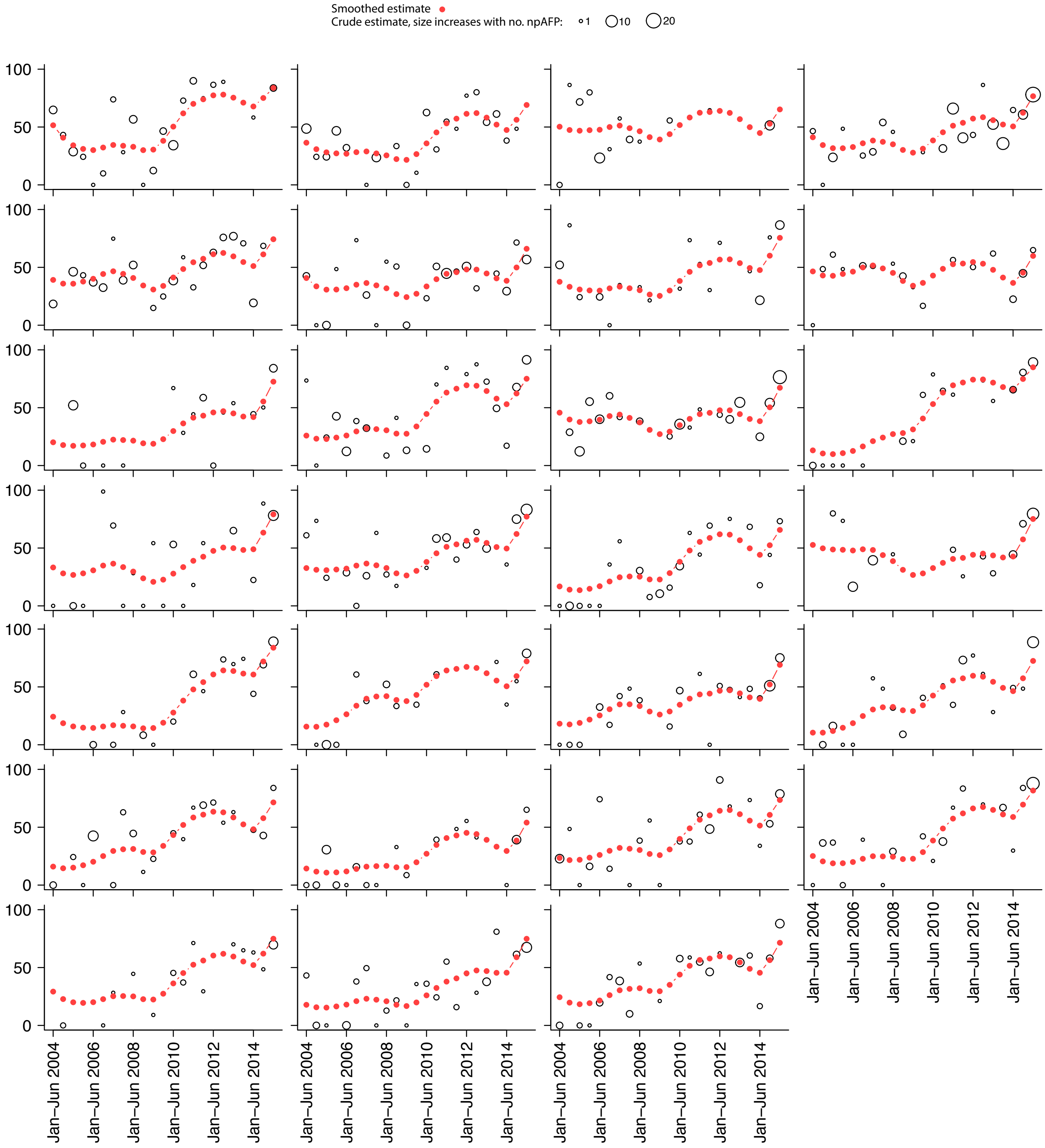

Smoothed estimate
Crude estimate, size increases with no. npAFP: 1 10 20
100
50
0
Jan–Jun 2004
Jan–Jun 2006
Jan–Jun 2008
Jan–Jun 2010
Jan–Jun 2012
Jan–Jun 2014

KADUNA

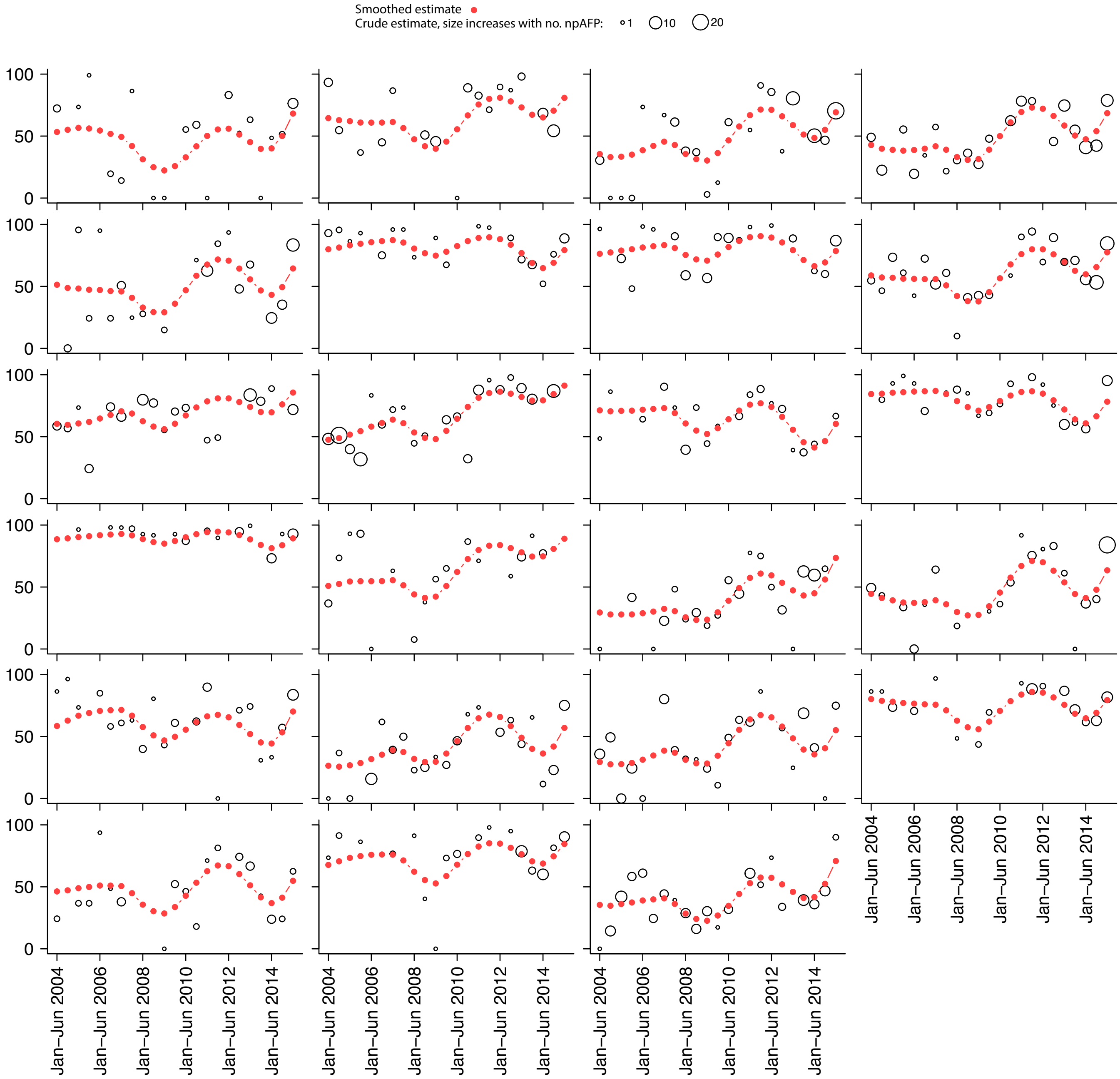
Smoothed estimate
Crude estimate, size increases with no. npAFP:
1
10
20
100
50
0
Jan–Jun 2004
Jan–Jun 2006
Jan–Jun 2008
Jan–Jun 2010
Jan–Jun 2012
Jan–Jun 2014

KANO (1/2)

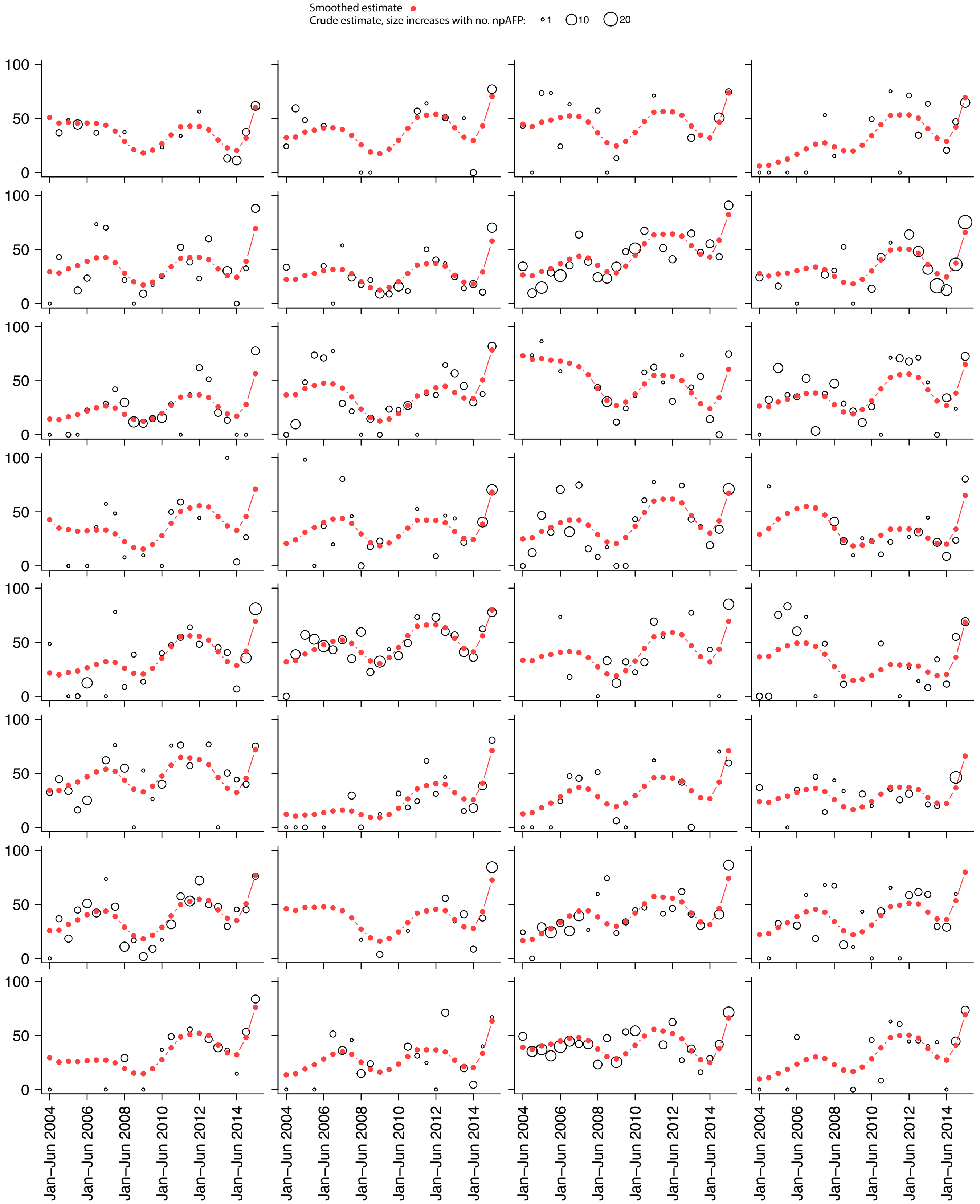

Smoothed estimate
Crude estimate, size increases with no. npAFP: 1 10 20
100
50
0
Jan–Jun 2004
Jan–Jun 2006
Jan–Jun 2008
Jan–Jun 2010
Jan–Jun 2012
Jan–Jun 2014

KANO (2/2)

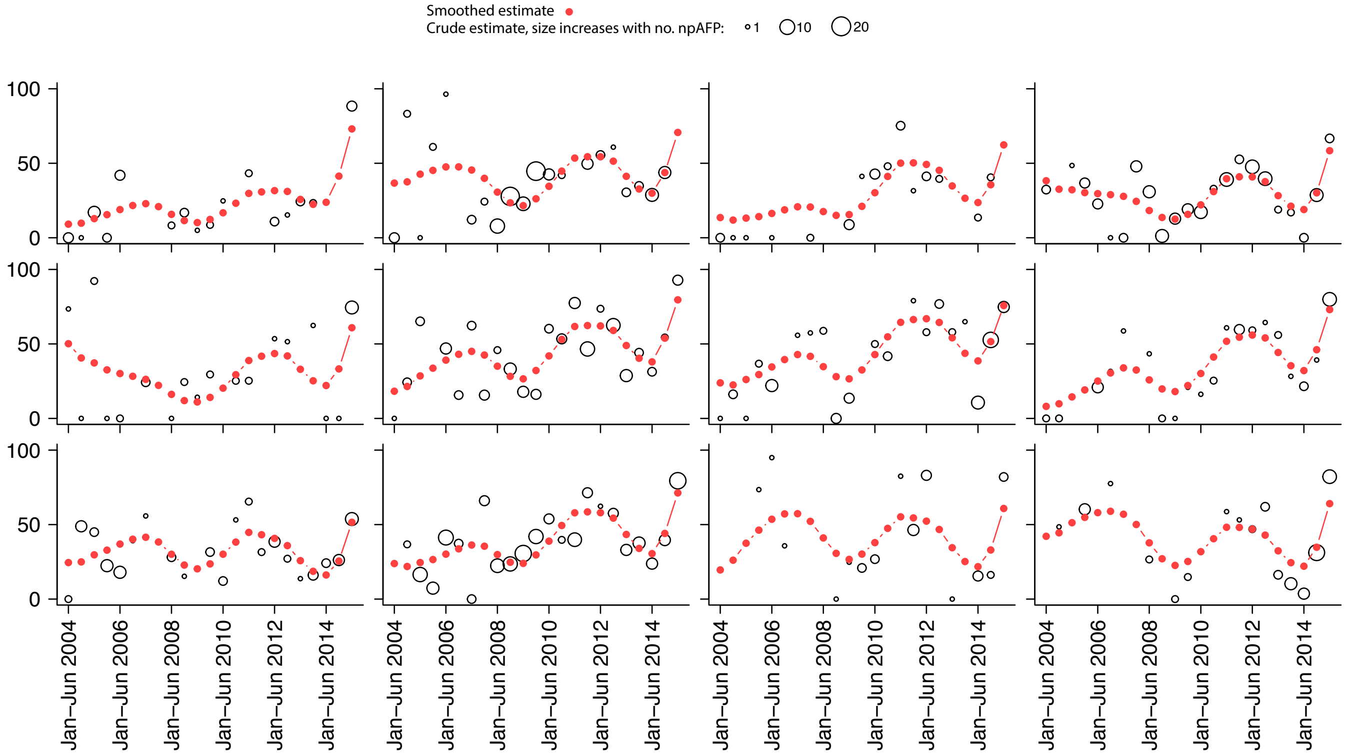

Smoothed estimate
Crude estimate, size increases with no. npAFP:
1
10
20
100
50
0
Jan–Jun 2004
Jan–Jun 2006
Jan–Jun 2008
Jan–Jun 2010
Jan–Jun 2012
Jan–Jun 2014

KATSINA

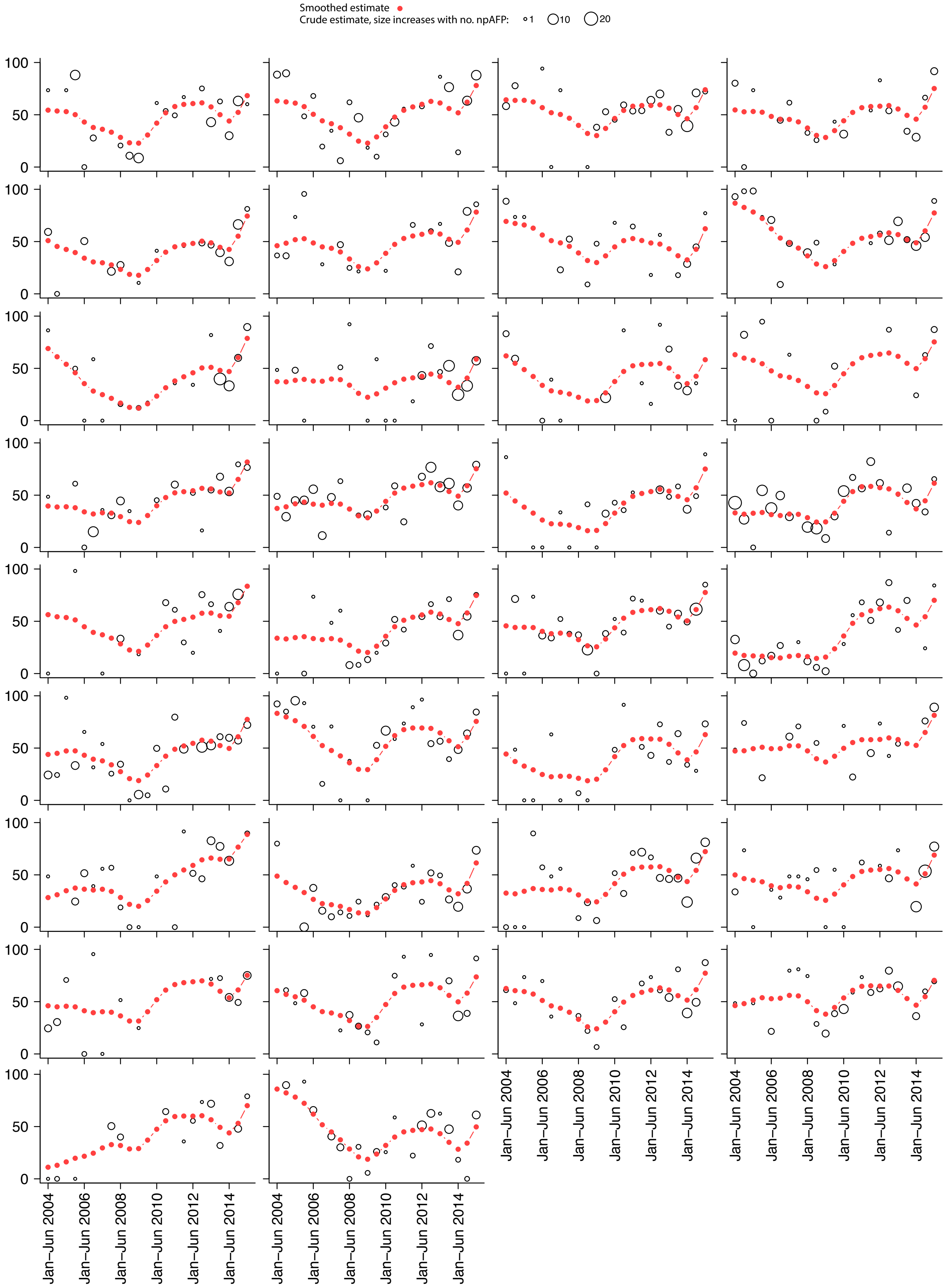
Smoothed estimate
Crude estimate, size increases with no. npAFP:
1
10
20
100
50
0
Jan–Jun 2004
Jan–Jun 2006
Jan–Jun 2008
Jan–Jun 2010
Jan–Jun 2012
Jan–Jun 2014

KEBBI

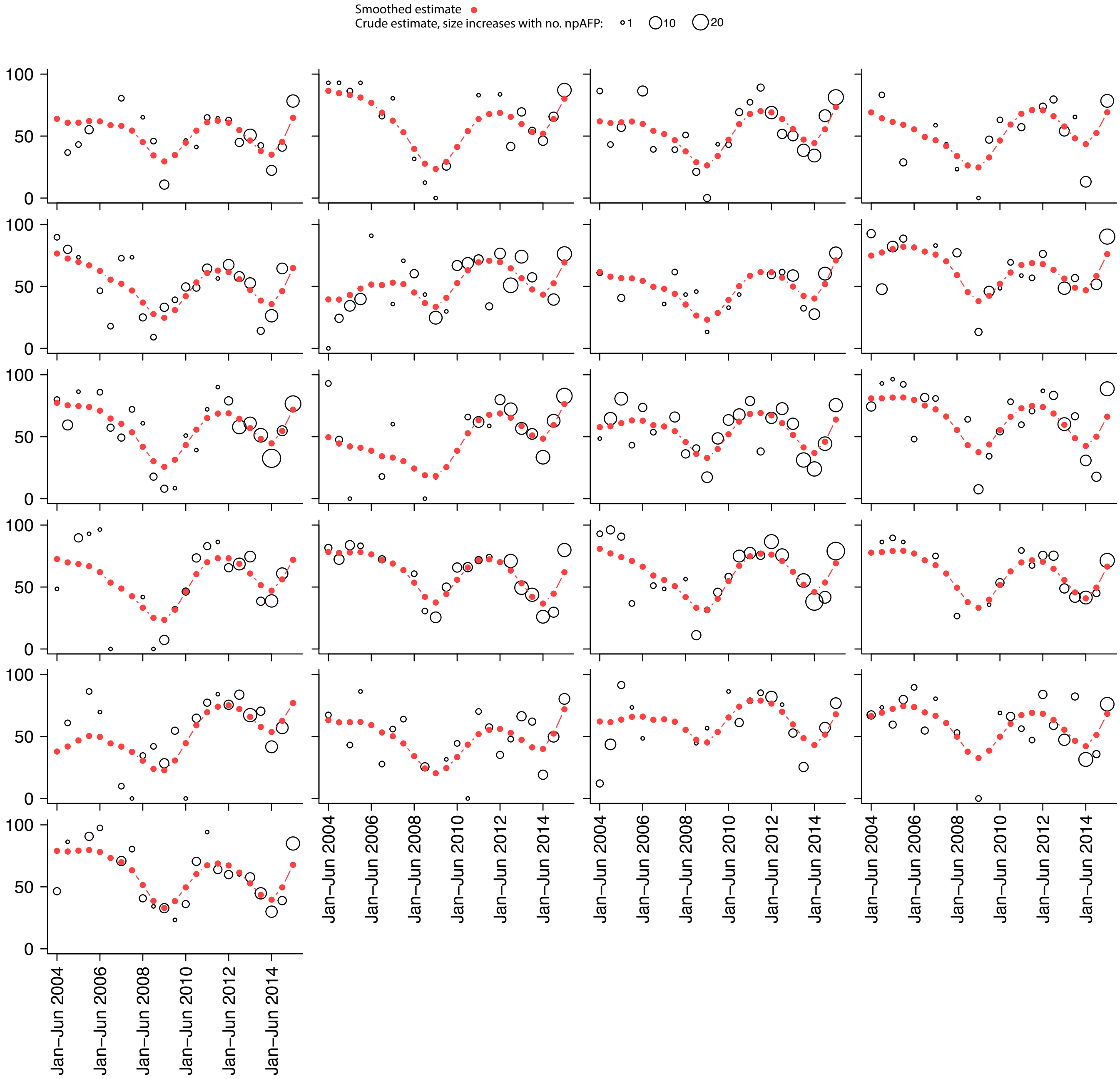

Smoothed estimate
Crude estimate, size increases with no. npAFP:
1
10
20
100
50
0
Jan–Jun 2004
Jan–Jun 2006
Jan–Jun 2008
Jan–Jun 2010
Jan–Jun 2012
Jan–Jun 2014

KOGI

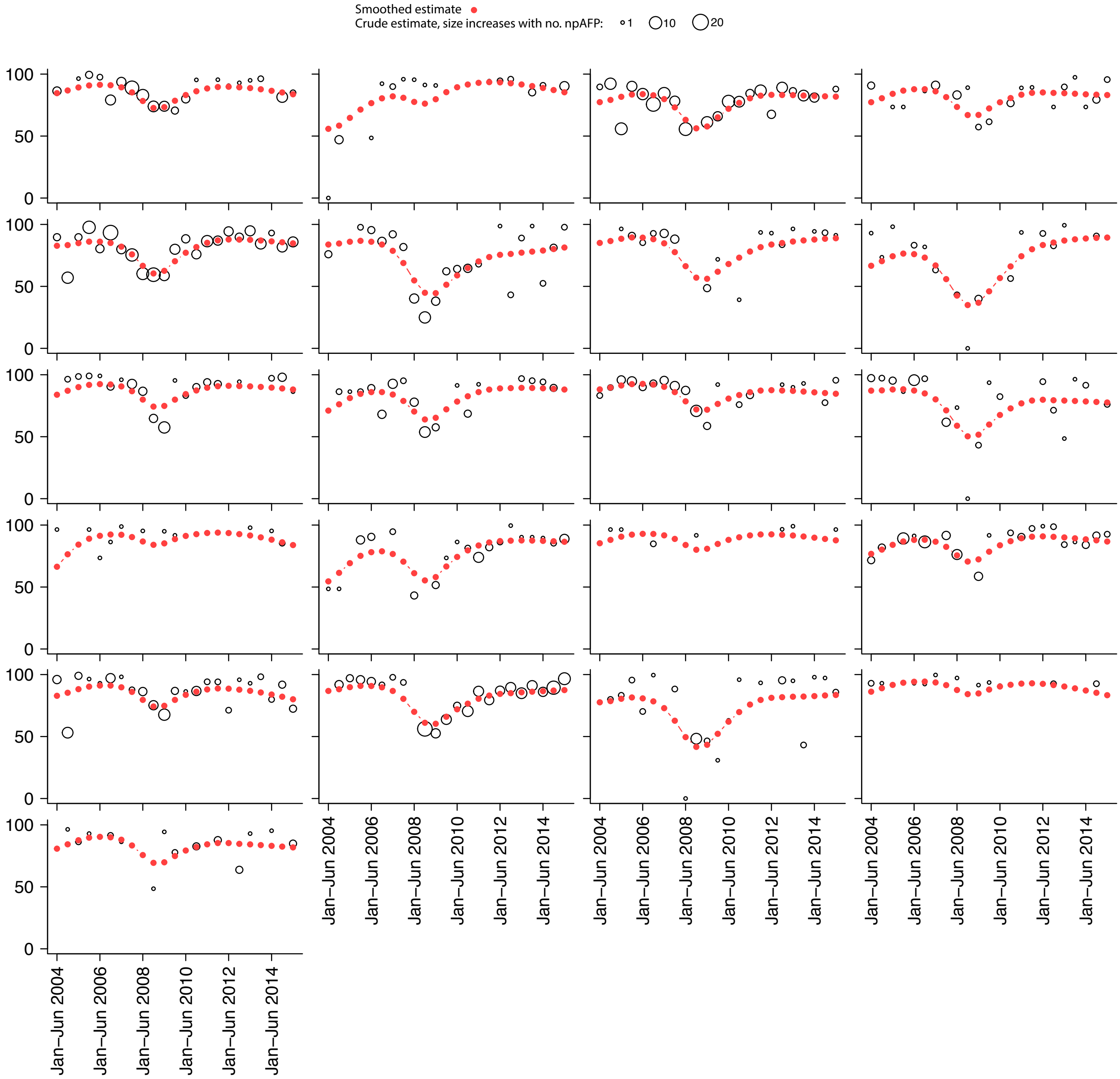
Smoothed estimate
Crude estimate, size increases with no. npAFP:
1
10
20
100
50
0
Jan–Jun 2004
Jan–Jun 2006
Jan–Jun 2008
Jan–Jun 2010
Jan–Jun 2012
Jan–Jun 2014

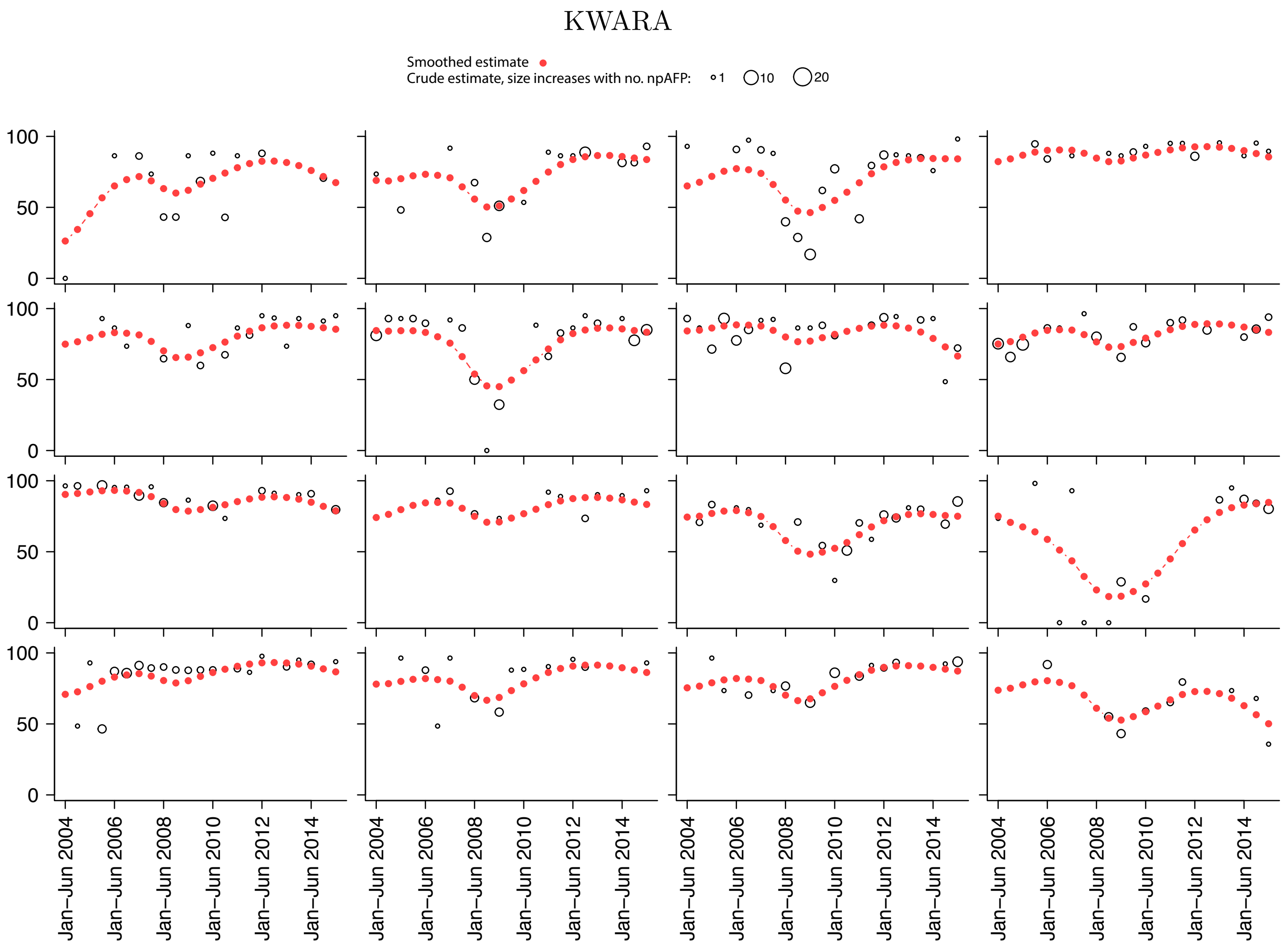
KWARA
Smoothed estimate
Crude estimate, size increases with no. npAFP: 1 10 20
100
50
0
Jan–Jun 2004
Jan–Jun 2006
Jan–Jun 2008
Jan–Jun 2010
Jan–Jun 2012
Jan–Jun 2014

LAGOS

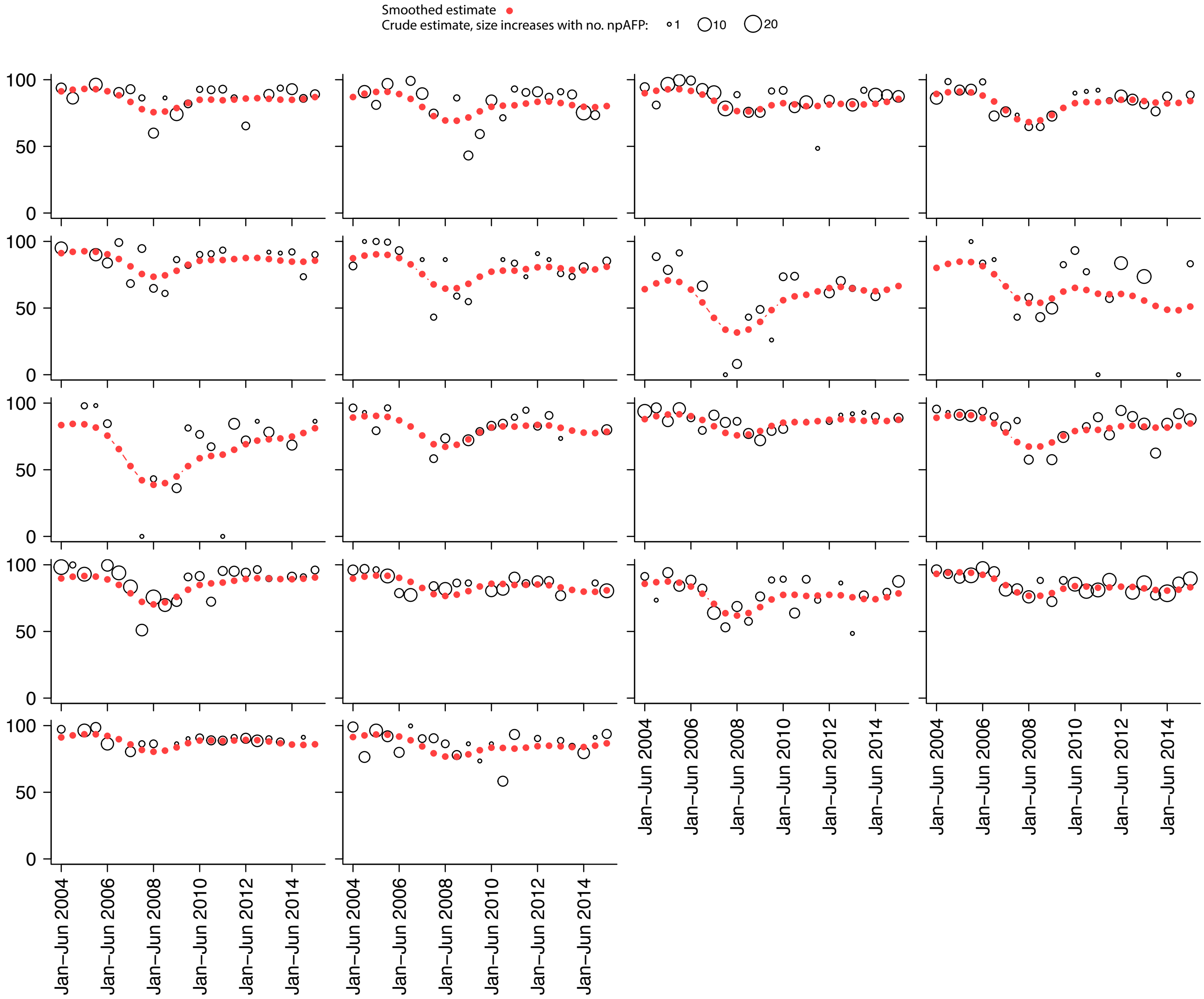

Smoothed estimate
Crude estimate, size increases with no. npAFP: 1 10 20
100
50
0
Jan–Jun 2004
Jan–Jun 2006
Jan–Jun 2008
Jan–Jun 2010
Jan–Jun 2012
Jan–Jun 2014

NASARAWA

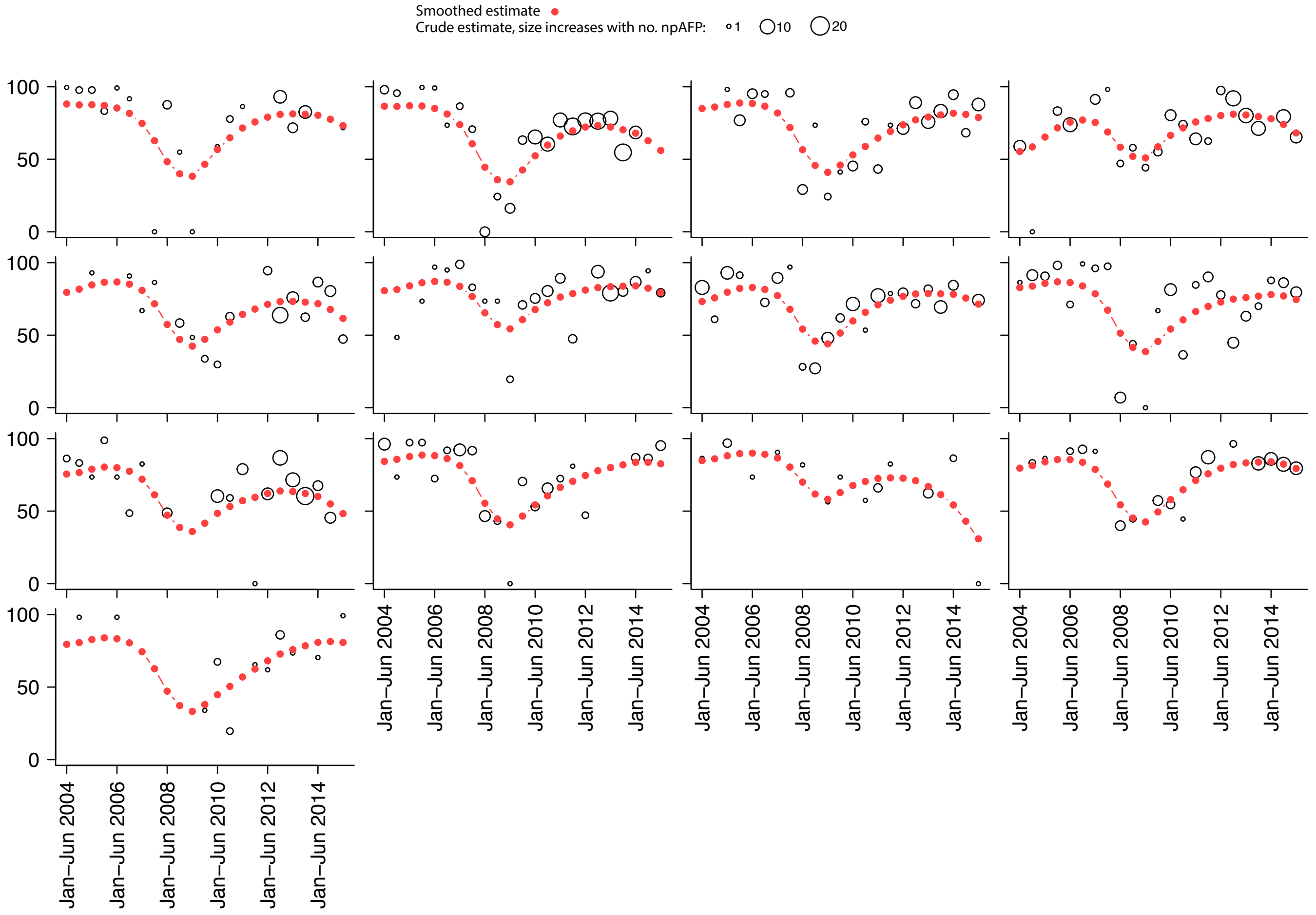
Smoothed estimate
Crude estimate, size increases with no. npAFP:
1
10
20
100
50
0
Jan–Jun 2004
Jan–Jun 2006
Jan–Jun 2008
Jan–Jun 2010
Jan–Jun 2012
Jan–Jun 2014

NIGER

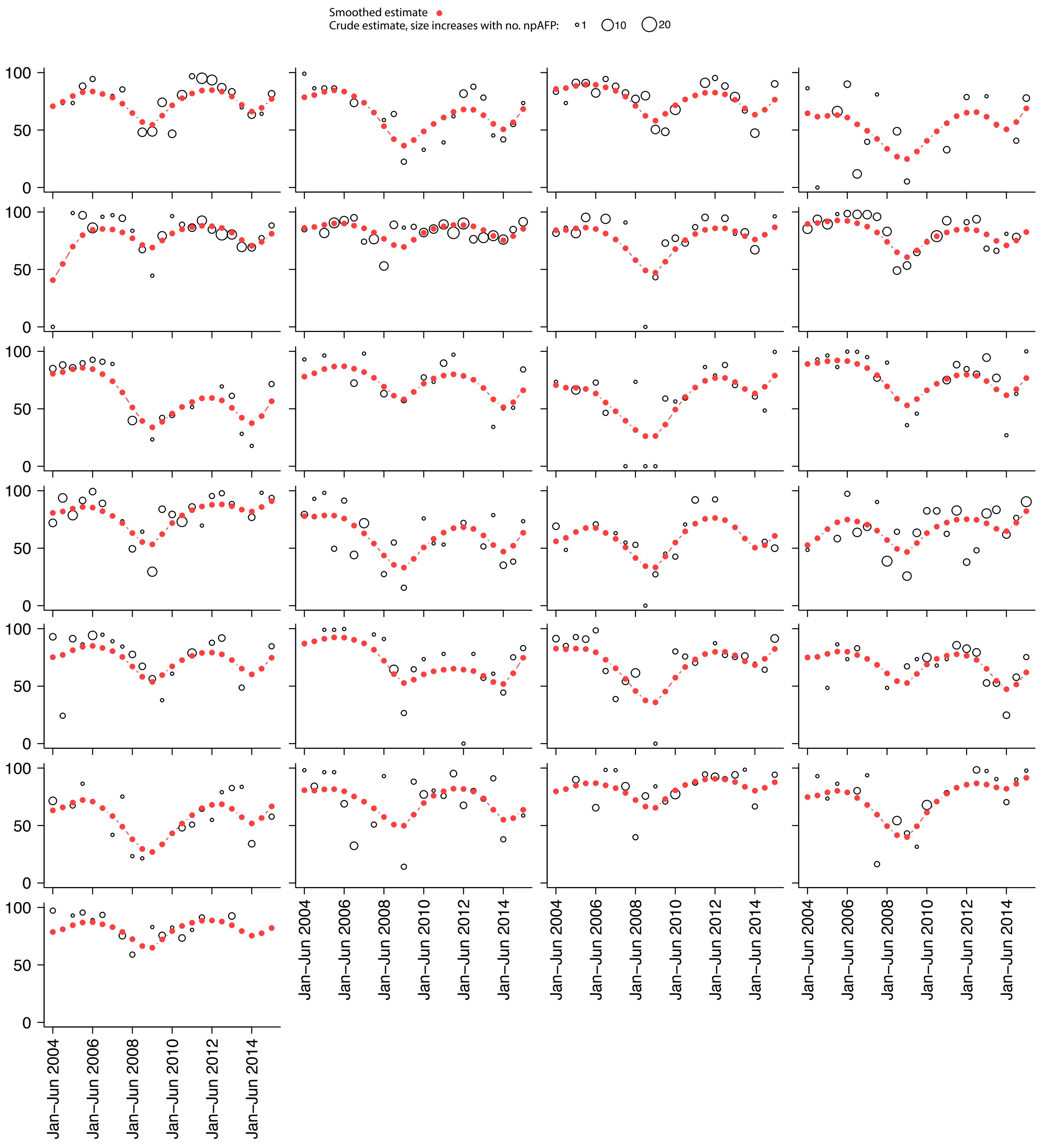

Smoothed estimate
Crude estimate, size increases with no. npAFP:
1
10
20
100
50
0
Jan–Jun 2004
Jan–Jun 2006
Jan–Jun 2008
Jan–Jun 2010
Jan–Jun 2012
Jan–Jun 2014

OGUN

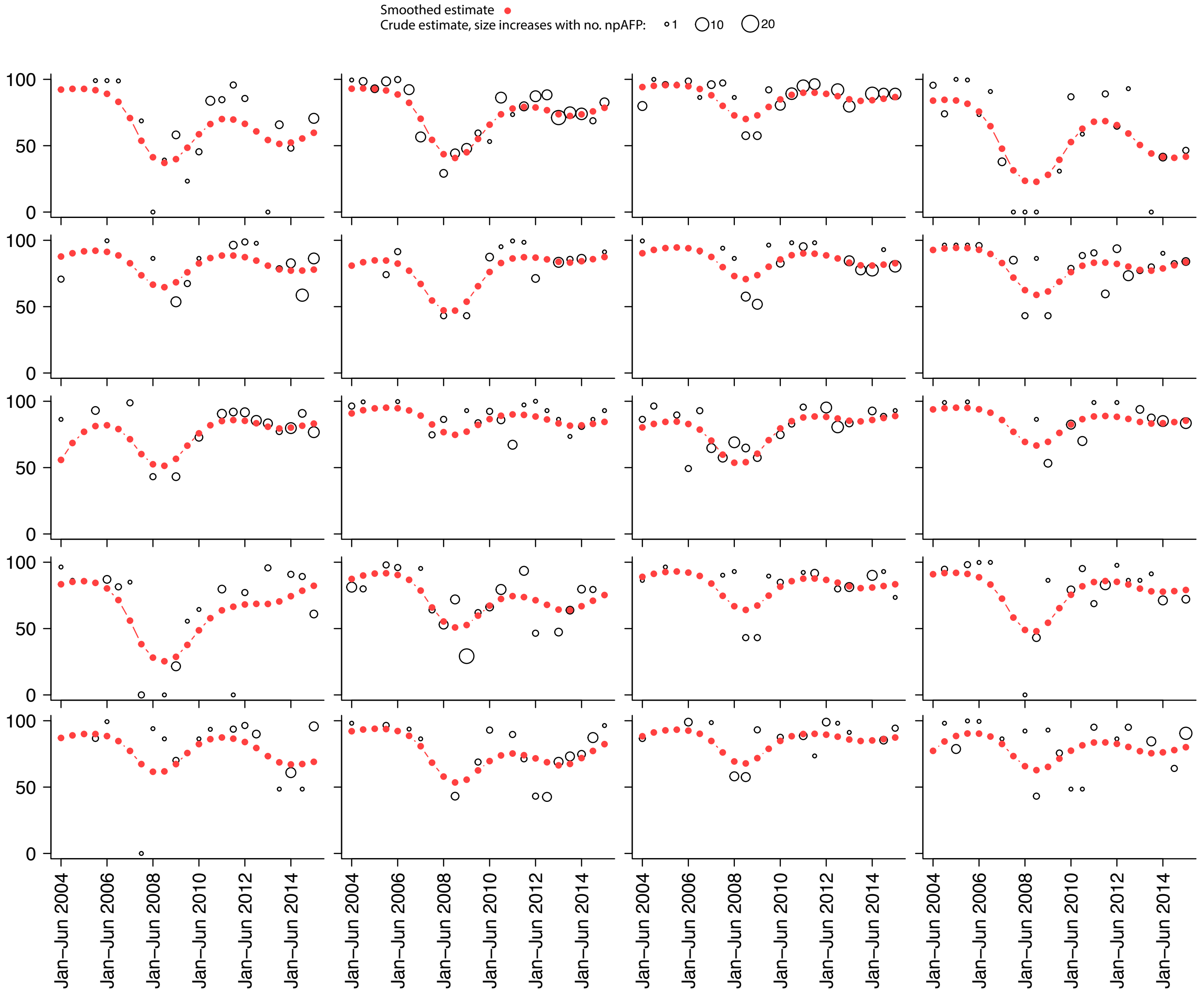

Smoothed estimate
Crude estimate, size increases with no. npAFP:
1
10
20
100
50
0
Jan–Jun 2004
Jan–Jun 2006
Jan–Jun 2008
Jan–Jun 2010
Jan–Jun 2012
Jan–Jun 2014

ONDO

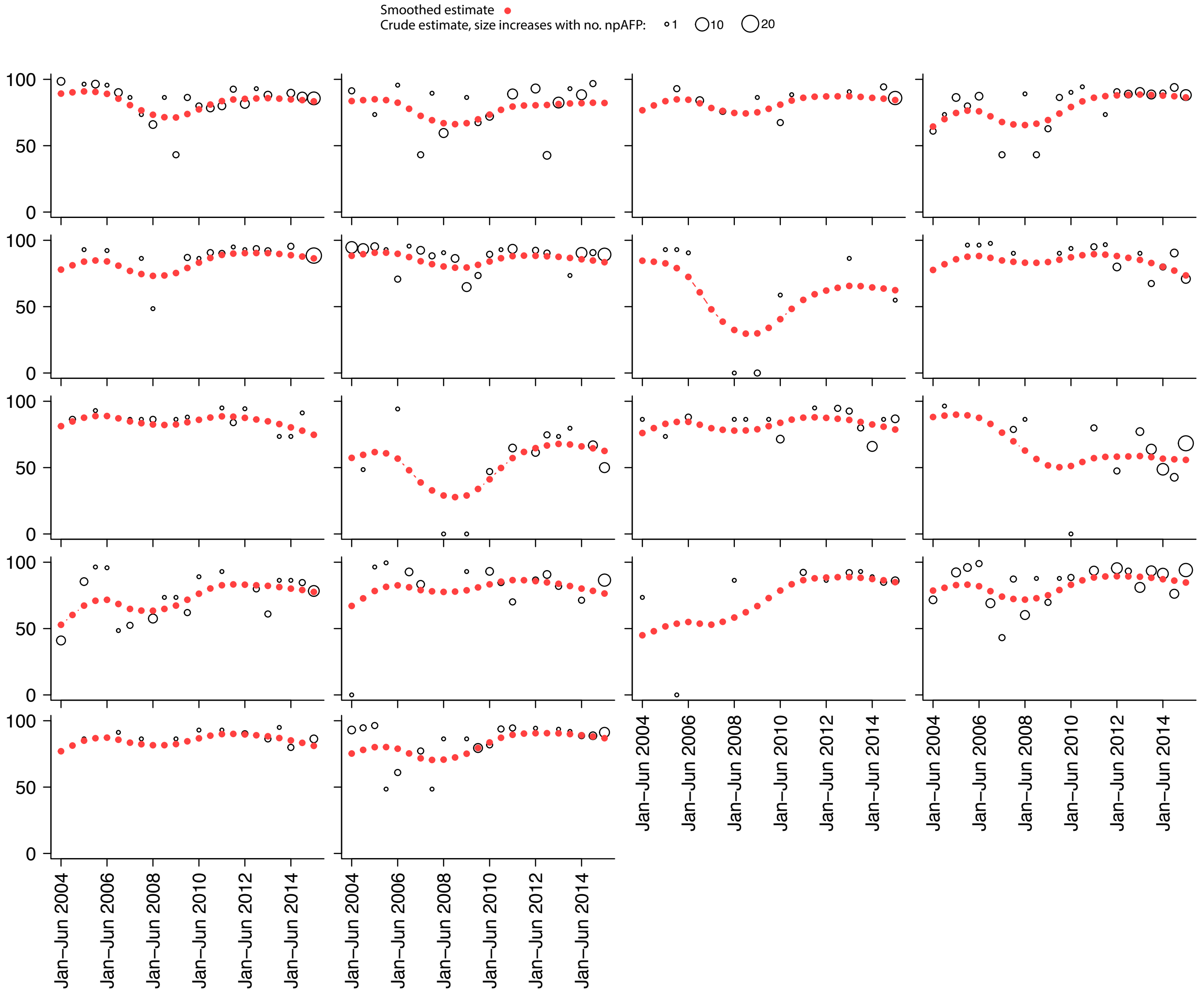
Smoothed estimate
Crude estimate, size increases with no. npAFP:
1
10
20
100
50
0
Jan–Jun 2004
Jan–Jun 2006
Jan–Jun 2008
Jan–Jun 2010
Jan–Jun 2012
Jan–Jun 2014

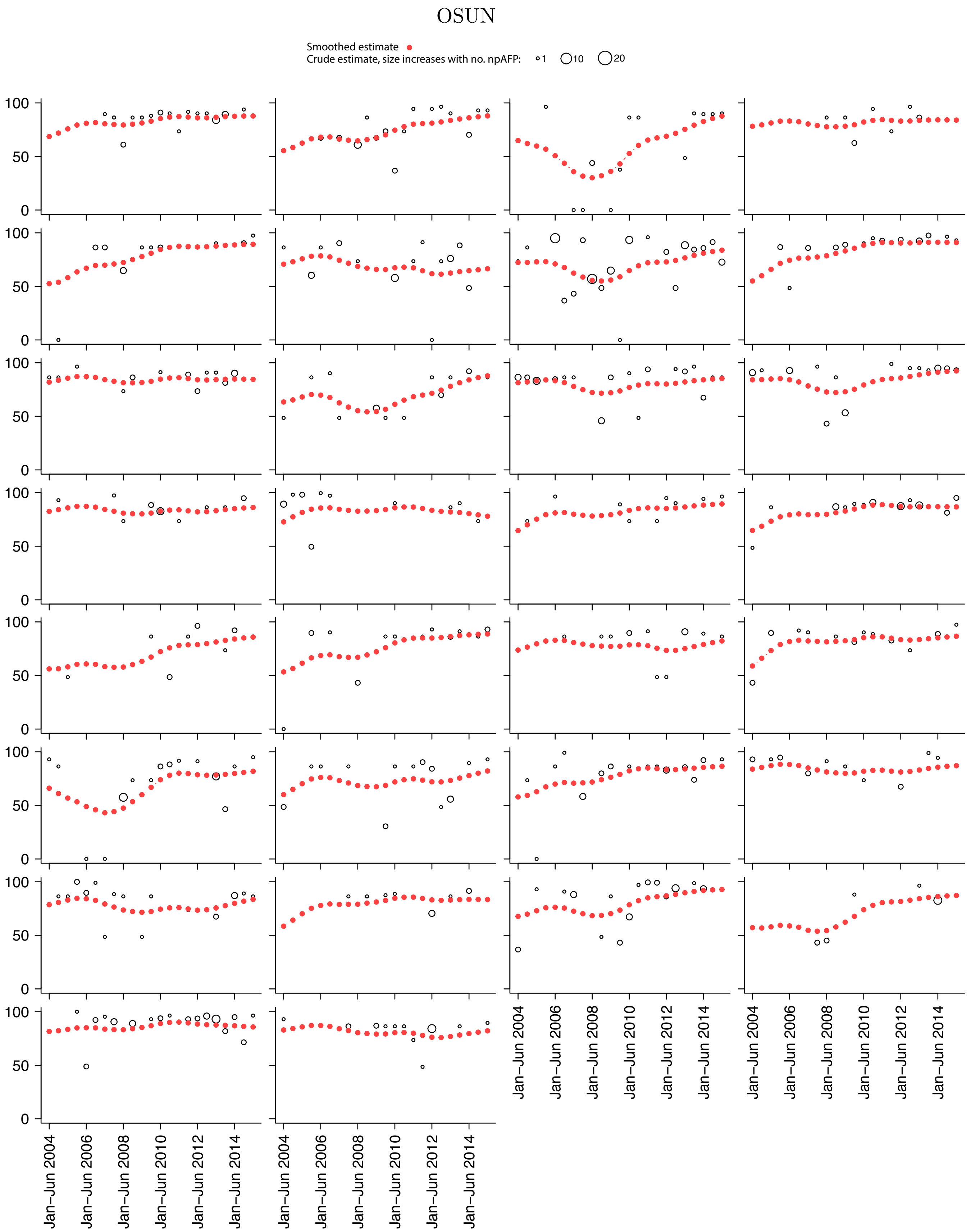

OSUN
Smoothed estimate
Crude estimate, size increases with no. npAFP:
1
10
20
100
50
0
Jan–Jun 2004
Jan–Jun 2006
Jan–Jun 2008
Jan–Jun 2010
Jan–Jun 2012
Jan–Jun 2014

OYO

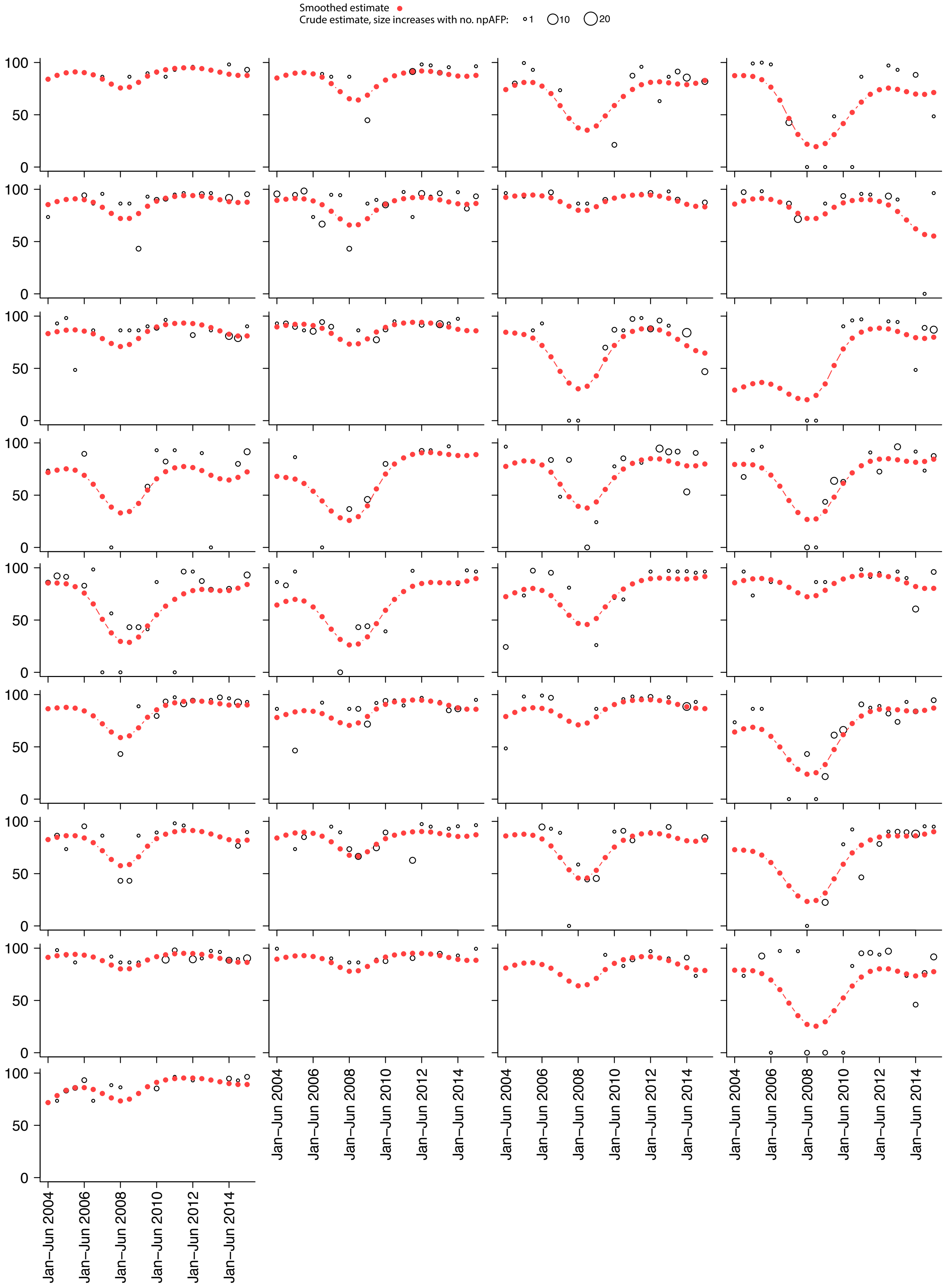
Smoothed estimate
Crude estimate, size increases with no. npAFP:
1
10
20
100
50
0
Jan–Jun 2004
Jan–Jun 2006
Jan–Jun 2008
Jan–Jun 2010
Jan–Jun 2012
Jan–Jun 2014

PLATEAU

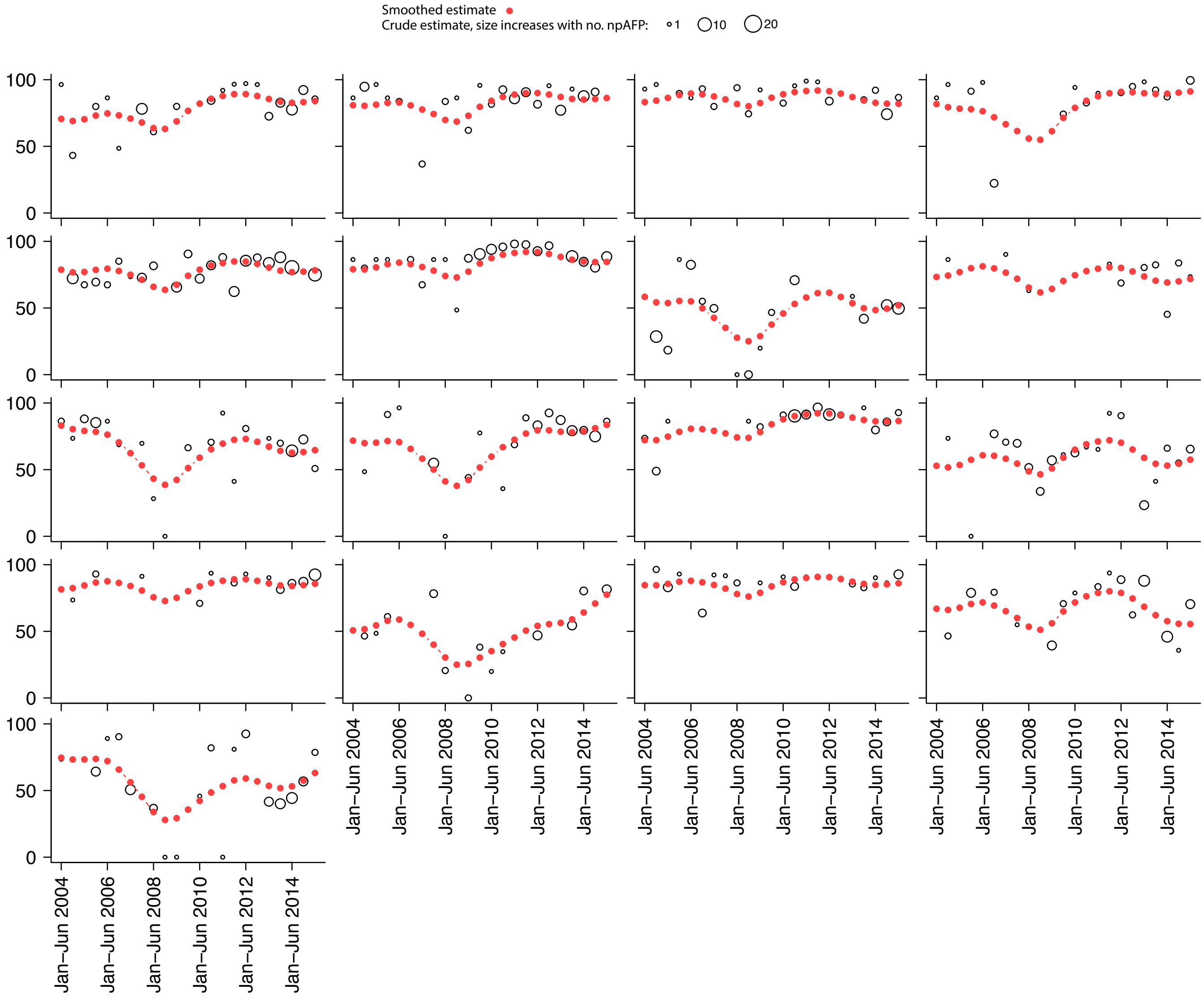
Smoothed estimate
Crude estimate, size increases with no. npAFP: 1 10 20
100
50
0
Jan–Jun 2004
Jan–Jun 2006
Jan–Jun 2008
Jan–Jun 2010
Jan–Jun 2012
Jan–Jun 2014

RIVERS

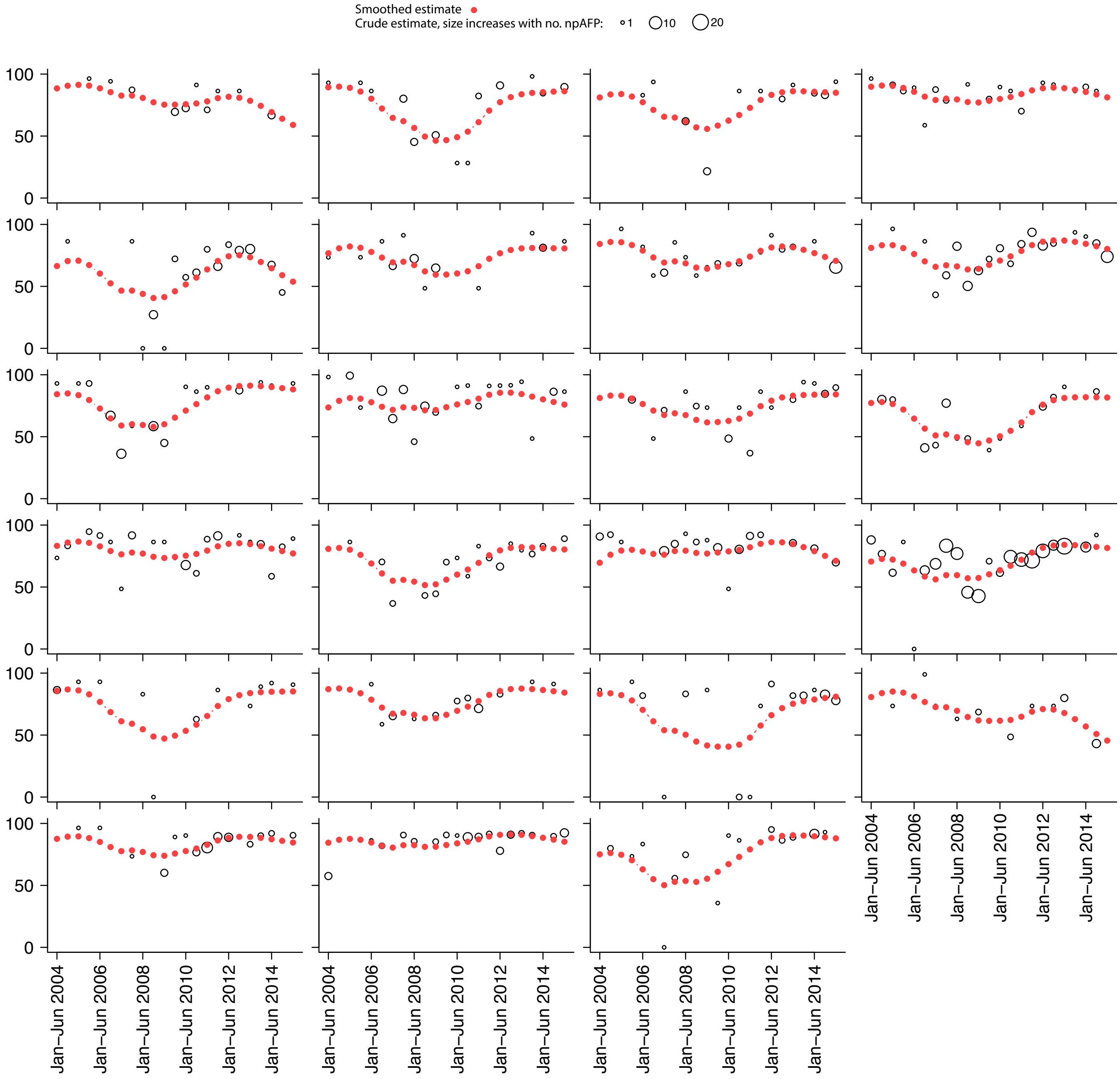

Smoothed estimate
Crude estimate, size increases with no. npAFP:
1
10
20
100
50
0
Jan–Jun 2004
Jan–Jun 2006
Jan–Jun 2008
Jan–Jun 2010
Jan–Jun 2012
Jan–Jun 2014

SOKOTO

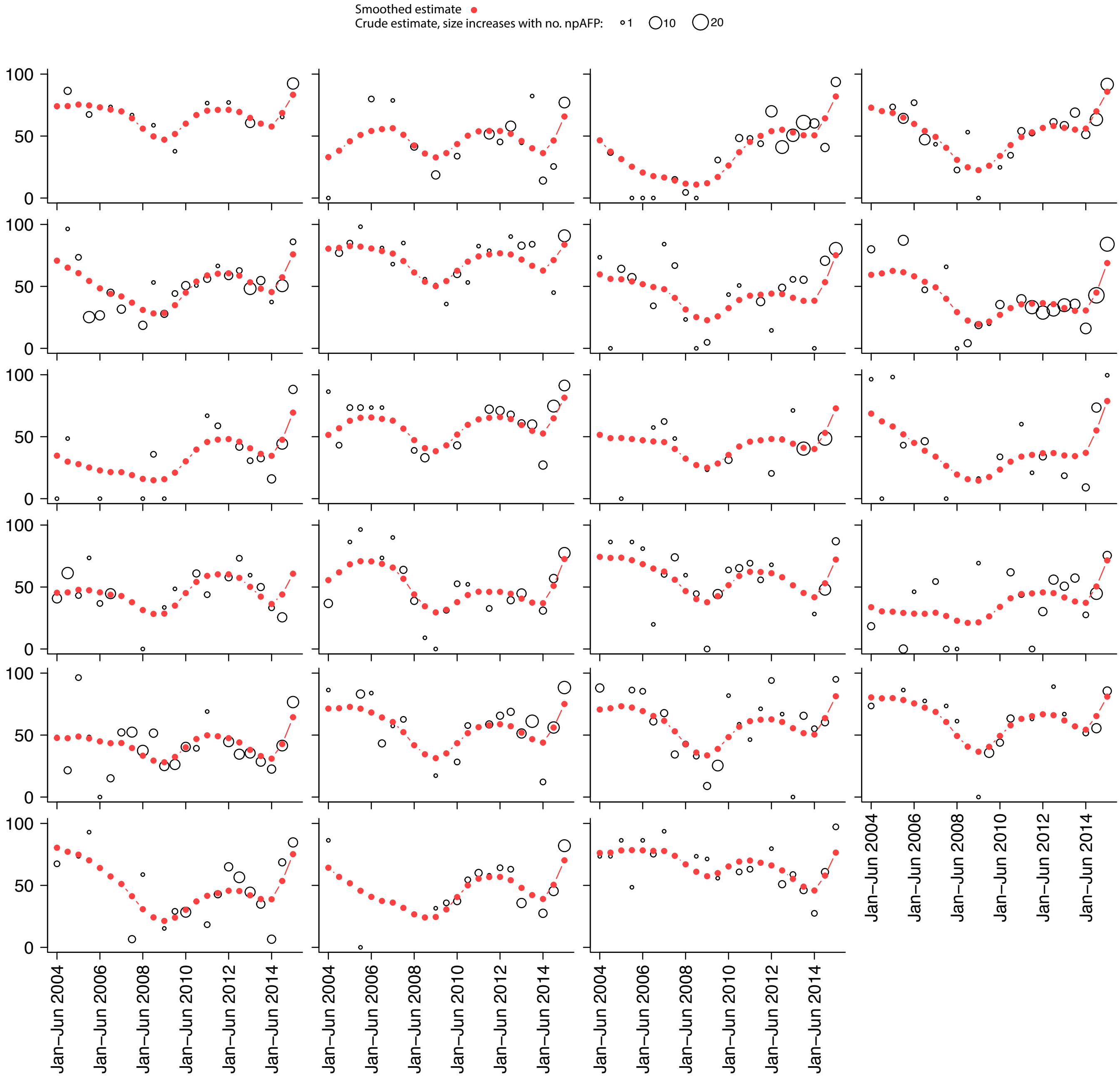
Smoothed estimate
Crude estimate, size increases with no. npAFP: 1 10 20
100
50
0
Jan–Jun 2004
Jan–Jun 2006
Jan–Jun 2008
Jan–Jun 2010
Jan–Jun 2012
Jan–Jun 2014

TARABA

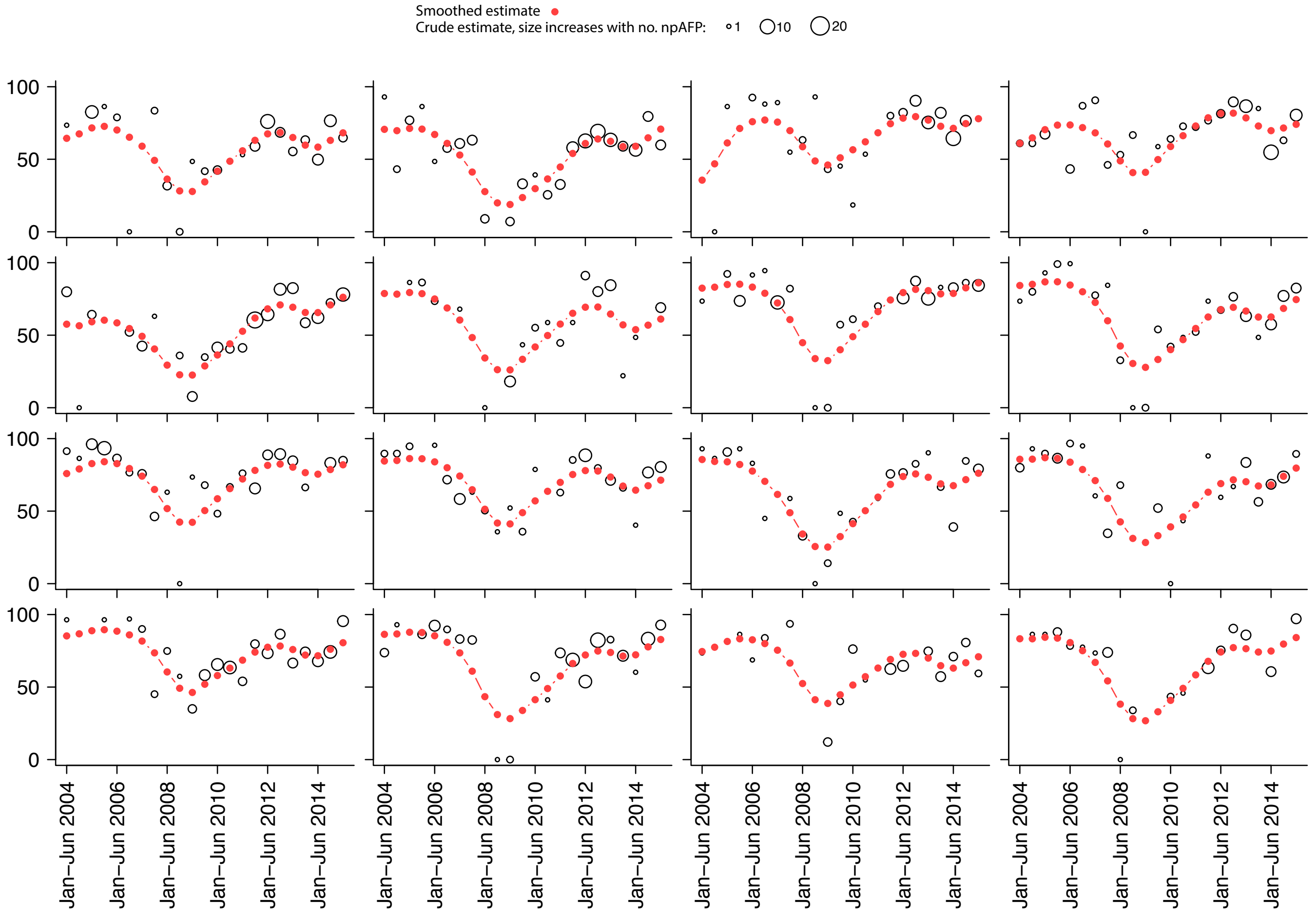
Smoothed estimate
Crude estimate, size increases with no. npAFP:
1
10
20
100
50
0
Jan–Jun 2004
Jan–Jun 2006
Jan–Jun 2008
Jan–Jun 2010
Jan–Jun 2012
Jan–Jun 2014

YOBE

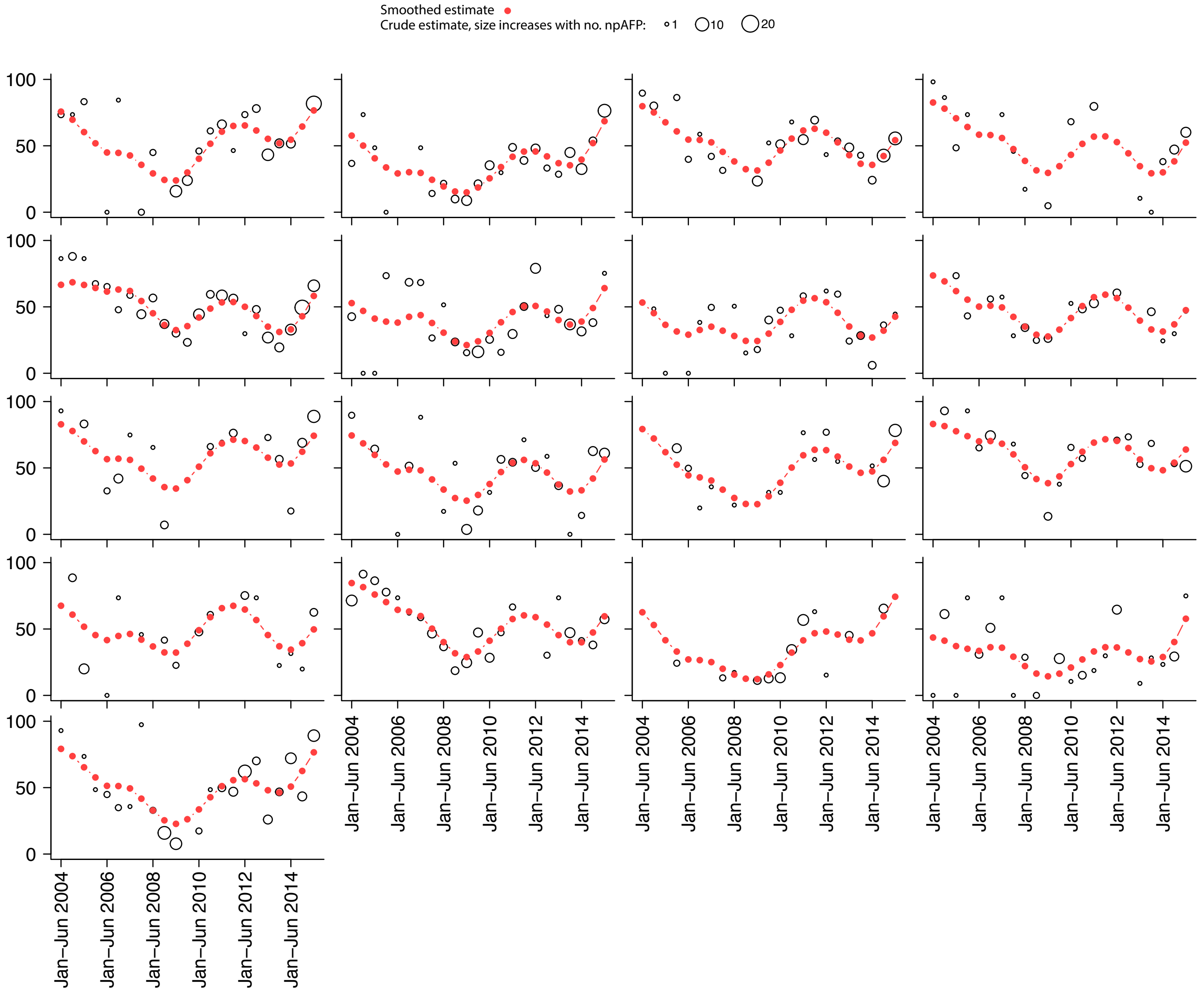

Smoothed estimate
Crude estimate, size increases with no. npAFP:
1
10
20
100
50
0
Jan–Jun 2004
Jan–Jun 2006
Jan–Jun 2008
Jan–Jun 2010
Jan–Jun 2012
Jan–Jun 2014

ZAMFARA

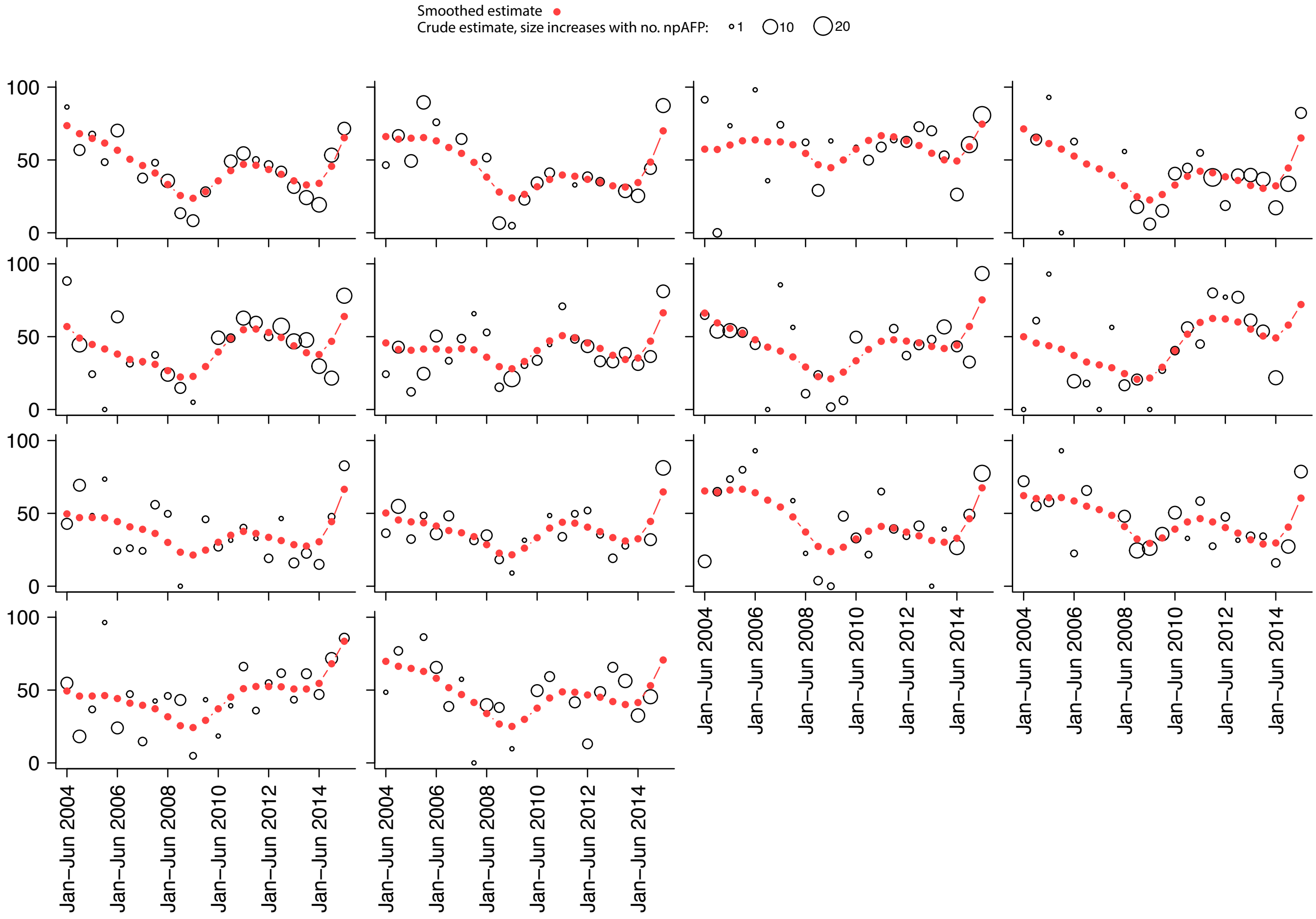
Smoothed estimate
Crude estimate, size increases with no. npAFP:
1
10
20
100
50
0
Jan–Jun 2004
Jan–Jun 2006
Jan–Jun 2008
Jan–Jun 2010
Jan–Jun 2012
Jan–Jun 2014
