# Supplementary material for: Population Immunity against Serotype-2 Poliomyelitis Leading up to the Global Withdrawal of the Oral Poliovirus Vaccine: Spatio-temporal Modelling of Surveillance Data
Source: PLoS Med. 2016 Oct 4;13(10):e1002140. doi: 10.1371/journal.pmed.1002140 (PMC5049753; doi:10.1371/journal.pmed.1002140)
Supplement: S3 Text — (PDF) [file pmed.1002140.s003.pdf]

S3 Text

Crude and estimated serotype-2 population immunity in Pakistan by district and 6-month period from Jan-Jun 2004 to Jan-Jun 2015

Figure XXXIII. Crude (black circles) and estimated (red points) serotype-2 population immunity in each district of Pakistan and 6-month period from Jan-Jun 2004 to Jan-Jun 2015 presented by state in alphabetical order. The size of the black circles indicates the number of non-polio AFP cases that informed that estimate.

AZAD JAMMU AND KASHMIR (AJK)

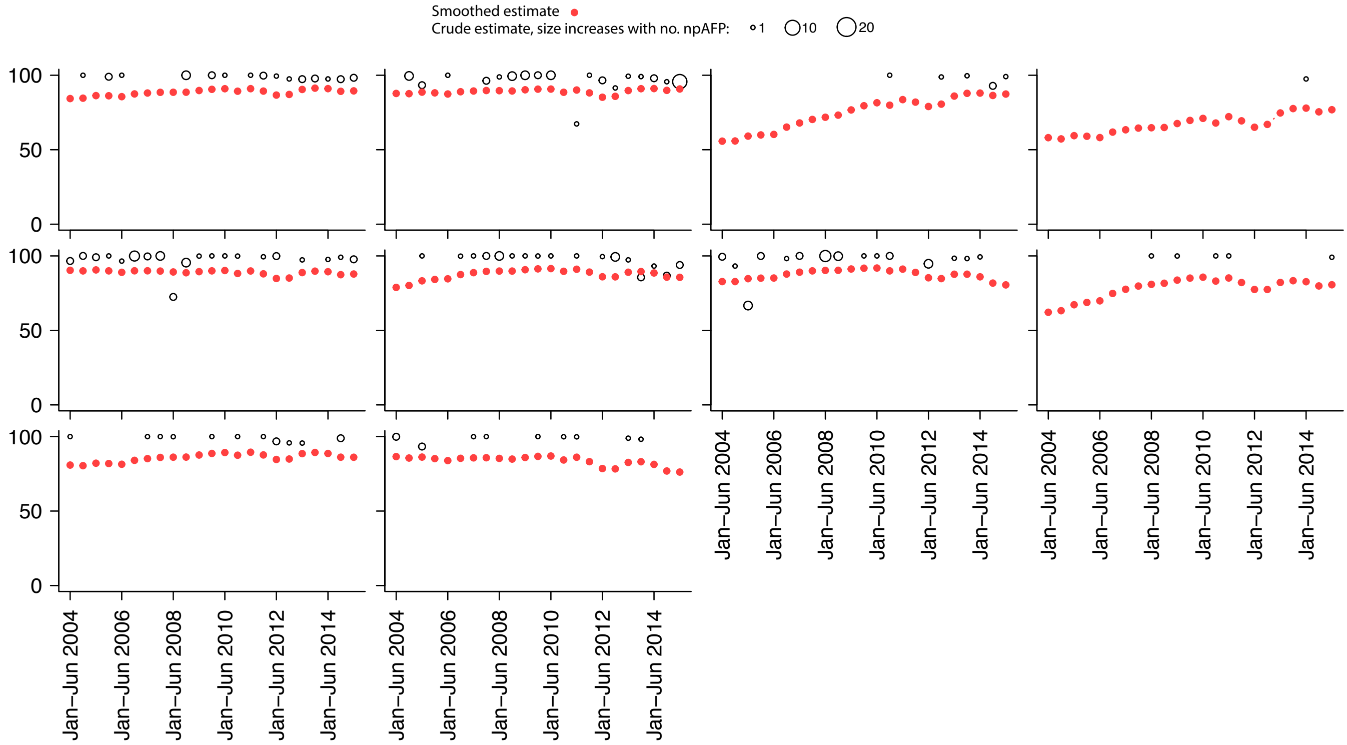

BALOCHISTAN

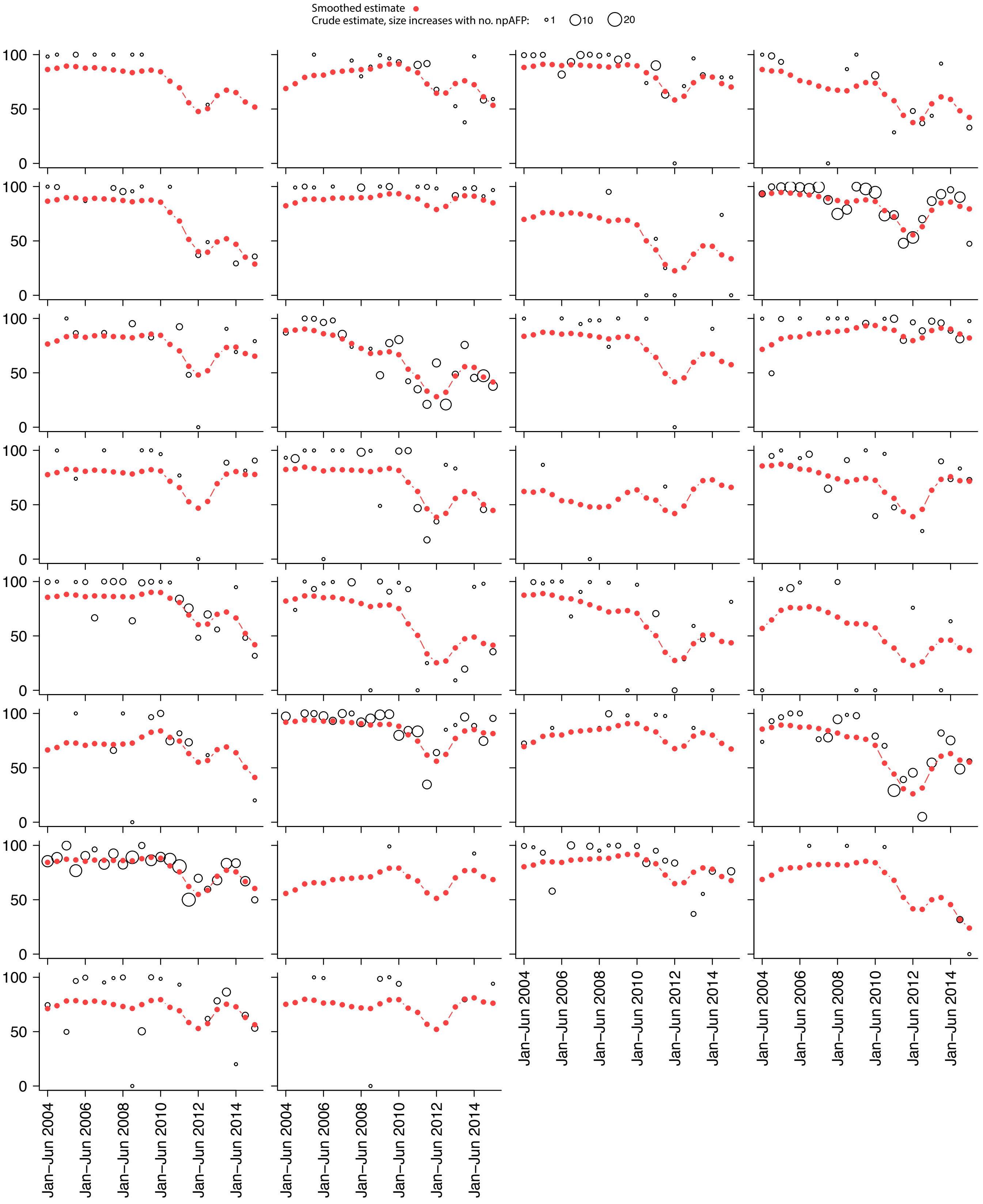

GILGIT BALTISTAN

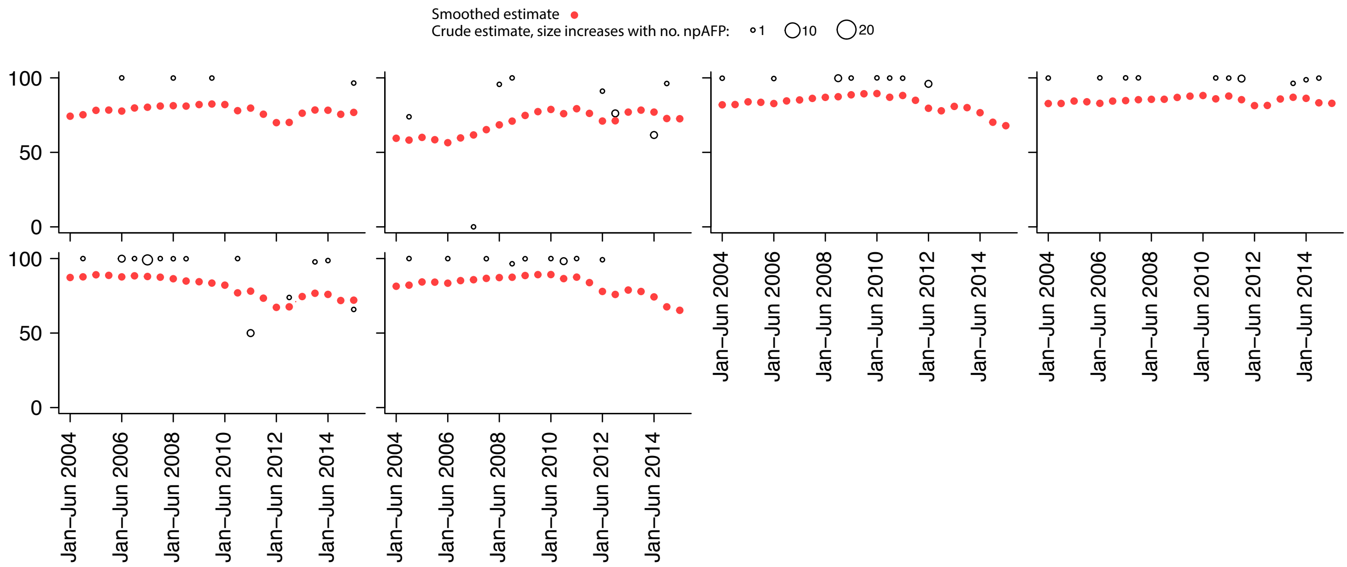

ISLAMABAD

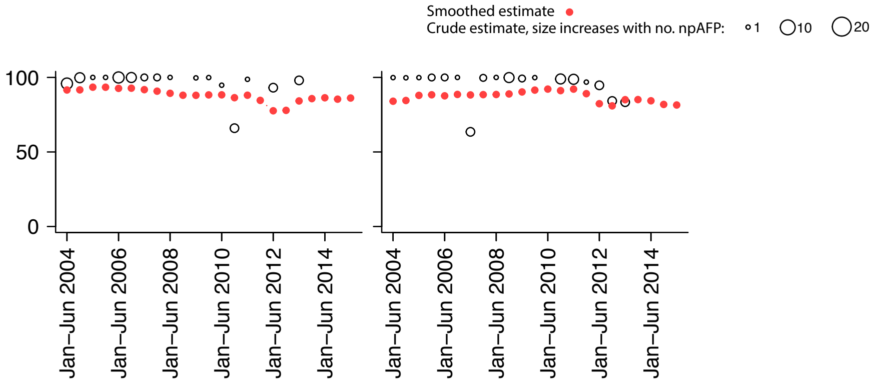

Smoothed estimate
Crude estimate, size increases with no. npAFP:
1
10
20
100
50
0
Jan–Jun 2004
Jan–Jun 2006
Jan–Jun 2008
Jan–Jun 2010
Jan–Jun 2012
Jan–Jun 2014

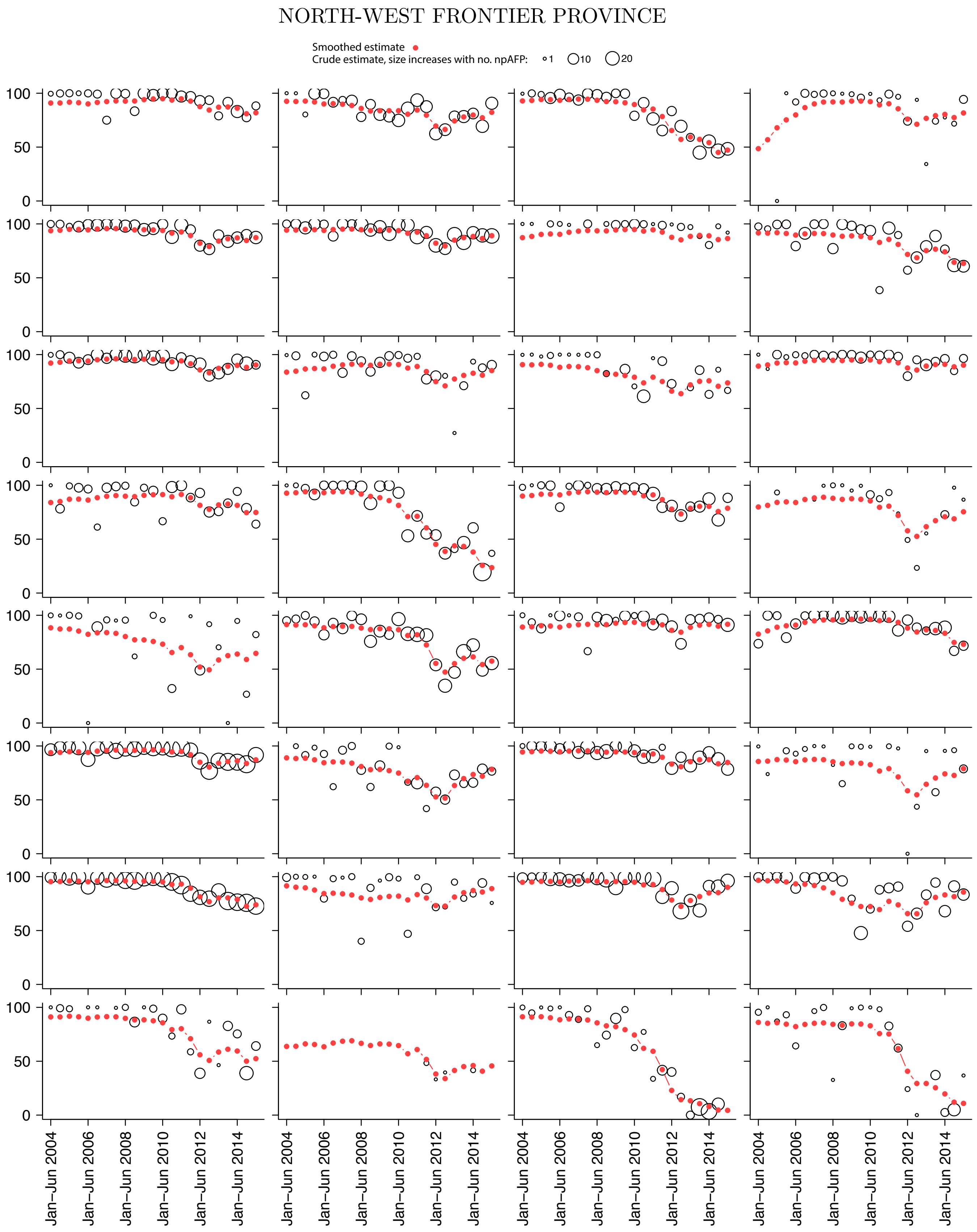
NORTH-WEST FRONTIER PROVINCE
Smoothed estimate
Crude estimate, size increases with no. npAFP:
1
10
20
100
50
0
Jan–Jun 2004
Jan–Jun 2006
Jan–Jun 2008
Jan–Jun 2010
Jan–Jun 2012
Jan–Jun 2014

PUNJAB

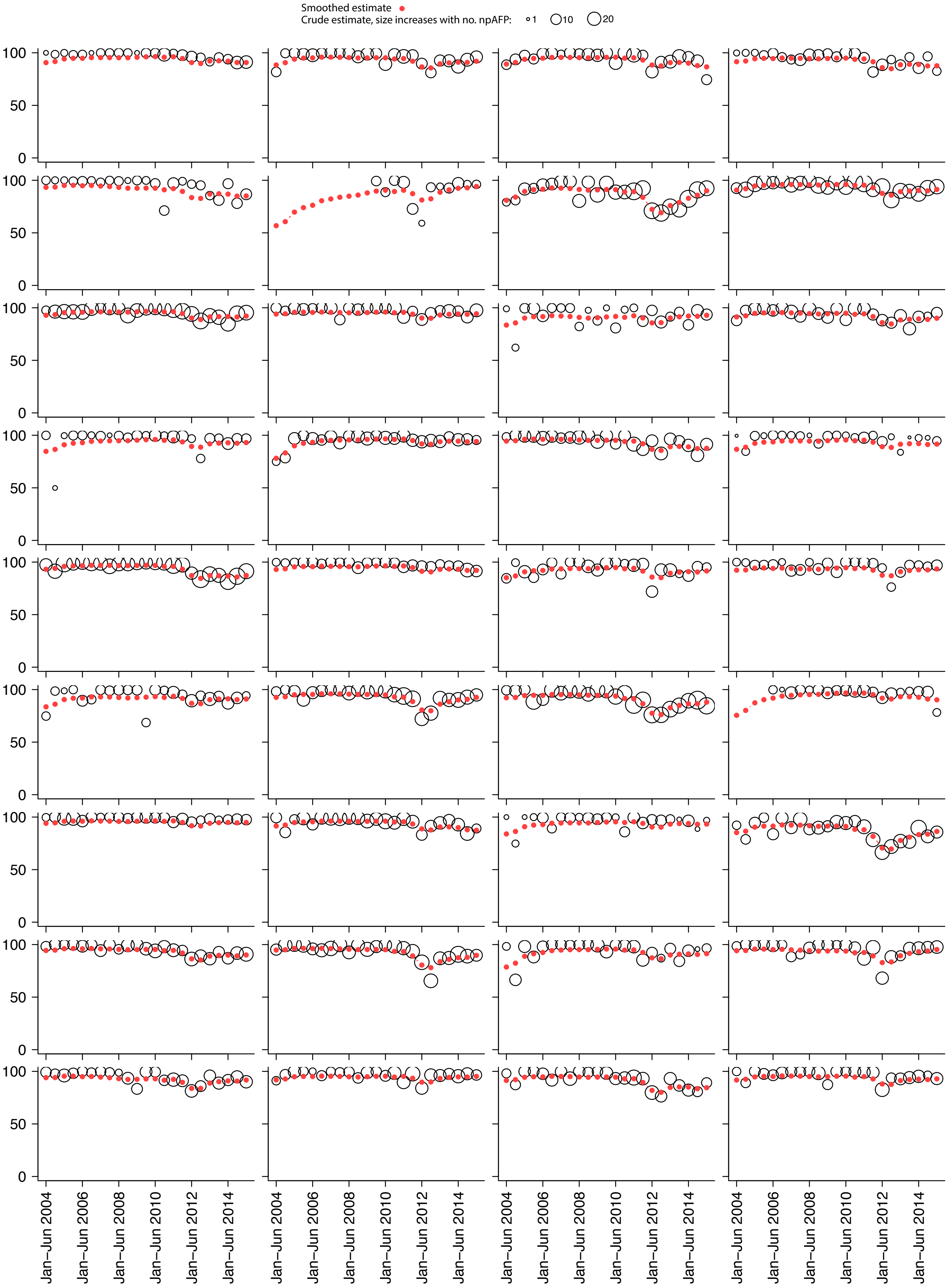

Smoothed estimate
Crude estimate, size increases with no. npAFP:
1
10
20
100
50
0
Jan–Jun 2004
Jan–Jun 2006
Jan–Jun 2008
Jan–Jun 2010
Jan–Jun 2012
Jan–Jun 2014

SINDH

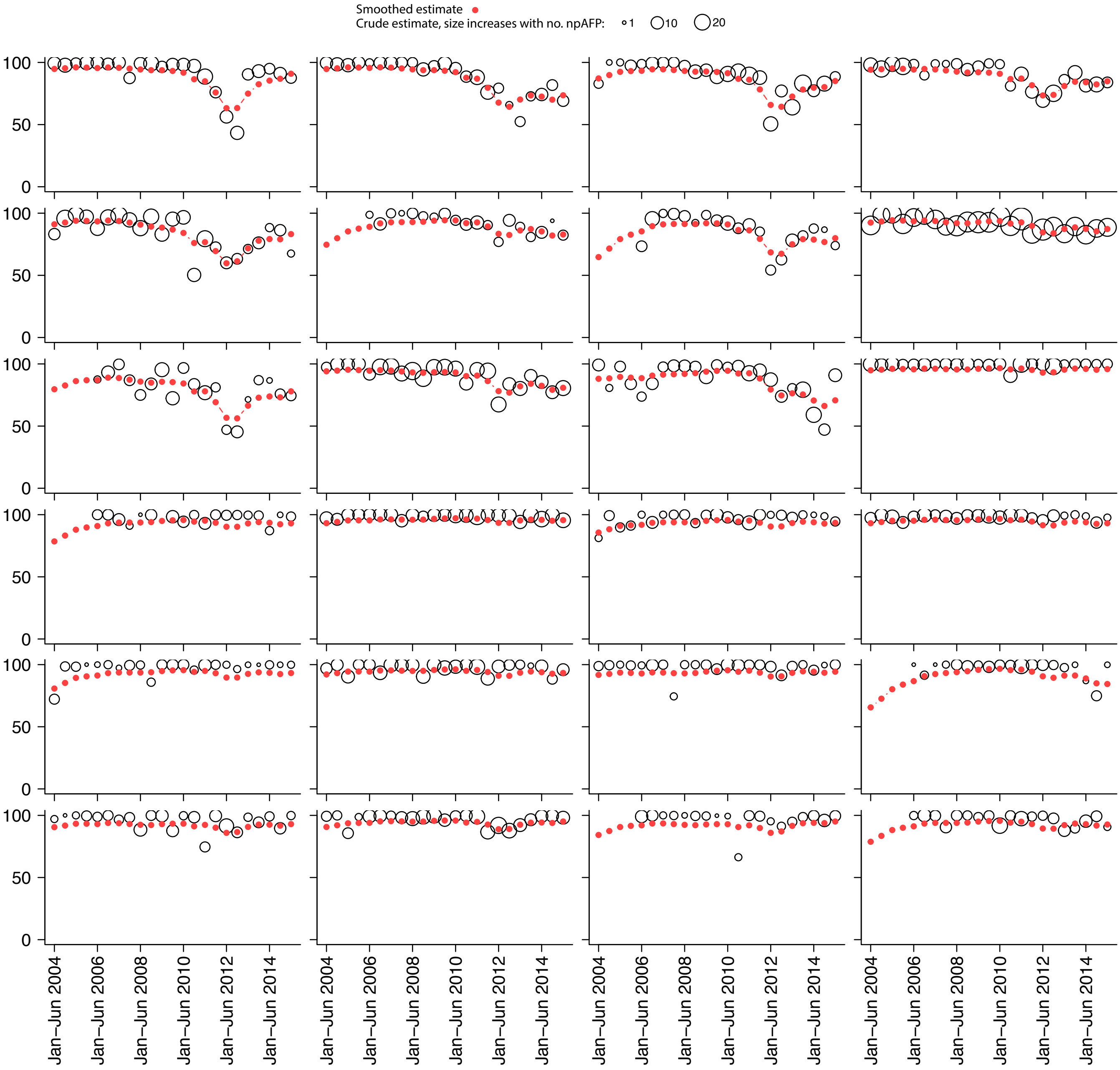
Smoothed estimate
Crude estimate, size increases with no. npAFP:
1
10
20
100
50
0
Jan–Jun 2004
Jan–Jun 2006
Jan–Jun 2008
Jan–Jun 2010
Jan–Jun 2012
Jan–Jun 2014
